# Quantifying Membrane Structure and Dynamics during Bioproduct Production in *Zymomonas mobilis* by Molecular Simulation

**DOI:** 10.1021/acs.jpcb.5c06231

**Published:** 2026-02-18

**Authors:** Nitin Kumar Singh, Josh V. Vermaas

**Affiliations:** † MSU-DOE Plant Research Laboratory, 3078 Michigan State University, 612 Wilson Road, East Lansing, Michigan 48824, United States; ‡ DOE Great Lakes Bioenergy Research Center, Michigan State University, 612 Wilson Road, East Lansing, Michigan 48824, United States; § Deparment of Biochemistry and Molecular Biology, Michigan State University, 612 Wilson Road, East Lansing, Michigan 48824, United States

## Abstract

The conversion of lignocellulosic biomass into biofuels and bioproducts by microbial biorefineries is central to a sustainable chemical industry. *Zymomonas mobilis* is one such biorefinery chassis and is resistant to ethanol stress, leading to its use in biomass conversion to biofuels and bioproducts. However, *Z. mobilis* growth is often inhibited by organic acids, aldehydes, alcohols, ketones, and amides found in biomass hydrolysate. The resulting slow growth inhibits production and as a result drives up the price for the resulting products. One hypothesis is that these molecules interact with or disrupt the bacterial membrane, triggering stress responses and hindering growth. To test this hypothesis at the molecular level, we employ all-atom molecular dynamics (MD) simulations to investigate lignocellulose-derived small molecules and their impact on a biologically relevant *Z. mobilis* membrane model. Simulations were conducted across a range of inhibitor concentrations from 0 to 2.5 mol %, analyzing key membrane properties such as area per lipid (APL), membrane thickness, lipid-order parameter (−*S*_CH_), lateral diffusion coefficient (*D*_*xy*_), and permeability coefficient (Pm). From simulation, we observed altered membrane structure and dynamics at these modest small molecule concentrations commonly found in hydrolysates. Generally, the membranes become thinner, with a higher area per lipid and lower-order parameter as the small molecule concentration increases. These trends are stronger for more hydrophobic molecules with greater hydrophobic bulk, as isobutanol, propanol, and propanoic acid showed greater membrane perturbations as the concentration increased compared to other small molecules. Tracking small molecule distributions directly in our equilibrium simulations allows us to determine concentration-dependent free energy profiles for these molecules. While the trends are noisy, generally the barriers to crossing the membrane decrease as the concentration increases, indicating that the membranes become leakier as small molecule concentrations rise. Comparing between native *Z. mobilis* membranes with hopanoids and membranes sharing the same phospholipid composition but without hopanoids, hopanoids stabilize and order the membrane for smaller molecules to maintain membrane structure but appear insufficient for larger hydrophobic molecules like isobutanol. These findings provide a mechanistic understanding of how small molecules found in biomass degradation streams interact with the *Z. mobilis* membrane, offering valuable insights for future strain engineering efforts to optimize biofuel and bioproduct synthesis from biomass feedstocks by highlighting limits to small molecule tolerance. This knowledge can guide the modification of membrane composition to develop more robust microbes, thereby improving microbial survival and yields in industrial contexts.

## Graphical abstract

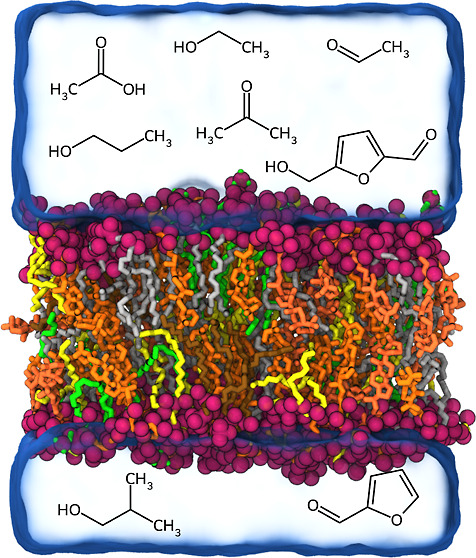

## Introduction

Global efforts toward sustainable and renewable energy and materials have intensified research efforts into converting lignocellulosic biomass from plant or microbial sources, including agricultural or forestry wastes, into biofuels and bioproducts.^1–3^ These plant residues are first mechanically and/or chemically pretreated to yield hydrolysates that are a mixture of many compounds. Rather than depend on separation technologies to feed individual waste streams, the biorefinery concept^4,5^ uses the flexible metabolic pathways found in microbes to biologically funnel these small molecules into specific fuel or product chemicals of commercial interest. However, microbial conversion is challenged by the presence of inhibitory compounds generated during pretreatment and hydrolysis.^6–10^ These inhibitors, such as furan aldehydes (e.g., furfural, 5-hydroxymethylfurfural (HMF)), weak organic acids (e.g., acetic acid), and phenolic compounds, significantly hinder the growth and metabolic activity of fermenting microorganisms.^6,11^

Among potential biorefinery platform microorganisms, *Zymomonas mobilis*,^12–16^ a natural ethanologenic bacterium distinguished by its high ethanol productivity, ethanol tolerance, and unique Entner–Doudoroff (ED) pathway, has emerged as a leading candidate for industrial biofuel production.^17–21^ Recent studies have established *Z. mobilis* as a model organism for biofuel synthesis due to its streamlined genome,^22^ well-characterized metabolic network, and genetic tractability.^23^ The ED pathway, which operates under anaerobic conditions, enables near-theoretical ethanol yields with minimal biomass production,^24^ making *Z. mobilis* exceptionally efficient compared to traditional yeast systems.^19,25^ Despite its advantages and tolerance to some solvent stressors, *Z. mobilis* remains highly sensitive to lignocellulosic hydrolysate inhibitors, which disrupt cellular integrity, impair enzymatic activity, and suppress sugar utilization, ultimately limiting scaling^26–29^ for fuel compounds like ethanol^30^ or isobutanol.^31,32^ These compounds induce oxidative stress, destabilize membranes, and inhibit glycolysis, with synergistic effects exacerbating toxicity in complex hydrolysates.^30^

While genetic approaches and wide-scale screenings can improve resistance to specific small molecules,^33–35^ the mechanism by which these small molecules interfere with growth remains unclear, which hinders directed bioengineering to solve these growth and production bottlenecks in *Z. mobilis*. One potential mechanistic hypothesis is that some small molecules alter the membrane structure or dynamics in *Z. mobilis* at low concentrations, triggering stress responses or potentially inducing membrane rupture, analogous to what has been observed for other microbes.^36,37^ Molecular simulation through classical molecular dynamics (MD) simulations offer a powerful tool to test the impact of small molecules on membrane structure or dynamics, providing high-resolution, time-resolved insights into the structural and functional changes induced by inhibitors.^38^ By simulating the interactions between *Z. mobilis* membranes and inhibitory compounds, MD simulations enable us to explore binding affinities, membrane perturbations, and potential mechanisms of tolerance that may be difficult to directly probe through other means.

In this study, we employ MD simulations to investigate the molecular interactions of *Z. mobilis* membrane models with key lignocellulosic inhibitors, including ethanol, furfural, HMF, and acetic acid (Figure 1). Our goal of this work is to elucidate the structural and dynamic basis of inhibitor tolerance and identify potential targets for strain improvement. By leveraging computational modeling, we can directly identify changes to membrane structure and dynamics induced by the presence of low inhibitor concentrations on *Z. mobilis* membranes, highlighting which compounds have the greatest perturbative effect. These simulations probe intrinsic membrane partitioning tendencies under simplified conditions and do not capture the full metabolic interconversion present during active fermentation. We can also ask hypothetical questions that are difficult to address experimentally by simulating equivalent membranes without the hopanoid compounds that are so prevalent in *Z. mobilis* membranes. These findings can inform future engineering efforts aimed at developing more resilient *Z. mobilis* strains for efficient microbial biorefinery production from lignocellulosic biomass.

**Figure 1:**
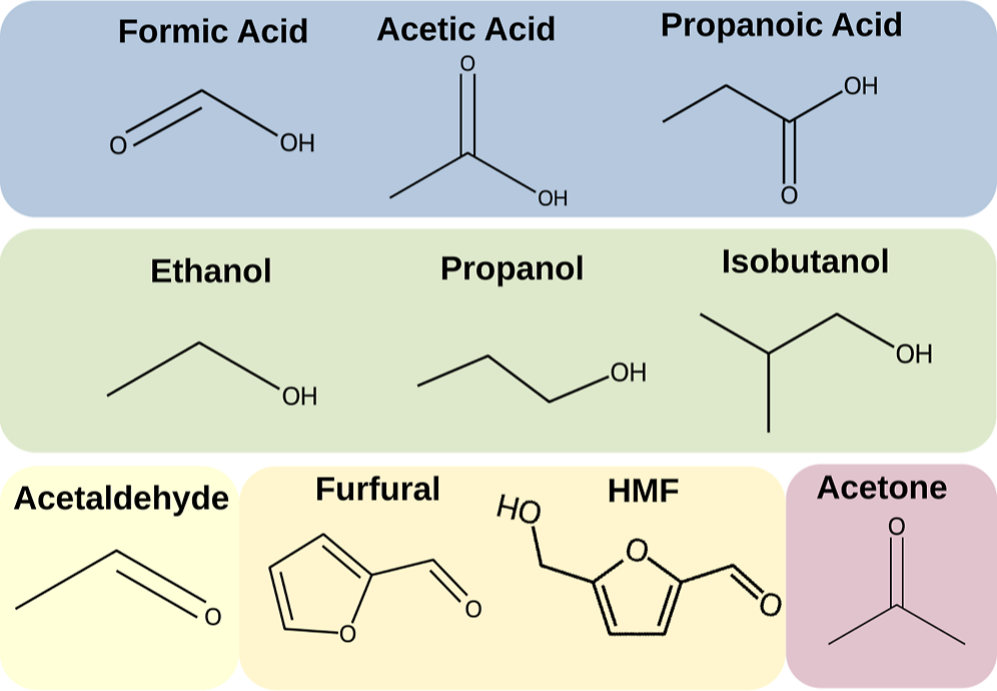
Small molecules considered in this study, grouped by chemical functionality. Blue highlights carboxylic acids (formic acid, acetic acid, and propanoic acid) in their protonated state, green includes alcohols (ethanol, propanol, and isobutanol), yellow contains aldehydes and furan derivatives (acetaldehyde, HMF, and furfural), and mauve represents a ketone (acetone).

## Methods

The general approach is to run classical molecular dynamics simulations for *Z. mobilis* membranes in increasing concentrations of small molecules that might perturb the membrane structure and would be observed in real hydrolysates. We also simulate a hypothetical membrane model for *Z. mobilis* that lacks any hopanoids in order to compare and contrast the impact these small molecules have on membrane structure and dynamics when exposed to multiple stresses.

### Membrane Model and System Preparation

The *Z. mobilis* membrane model was constructed from the phospholipids phosphatidylethanolamine (PE), phosphatidylglycerol (PG), phosphatidylcholine (PC), and cardiolipin (CL), alongside hopanoids (bacteriohopanetetrol cyclitol ether and bacteriohopanetetrol glucosamine), as illustrated in Figure 2. The composition of the model was based on recent lipidomics data,^31^ condensing the multitude of lipids identified by lipidomics into a representative lipid mixture as detailed in Table 1. Each membrane leaflet contained 100 molecules, consisting of either a mixture of phospholipids and hopanoids or phospholipids alone. For the hypothetical hopanoid-deficient membrane system, the membrane was generated using the CHARMM-GUI Membrane Builder.^39^ Each leaflet contained 100 phospholipid molecules (combining PE, PG, PC, and CL), utilizing double the quantities listed in Table 1 to compensate for the absence of hopanoids.

**Figure 2:**
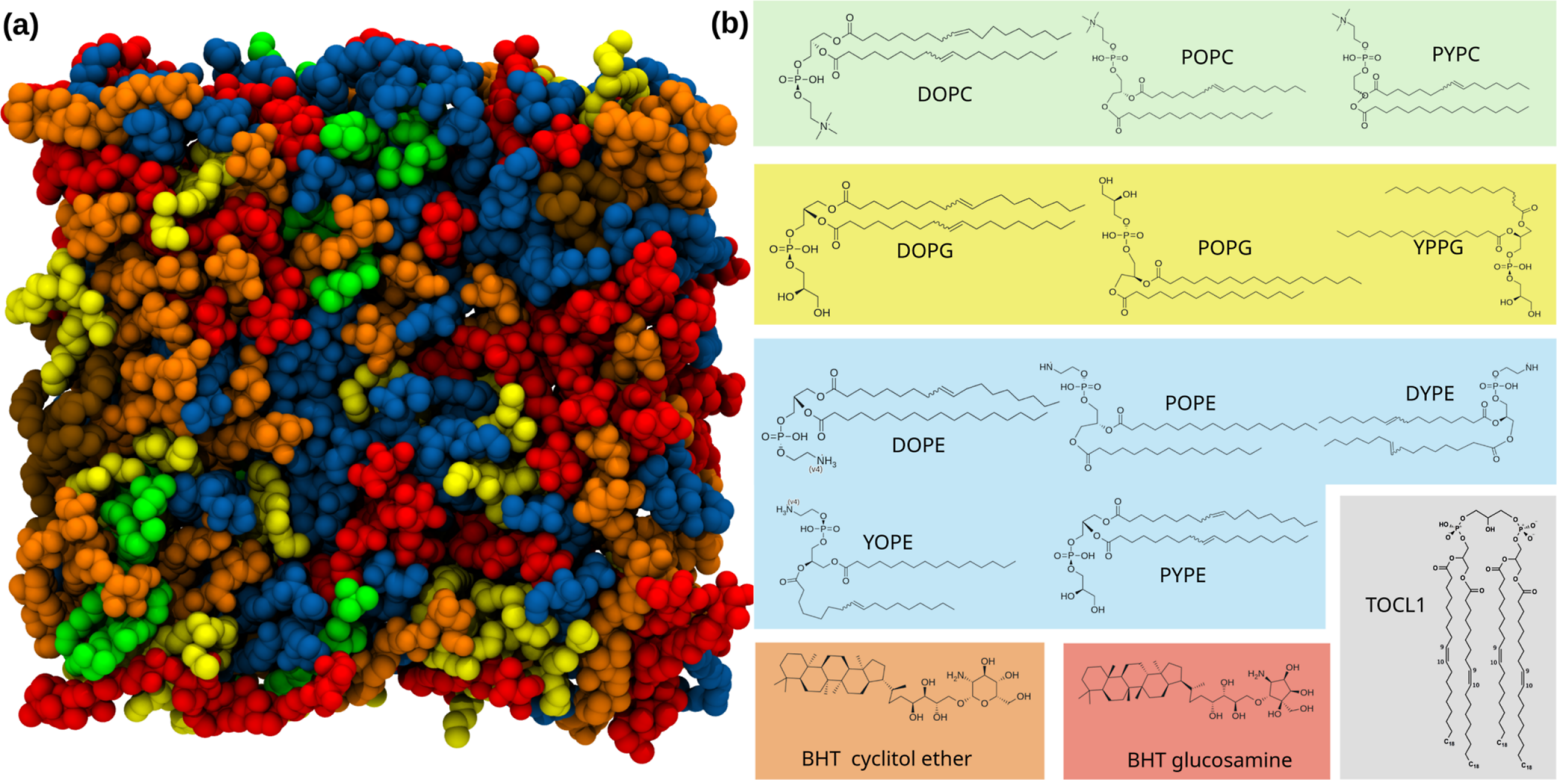
(a) Top view of the *Z. mobilis* model membrane structure. The membrane is comprised of phosphatidylcholines (PCs) in green, phosphatidylglycerols (PGs) in yellow, phosphoethanolamines (PEs) in blue, cardiolipin (TOCL1) in gray, and hopanoids in orange and red color. (b) The chemical structures of the lipids, colored according to their head groups. The names for these molecules are given in Table 1.

**Table 1:** Membrane Phospholipid and Hopanoid Composition in Wild-Type *Z. mobilis* Adapted from Available Lipidomics^31^ to Specify the Number of Lipids in Each Leaflet of the Lipid Bilayer

lipid	name	numbers
DOPE 18:1/18:1	1,2-dioleoyl-*sn*-glycero-3-phosphoethanolamine	18
PYPE 16:0/16:1	1-palmitoyl-2-arachidonoyl-*sn*-glycero-3-phosphoethanolamine	4
YOPE 16:1/18:1	1-stearoyl-2-oleoyl-*sn*-glycero-3-phosphoethanolamine	3
POPE 16:0/18:1	1-palmitoyl-2-oleoyl-*sn*-glycero-3-phosphoethanolamine	3
DYPE 16:1/16:1	1,2-di(9*Z*-hexadecenoyl)-*sn*-glycero-3-phosphoethanolamine	1
DOPG 18:1/18:1	1,2-dioleoyl-*sn*-glycero-3-phospho-(1′-rac-glycerol)	8
POPG 16:0/18:1	1-palmitoyl-2-oleoyl-*sn*-glycero-3-phospho-(1′-rac-glycerol)	4
YPPG 16:1/16:0	1-palmitoleoyl-2-palmitoyl-*sn*-glycero-3-phospho-(1′-rac-glycerol)	1
DOPC 18:1/18:1	1,2-dioleoyl-*sn*-glycero-3-phosphocholine	4
POPC 16:0/18:1	1-palmitoyl-2-oleoyl-*sn*-glycero-3-phosphocholine	1
PYPC 16:0/16:1	1-palmitoyl-2-palmitoleoyl-*sn*-glycero-3-phosphocholine	1
TOCLI 18:1/18:1/18:1/18:1	1′, 3′-bis(1,2-dioleoyl-*sn*-glycero-3-phospho)-*sn*-glycerol	2
	Bacteriohopanetetrol cyclitol ether	25
	Bacteriohopanetetrol glucosamine	25
Total		100

The hopanoid-rich membrane system was constructed through a similar multistep process. Initially, the system was built using the CHARMM-GUI Membrane Builder.^39^ Because hopanoids are not currently present in the CHARMM-GUI lipid library, cholesterol and ergosterol were used as placeholders for the 50 hopanoid molecules required per leaflet. This 1:1 ratio represents the hopanoid enrichment found in *Zymomonas* strains actively producing ethanol,^40^ as hopanoids are essential to microbial fitness under these conditions.^41^ Following initial membrane assembly, each cholesterol or ergosterol molecule was replaced with the specific hopanoids found in *Z. mobilis* membranes.^41^ Finally, as this replacement process can result in ring penetrations, LongBondEliminator was utilized to resolve these artifacts prior to extended simulation.^42^

To study the interactions of small molecules (Figure 1) with the *Z. mobilis* membrane, simulations were performed at varying concentrations of the small molecules (0, 0.5, 1.0, 1.5, 2.0, and 2.5 mol %). Since the carboxylic acids are less hydrophilic than their charged conjugate base, and that the acids are the primary form that crosses a typical lipid bilayer,^43–45^ we only simulate the acid in this study. All small molecules were initially placed in the water phase, as shown in Figure S1. The system was solvated with TIP3P water molecules and was neutralized by adding counterions (Na^+^ or Cl^–^).

### Molecular Dynamics Simulations

All MD simulations were performed using the NAMD 3.0.1 simulation engine.^46^ The lipid parameters were derived from CHARMM36,^47^ while the CGenFF^48^ was employed for the small molecules and hopanoids (bacteriohopanetetrol cyclitol ether and bacteriohopanetetrol glucosamine). This classical molecular simulation captures partitioning, but not reactivity for molecules like aldehydes present in our molecule set.^49^ As is standard for CHARMM36,^47^ we used the TIP3P water model;^50^ the representative initial system setup is shown in Figure S1. Periodic boundary conditions were applied in all directions to avoid edge effects. The system consisted of 100 lipids per leaflet and an initial simulation box of approximately 89 × 93 × 100 Å^3^. The lateral dimensions were chosen to provide sufficient in-plane extent to minimize finite-size effects in heterogeneous membranes, while remaining consistent with established all-atom simulations of multicomponent lipid bilayers.^51–53^

The systems were first energy-minimized using the steepest descent algorithm to remove any steric clashes. The simulations were performed in a constant pressure and temperature (*NpT*) ensemble. The temperature was maintained at 300 K using the Langevin thermostat,^54^ and the pressure was controlled at 1 bar using the Langevin barostat.^55^ The barostat had a piston period of 200 fs and a decay time of 100 fs. Group pressure coupling was used in combination with a flexible cell and a constant cell ratio, as is typical for membrane simulations in NAMD. The flexible cell setting permits the simulation box to adjust in both volume and shape to accommodate anisotropic pressure fluctuations, while the constant ratio between the *x*- and *y*-dimensions keeps the membrane aspect ratio constant. A 2 fs time step was applied throughout the simulation, enabled by constraining bond lengths to hydrogen atoms using SETTLE.^56^ Long-range electrostatic interactions were calculated using the particle mesh Ewald (PME) method^57^ with a grid spacing of 1  Å. van der Waals interactions were truncated at 12  Å with a switching function applied at 10  Å to maintain continuous forces and energies. All of the simulations were performed for three independent runs, each run to 1000 ns. All thermodynamic analysis was carried out for the last 800 ns, considering the first 200  ns as the equilibration time, and statistics were averaged over the three independent runs.

#### Membrane Property Analysis

We measure multiple membrane properties from the simulations described above. All properties were calculated for each of the three replicas, and the results were averaged over all replicas. Error estimates were determined as the standard error of the mean across the average of the individual simulation replicates. All calculations were performed using in-house Python scripts, using numpy,^58^ scikit,^59^ and matplotlib.^60^ The following properties were analyzed to characterize the interactions of small molecules with the *Z. mobilis* membrane.

#### Area Per Lipid

The area per lipid (APL) was determined from the periodic box size of the simulation system, averaged over the equilibrated portion of the trajectory, and divided by the fixed number of lipids in a leaflet. While we could have determined the area per lipid for individual lipids, we focus here on the overall area per lipid to track changes in the membrane structure when the small molecule concentration rises.

#### Membrane Thickness

Similarly, we measured the membrane thickness for the entire membrane by measuring the distance along the membrane normal (*z*) axis between the average position for phosphorus atoms in the upper and lower leaflets.

#### Lipid-Order Parameter (*S*_CH_)

To facilitate comparisons with potential future NMR experiments, we determine the lipid-order parameter, averaged across all lipids along every acyl chain, to measure the overall order as a function of small molecule concentration. The lipid-order parameter, *S*_CH_, is measured in NMR via carbon–deuterium bond orientations, but we are measuring based on the equivalent C–H bond vector. The angle of this bond compared to the membrane normal axis, θ, is used to quantify lipid order

1 $$S_{CH}=\frac{1}{2}\left\langle 3\cos^{2}\theta -1\right\rangle$$

#### Lateral Diffusion Coefficient

Measuring the overall lipid dynamics is done here by measuring the lateral diffusion of individual lipids within the larger molecular system. The lipid lateral diffusion coefficient (*D*_*xy*_) was calculated from the mean square displacement (MSD) of lipids in the *xy*-plane using the relation

2 $$D_{xy}=\frac{\left\langle MSD_{xy}\right\rangle }{4}$$

To compute *D*_*xy*_, the molecular dynamics trajectory was divided into consecutive 10 ns segments. For each segment, the MSD of the lipid phosphorus atoms was calculated relative to the first frame of that segment. A linear regression was then performed on the MSD versus time data for each segment, and the fitted slope was used to determine the *D*_*xy*_ according to eq 2. The final diffusion coefficient was reported as the mean of all segment-based values, with the standard error of the mean calculated to represent variability across segments.

#### Probability Density and Free Energy Profile for Small Molecules across the Membrane

From our extensive sampling, it is possible to measure the probability distributions for the various small molecules across the membrane. This is done by determining the center of mass for each molecule with respect to the membrane normal and then histogramming the resulting positions to determine a probability distribution. The probability distribution can then be converted into a free energy profile by the well-worn relationship

3 $$\Delta G=-RT\;\ln\frac{p}{p_{0}}=G_{p}-G_{p_{0}}$$

in eq 3, *p* is the probability within a specific bin, while *p*_0_ is the probability in a reference bin within the histogram. We think it is most helpful if the small molecule in solution is considered to be the zero point for free energy, which we accomplish by selecting the probability in solution as *p*_0_.

#### Flux-Based Estimation of Permeability

The permeability values were calculated directly from the statistics of complete leaflet-to-leaflet crossings observed in the atomistic MD trajectories. For a given solute, we recorded the total number of full translocation events *N* in three independent 1000 ns replicas of a hydrated *Z. mobilis* model membrane. A permeability coefficient was then computed following the analysis framework from Venable et al.^61^

4 $$Pm=\frac{N}{2A\;\Delta t\;C}$$

where the factor 2 accounts for the two bilayer faces, Δ*t* is the aggregate simulation time and *C* is the instantaneous aqueous concentration extracted from the bulk region, and *A* is the area of the lipid bilayer.

## Results

There are two overall goals for the simulations. First, using equilibrium molecular-dynamics simulations, we quantify how membrane structure and dynamics in *Z. mobilis* respond to biomass-derived small molecules as their alkyl chain length and concentration increase. Second, we explore the role of hopanoids within the membrane and how they contribute to exceptional ethanol tolerance by simulating a hypothetical membrane in which all hopanoids are removed. The results addressing these two objectives are presented sequentially in the following sections.

### Control Membrane Structure and Dynamics

Before probing structure and dynamics changes within the membrane precipitated by small molecule action, it is important to know what occurs when small molecules are absent. Thus, we quantify the intrinsic behavior of the *Z. mobilis* model membrane in the absence of exogenous stressors, when hopanoids are present and absent from the membrane model. The resulting baseline membrane properties in Table S1 and Figure 3 measure the average structure and dynamic properties from the individual time series from Figures S2 and S3.

**Figure 3:**
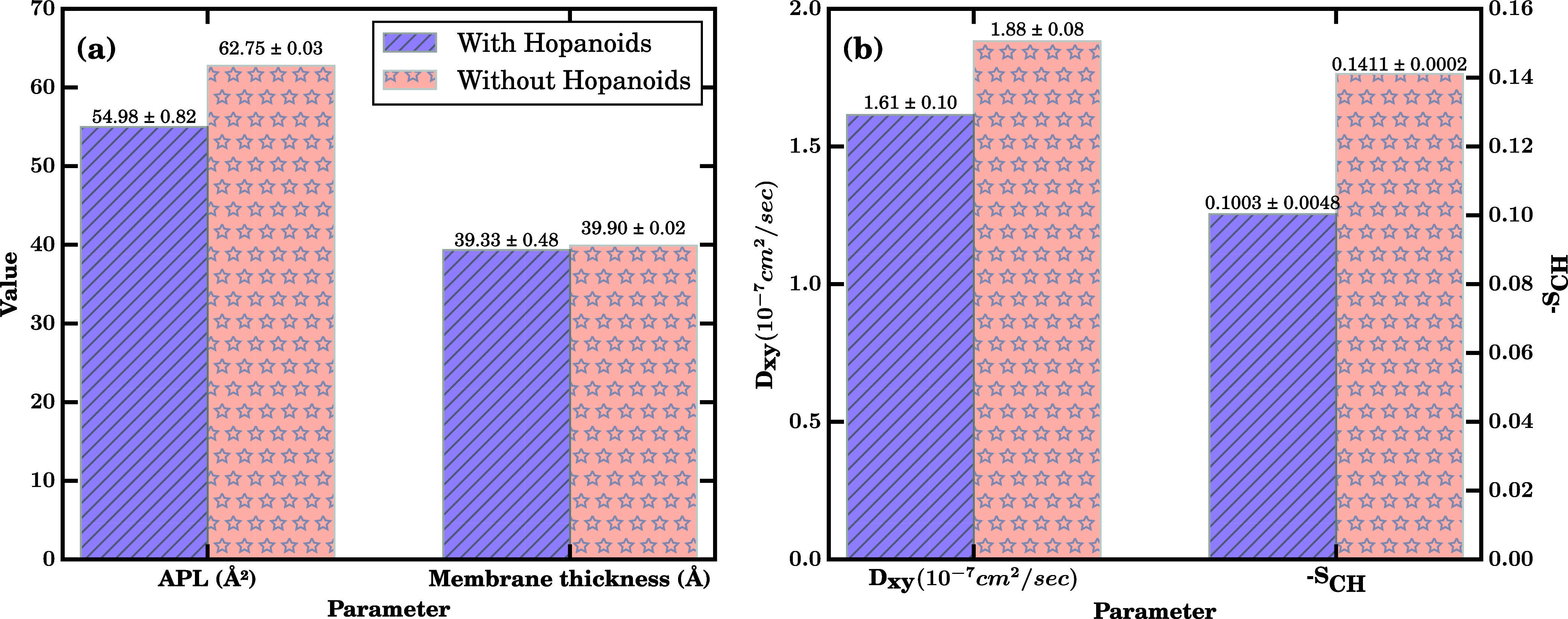
Comparison of control bilayer properties; (a) APL and membrane thickness and (b) *D*_*xy*_ and –*S*_CH_; in the presence (blue) and absence (red) of hopanoids. Simulations were performed in the *NpT* ensemble at 300  K and 1 bar. Each bar represents the mean over the final 800 ns of three independent trajectories, with values being printed above each bar for clarity and also reproduced in Table S1.

Comparing between the standard *Z. mobilis* and the hypothetical hopanoid-free membrane, there are clear differences in membrane structure and dynamics. Removing hopanoids increases the area per lipid by ∼15% and the lipid lateral diffusion coefficient *D*_*xy*_ by ∼19%. In our simulations, eliminating hopanoids spreads apart the lipids laterally yet leaves the hydrophobic core thickness essentially unchanged, indicating that hopanoids function primarily as in-plane condensing agents rather than as modulators of bilayer thickness—a behavior reminiscent of other sterol like lipids.^62^ Pentacyclic hopanoids intercalate among phospholipid acyl chains to promote tighter packing and reduce the free surface area. However, the rigid, nearly cylindrical shape for the ring system present in the hopanoids (Figure 2) means that the tighter packing does not expand the membrane along the membrane normal and keeps the membrane structural orientation intact. One area where this is shown explicitly is in the order parameter analysis, where the removal of the hopanoids leads to ∼40% increase in the –*S*_CH_ value (Figure 3). The membrane with hopanoid composition is more condensed and less fluid. Because membrane permeability generally scales with both area per lipid and lipid mobility, the hopanoid-rich state is expected to confer an inherent barrier to small-molecule entry, whereas the hopanoid-depleted control is primed for higher passive uptake.

### Membrane Dynamics and Structure Shifts under Stress

Having determined the baseline membrane structure and dynamics in a simple aqueous environment, we studied the impact different classes of small molecules have on the structure and dynamics of *Z. mobilis* membranes. In Figure 4, we report the membrane thickness, area per lipid, *D*_*xy*_ (eq 2), and –*S*_CH_ (eq 1), as we increase the solute concentration from 0.5% to 2.5 mol %. Figure 4 makes it clear that none of the five hydrolysate-derived metabolites cause a significant damage to the *Z. mobilis* bilayer, yet a set of small, internally consistent shifts emerges once we examine each observable in turn.

**Figure 4:**
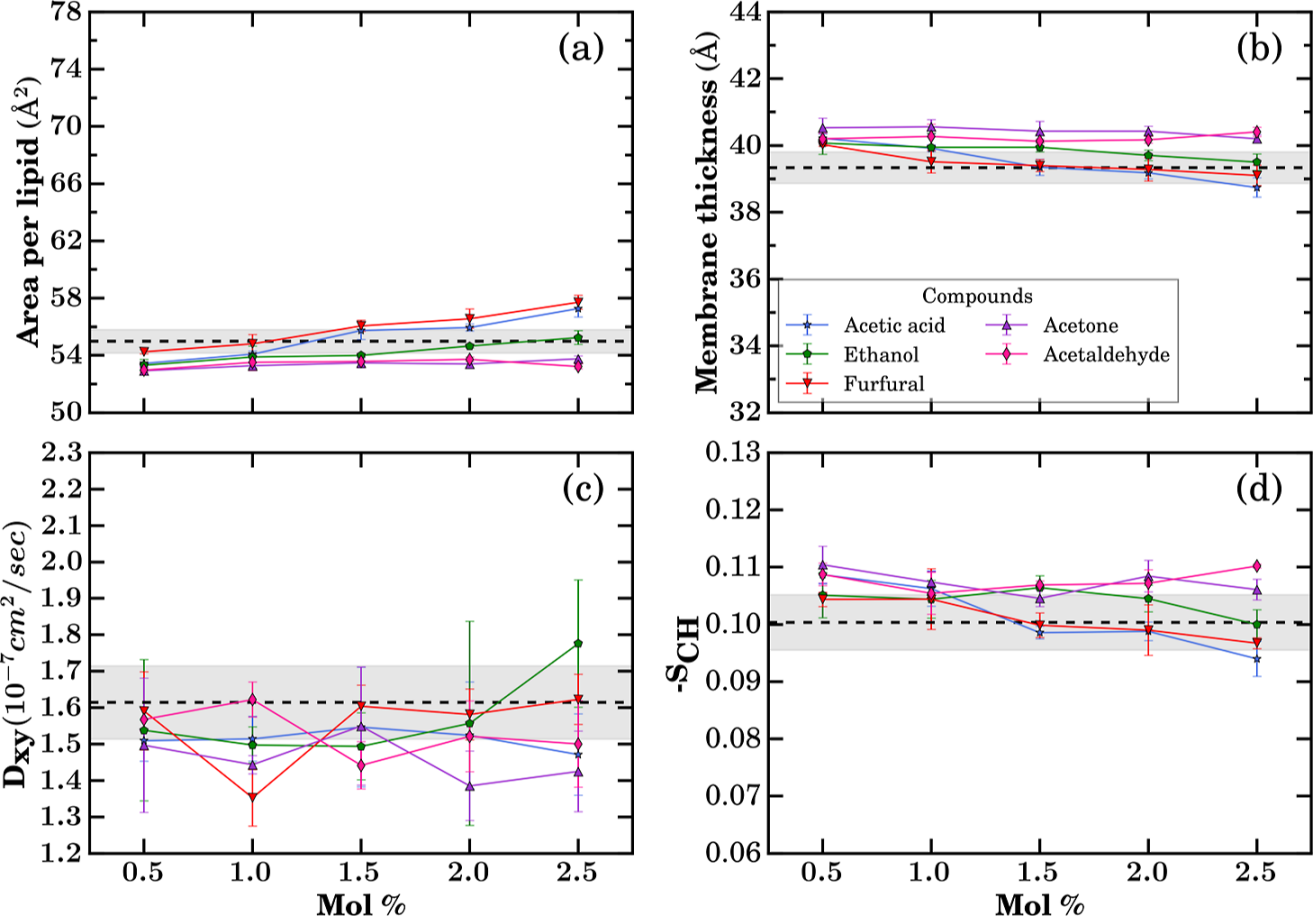
Membrane structure and dynamic properties quantified with increasing concentrations of small molecules from different compound classes introduced in Figure 1: (a) area per lipid, (b) membrane thickness, (c) lateral diffusion coefficient (*D*_*xy*_), and (d) deuterium-order parameter (–*S*_CH_). Simulations were performed in the *NpT* ensemble at 300 K and 1 bar. Each data point represents the mean over the final 800 ns of three independent trajectories. The black dashed line shows the average values, and the gray area shows the standard deviation from control membrane simulation runs when no small molecules were present. The time series data for the individual runs of each molecule is shown in Figures S4–S28.

Among the studied small molecules, furfural, acetic acid, and ethanol produce a measurable, quasi-linear increase in area per lipid with rising mole fraction, with furfural showing the largest perturbation at 2.5 mol %. In contrast, acetone and acetaldehyde do not exhibit statistically significant changes relative to the control, consistent with the confidence intervals shown in Figure 4a. Even for the most perturbing solutes, the changes remain modest. The mean area per lipid stays within the range of area per lipid values sampled in the control simulations without solutes added (gray area in Figure 4), reinforcing that the bilayer remains in the liquid crystalline regime in the studied concentration range.

Membrane thicknesses display a related pattern. All five solutes produce a mild concentration-dependent decrease in phosphate-to-phosphate thickness (Figure 4b), with the control spanning 39.3 ± 0.5 Å. By 2.5 mol %, acetic acid, ethanol, and furfural induced a small but detectable thinning of the membrane.

These structural changes have a far smaller impact on the dynamics. Figure 4c shows the *D*_*xy*_ changing in response to the small molecules. In the solute-free control, *D*_*xy*_ is (1.61 ± 0.10) × 10^–7^ cm^2^ s^–1^. The *D*_*xy*_ value does not show a significant change, and it remains consistent across different small molecules within the tested concentrations.

Orientational order parameters –*S*_CH_ show small but internally consistent changes (Figure 4d). Ethanol induces only minimal tail disorder even at 2.5 mol %, consistent with the high ethanol tolerance observed for *Z. mobilis*. Furfural and acetic acid yield slight decreases in order at higher concentrations, aligning with their observed increases in area per lipid and decreases in membrane thickness (Figure 4a,b).

Overall, the analysis portrays a membrane that is remarkably resilient, even at 2.5 mol %. None of the solutes drive any observable outside the range sampled by the control simulations (gray regions in Figure 4). While the hydrolysate components modulate membrane properties in predictable, dose-dependent manner, the perturbations remain well within the tolerance window of *Z. mobilis* membranes (Figure 3). Still, coherent patterns emerge. First, there is a universal, dose-dependent reduction in membrane thickness and a commensurate APL increase. Second, compounds such as furfural and acetic acid cause the maximum perturbation to the membrane among the studied compounds.

### Effect of Chain Length on Membrane Dynamics

Whereas Figure 4 scans across multiple small molecule chemistries, converting from alcohols to aldehydes to acids, Figure 5 explores what happens as we add hydrophobic bulk to a subset of these molecules. For acids and alcohols, we are adding carbons to the small molecule as we move from formic acid to acetic acid to propanoic acid or from ethanol to propanol to isobutanol. HMF, while larger than furfural, is also less hydrophobic, which ends up having a strong impact on the trends observed in Figure 5.

**Figure 5:**
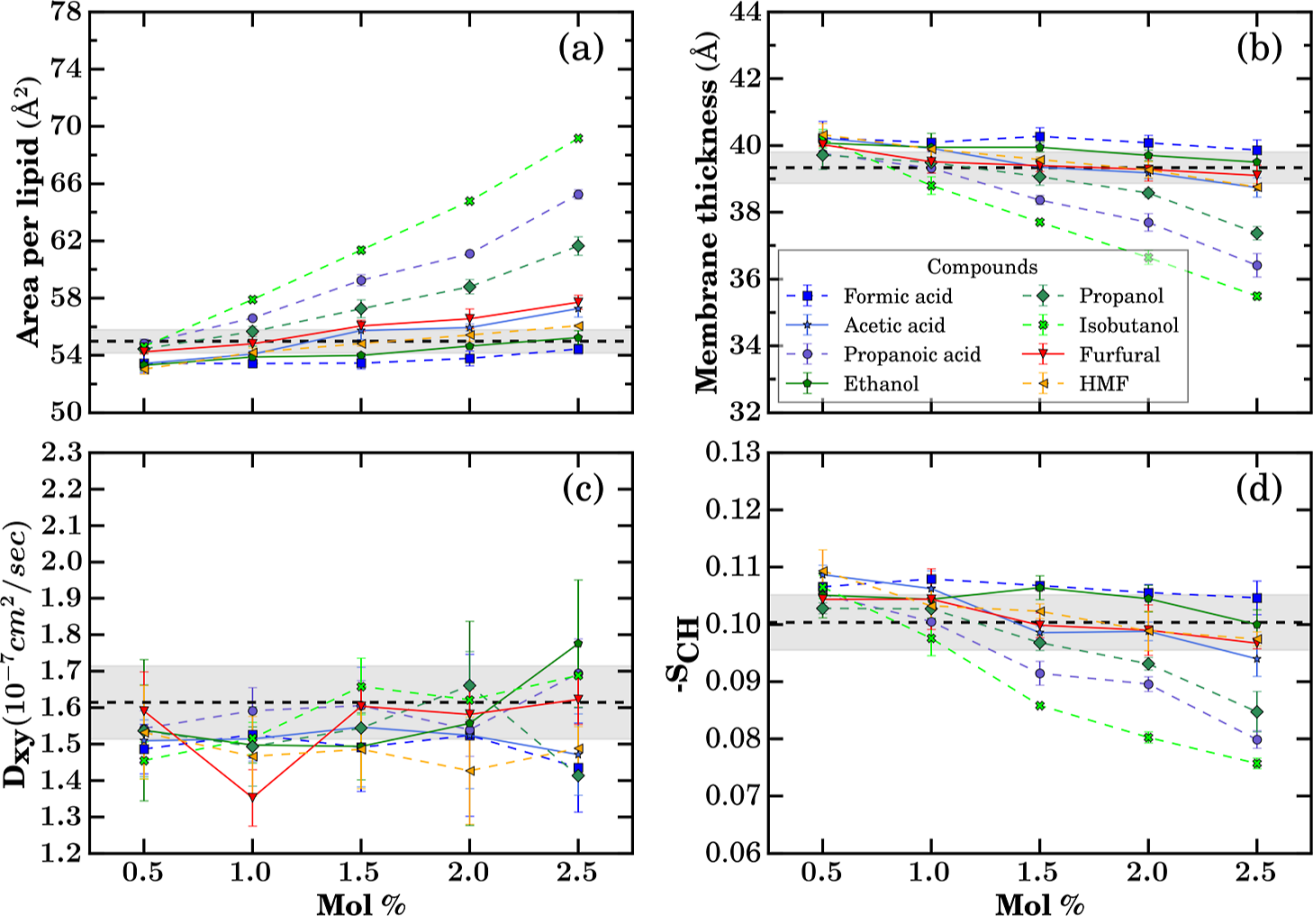
Membrane structure and dynamic properties quantified with increasing alkyl chain length and concentrations of small molecules. (a) Area per lipid, (b) membrane thickness, (c) lateral diffusion coefficient (*D*_*xy*_), and (d) deuterium-order parameter (–*S*_CH_). Simulations were performed in the *NpT* ensemble at 300 K and 1 bar. Each data point represents the mean over the final 800 ns of three independent trajectories. The black dashed line shows the average values, and the gray area shows the standard deviation from control membrane simulation runs when no small molecules were present. The time series data for the individual runs of each molecule is shown in Figures S4–S18 and S29–S48.

Starting from Figure 5a, the maximum area per lipid within the data set is far higher than we observed for the control membrane in Figure 3a. Particularly for the larger, more hydrophobic molecules within the data set (isobutanol, propanoic acid, and propanol), the area per lipid is so far above the range seen without small molecules present; this represents a substantial membrane structure perturbation. The increased area per lipid is driven by these molecules intercalating near the membrane headgroups and disrupting the lipid packing. Conversely, smaller molecules occupy less space near the membrane–water interface and thus have less dramatic changes at a similar concentration. Regardless of the molecular size, the area per lipid increases with increasing molecular concentration. Increasing lipid area with increasing small molecule concentration has been previously observed in prior studies.^63–65^

Membrane thickness is the converse of the area per lipid. Since the size of individual lipids is fixed, a larger spacing between lipids tends to thin membranes. Thus, the hydrophobic molecules that show the greatest increase in area per lipid show the greatest decrease in membrane thickness (Figure 5b). More broadly, increasing concentrations for these molecules (Figure 1) decrease the membrane thickness, although for most compounds the trend is relatively weak within the studied concentration range.

Higher concentrations of small molecules can disrupt lipid–lipid interactions, leading to bilayer destabilization. Given how important hydrophobic matching is to membrane protein function, the reduced thickness for the most hydrophobic compounds is likely to substantially impact membrane protein function^66,67^ and certainly is a plausible mechanism by which these molecules would stress *Z. mobilis*. Despite these structural changes, the *D*_*xy*_ is strikingly resilient: within statistical uncertainty, it remains nearly constant across the full concentration range examined (Figure 5c). This invariance indicates that lipid mobility, hence the viscous properties of the membrane interior, is largely preserved even as the bilayer spreads laterally and becomes thinner.

Returning to structural metrics, the lipid-order parameter –*S*_CH_ quantifies the acyl tail rigidity and orientation. Thinner membranes with larger spaces between lipids introduce additional disorder into the membrane, and so the overall trend in Figure 5d is for decreased order, mimicking the trend for membrane thickness. Just like the other structure measures, the largest changes occur for isobutanol, propanol, and propanoic acid. The observed shift for these molecules of approximately 0.02 is similar to the shift observed for plant membranes when raising the simulated temperature by 50 °C.^68^ Unlike temperature, which changes relatively slowly over the course of a day, concentration changes in a hydrolysate stream can change far more rapidly, emphasizing how challenging such an environment is for microbes in general. These findings demonstrate that even within the same class of molecules, variations in the number of carbon atoms and molecular structure significantly influence the nature and extent of their interaction with the lipid membrane. In both carboxylic acids and alcohols, increasing the alkyl chain length generally leads to a greater perturbation of the membrane in terms of lateral expansion and increased fluidity (disorder of lipid tails) and also tends to slightly decrease in membrane thickness.

To further assess the association of hopanoids and other lipids within the membrane system, we quantified the lateral distribution of hopanoids and other lipids using the lipid enrichment/depletion index implemented in LiPyphilic.^69^ In this analysis, an enrichment index of 1 corresponds to random mixing, values >1 indicate an increased probability of finding a given lipid type as a nearest neighbor, and values <1 indicate depletion. We grouped lipids into four classes: hopanoids (HOP), saturated tails (sat), monounsaturated tails (MU), and polyunsaturated tails (PU), and computed the grouped enrichment index matrices for two representative systems: (i) acetaldehyde at 0.50 mol %, which causes the smallest perturbation in global membrane properties (Figure 4), and (ii) isobutanol at 2.50 mol %, which causes the largest perturbation (Figure 5).

The resulting enrichment matrices (Figures S49 and S50) show that when hopanoids are taken as the reference lipid, all neighbor types (including other hopanoids) have enrichment indices ≈0.9 in both systems, indicating a mild depletion of all lipid types in the immediate vicinity of hopanoids relative to random mixing. For example, a Sat–HOP enrichment value of 1.12 would mean that hopanoids are 12% more likely to appear next to a saturated lipid than expected from the bulk composition, whereas a HOP–HOP value of ≈0.9 would indicate a slight tendency against hopanoids sitting next to each other. When phospholipids (Sat, MU, PU) are used as reference lipids, hopanoids appear only modestly enriched as neighbors (indices typically ∼1.1), and the indices for phospholipid–phospholipid contacts lie close to unity and within a relatively narrow range (roughly 0.8–1.2) for all pairs. We therefore do not observe strong hopanoid–hopanoid self-enrichment or any lipid pair with a pronounced enrichment or depletion that would clearly indicate stable hopanoid-rich or hopanoid-poor lateral domains on the simulated length scale. This behavior is similar for the minimally perturbed acetaldehyde system (Figure S50a) and for the more strongly perturbed isobutanol system (Figure S50b), suggesting that, in these two representative cases, the lipids remain largely laterally mixed.

Collectively, these enrichment maps, combined with the global APL and thickness data (Figures 4 and 5), suggest that hopanoids mainly act as agents that condense the membrane in-plane, influencing its average packing and fluctuations rather than creating distinct thick or thin regions within the simulation boxes and time scales we examined.

### Molecular Distribution and Free Energy Profile across the Membrane

To elucidate the small molecule partitioning behavior within the *Z. mobilis* membrane, we computed the small molecules’ probability density distributions relative to the bilayer center (Figures S51 and S52). From these probabilities, we can further quantify molecular translocation energetics by evaluating the free energy profiles by the Boltzmann inversion of the probability distributions (Figure 6). These distributions provide insight into the preferential localization of each compound and its potential interaction with membrane components, and the free energy profiles provide a measure of the energetic barriers associated with membrane traversal and allow for comparative analysis of molecular permeability.

**Figure 6:**
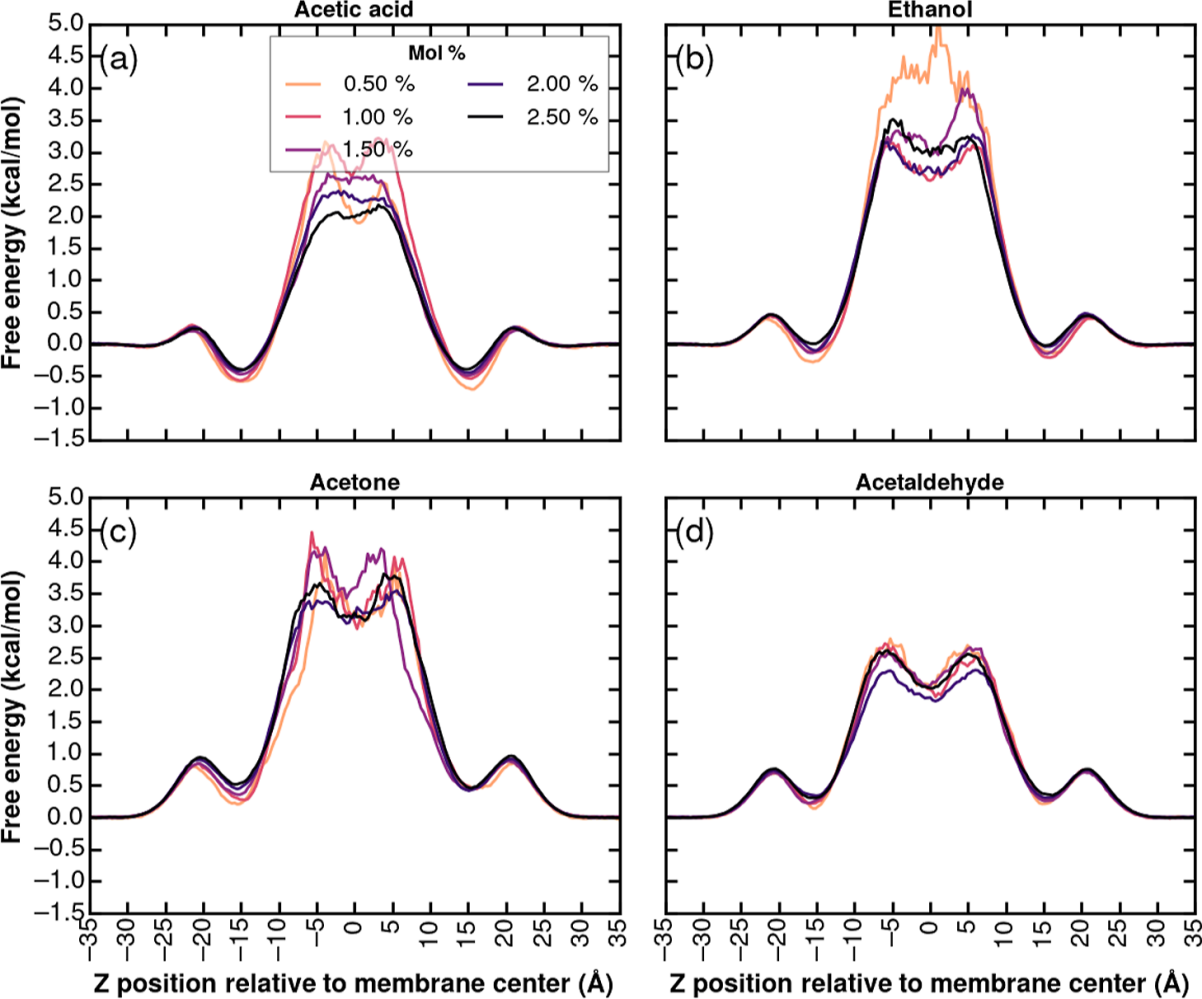
Free energy profiles obtained from the Boltzmann inversion of probability distributions reported in Figure S51 for (a) acetic acid, (b) ethanol, (c) acetone, and (d) acetaldehyde.

We first studied the probability distributions in different classes of molecules, shown in Figure S51. The selected compounds, acetic acid, ethanol, acetone, and acetaldehyde, span a range of chemical classes, enabling a comparative evaluation of how different chemical class compounds influence membrane partitioning and insertion energetics. As shown in Figure S51, trends suggest that most molecules exhibit peak densities at the lipid–water interface and significantly lower probabilities in the hydrophobic core with the degree of central bilayer penetration strongly correlating with hydrophobicity.

Acetic acid and ethanol showed a sharply peaked distribution at the interface and minimal presence in the membrane core. This pattern reflects the compound’s affinity for the polar headgroup region and aversion to the hydrophobic interior, which is natural for these small molecules with substantial hydrophilic groups. With an increasing concentration, the interfacial peaks in Figure S51 increased slightly, suggesting minor penetration into deeper membrane regions at higher loading. The free energy distributions for acetone and acetaldehyde show lower peak values at the membrane–water interface as compared to those at acetic acid and ethanol.

The probability distributions in Figure S51 can be inverted into free energy profiles to express these probabilities for the small molecule solutes in energy terms. The free energy Δ*G*(*z*) as a function of position *z* relative to the bilayer center was calculated by using eq 3. This formulation directly relates the statistical occupancy of solutes at different membrane depths to their underlying thermodynamic stability. The computed free energy profiles (Figure 6) reflect the hydrophobicity trends of these molecules (acetaldehyde < acetone < ethanol < acetic acid). A consistent feature across all molecules is the presence of a free energy minimum at the bilayer interface, approximately 15–20  Å from the membrane center. This minimum corresponds to a region where solutes experience partial insertion into the bilayer, interacting with both hydrophilic headgroups and partially exposed hydrophobic acyl chains. Because the bilayer and solute loading are symmetric about the membrane midplane, the underlying free energy profiles are expected to be symmetric; the mild left–right differences seen in Figure 6 arise from finite sampling rather than any built-in structural asymmetry.

At 0.5 mol %, acetic acid exhibited a well-defined central energy barrier (∼3.5 kcal/mol), which decreased slightly with concentration (Figure 6a). The prominent interfacial minima and significant barrier at the core are consistent with its strong preference for the polar environment and the high energetic cost of insertion into the hydrophobic bilayer center. This pronounced interfacial stabilization in acetic acid arises from its high polarity and capacity for hydrogen bonding. The carboxylic acid group readily forms hydrogen bonds with the phosphate or glycerol moieties of lipid headgroups, promoting a strong interfacial association. Ethanol (Figure 6b), on the other hand, exhibited a central energy barrier of ∼5 kcal/mol at 0.5 mol %, which decreased slightly with concentration.

The profiles of acetone and acetaldehyde (Figure 6c,d) also revealed the presence of an interfacial energy barrier when approaching the bilayer from the aqueous phase. Acetone and acetaldehyde possess polar carbonyl groups but lack hydrogen bond donors, limiting their ability to form stabilizing interactions with the lipid headgroups during initial insertion. This leads to a small but detectable energy barrier at the onset of the membrane interface, representing the disruption of favorable solute–water interactions without fully compensatory interactions with the membrane.

These interfacial minima also evolve with the concentration. For acetic acid and ethanol, increasing the mole percent reduces the interfacial stabilization slightly (Figure 6a,b), likely due to saturation of hydrogen-bonding sites or local reorganization of the lipid environment. This effect is less pronounced in the other compounds, consistent with their weaker interfacial interactions. Overall, these trends illustrate that the depth of the interfacial free energy well is a direct consequence of solute hydrophilicity and its chemical capacity for hydrogen bonding and plays a crucial role in determining membrane permeability and accumulation behavior.

### Effect of Alkyl Chain Length on Membrane Partitioning and Energetics

To probe how incremental increases in hydrophobicity modulate solute–membrane interactions, we next analyzed a homologous series of carboxylic acids (formic, acetic, propanoic) and alcohols (ethanol, propanol, isobutanol) in a concentration range of 0.5–2.5 mol %. The probability density distributions and their Boltzmann-inverted free energy profiles (Figures S52 and 7 respectively) reveal systematic trends in the insertion depth, interfacial stabilization, and central barrier heights as the alkyl chain is lengthened.

**Figure 7:**
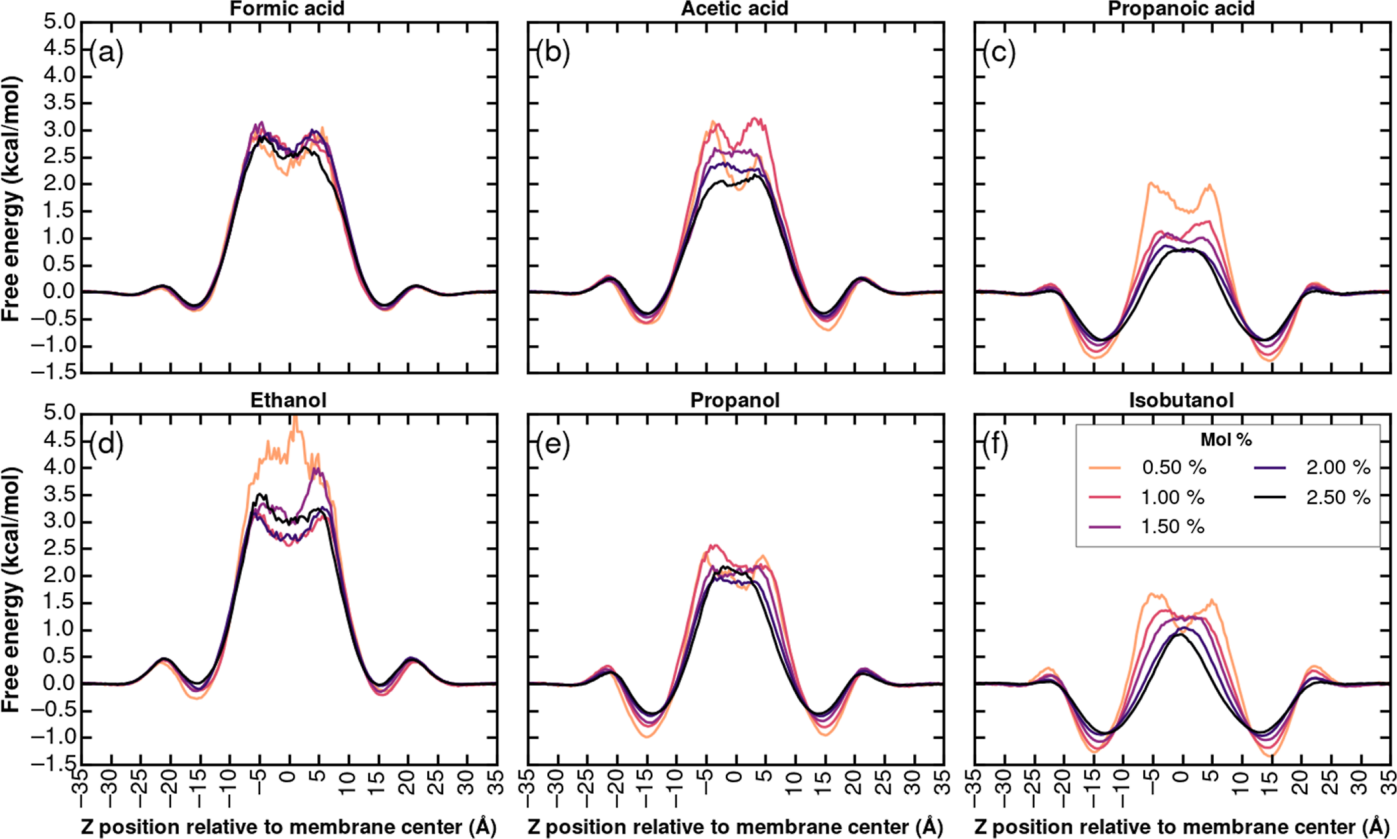
Free energy profiles obtained from the Boltzmann inversion of probability distributions for carboxylic acids (formic, acetic, and propanoic) (a–c) and alcohols (ethanol, propanol, and isobutanol) (d–f).

Formic acid, acetic acid, and propanoic acid remain tightly localized at the lipid–water interface, with a narrow peak at |*z*| ≈ 15  Å. For formic acid, its central free energy barrier is ∼3.5 kcal/mol (Figure 7a). Extending to acetic acid introduces noticeable probability in the upper acyl-chain region (Figure S52), and lowers the core barrier to ∼2.5 kcal/mol at a higher loading of 2.5 mol % (Figure 7b). Propanoic acid amplifies these shifts, and the central barrier falls further to ∼1.5 kcal/mol at a higher loading of 2.5 mol % (Figure 7c). Across all three acids, increasing the concentration mildly broadens the interfacial maximum and slightly depresses the free energy minima, indicative of concentration-driven lipid perturbation but preserving the hydrophobicity-driven partitioning trend.

A parallel progression is seen for the alcohols. Ethanol exhibits an interfacial peak at |*z*| ≈ 15 Å and a core barrier of ∼3.5 kcal/mol at a higher loading of 2.5 mol % (Figure 7d). Propanol’s distribution is broader, with significant density in the upper acyl chains, and its central barrier decreases to ∼2.5 kcal/mol at a higher loading of 2.5 mol % (Figure 7e). Isobutanol, the most hydrophobic, shows pronounced occupancy throughout the bilayer core (Figure S52) and a minimal barrier of ∼1.5 kcal/mol at a higher loading of 2.5 mol % (Figure 7f). As with the carboxylic acids, higher concentrations introduce slight broadening of the probability profiles and a modest flattening of the free energy wells, reflecting local membrane reorganization at elevated solute loading.

The height of the barrier at the membrane–water interface provides insight into the kinetics of membrane association. Molecules encountering a higher energy barrier (Figures 6 and 7) at the water–membrane interface may exhibit slower rates of insertion and lower interfacial occupancy under equilibrium conditions. This partially explains the broader and more dispersed probability density profiles of acetone and acetaldehyde compared to the sharply localized interfacial peak of acetic acid.

In order to address the asymmetry in the free energy distributions of the inhibitors on the two sides of the membrane, we analyzed the free energy profiles separately for each of the six independent leaflets per condition (three independent simulations with two leaflets each). For each leaflet, the profiles were folded about the membrane midplane and expressed as a function of the absolute distance from the membrane center, |*z*|. This procedure removes any imposed left–right labeling while preserving leaflet-to-leaflet variability. We then computed the mean free energy profile and the corresponding standard deviation across all six leaflets. The resulting one-sided free energy profiles are shown in Figures S74 and S82, confirming that any apparent leaflet-level asymmetries are minimal.

With this symmetry established, the homologous-series data underscore a clear structure–function relationship: each additional methylene unit deepens the interfacial free energy by approximately 1.0 kcal/mol and lowers the energetic cost of traversing the hydrophobic core, thereby enhancing overall membrane permeability in direct proportion to molecular lipophilicity.

These findings emphasize that the initial steps of membrane insertion, specifically the transition from bulk water to the lipid interface, are governed not only by hydrophobicity but also by the molecular capacity to engage in specific interactions with membrane headgroups. The results provide quantitative insight into solute–membrane interactions and underscore how hydrophobicity modulates both thermodynamic and spatial properties of small molecule insertion in *Z. mobilis* membranes.

### Membrane-Crossing and Small Molecule Permeabilities

The event-based analysis of the permeability values reveals a spread of more than 2 orders of magnitude in passive permeability that can be traced to subtle variations in chain length, branching, and aromaticity. The insights for the permeability calculations also correlate with the membrane dynamics (Figures 4 and 5), as greater membrane perturbation caused significant change in the permeability values. Permeability values (Figure S73 and Table S3) were calculated by quantifying the number of permeants that traverse the bilayer (Figure 8 and Table S2). Formic, acetic, and propanoic acids all display pronounced concentration dependence. For formic acid, crossing events increased from 8 at 0.5 mol % to 96 at 2.5 mol %, with log_10_ *Pm* increasing from −1.83 to −1.50 (Table S3) as the number of crossings increased approximately 2× over what would be expected just based on the concentration increase alone. Acetic acid followed the same trend but with greater scatter, suggesting intermittent trapping by hydrogen-bonded clusters at the interface. Propanoic acid benefited most from the additional methylene group, reaching log_10_ *Pm* = −0.96 at 2.5 mol %—around 10-fold gain over formic acid at identical loading (Figure S73).

**Figure 8:**
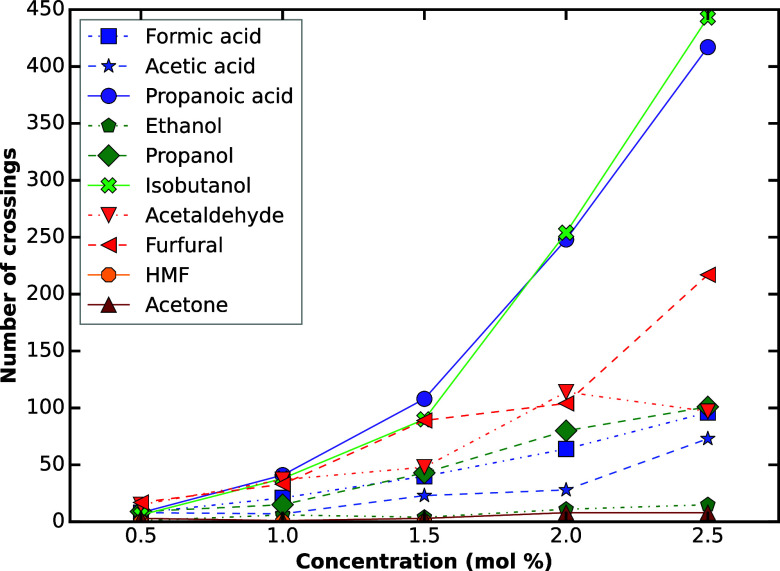
Total crossings for each solute at the range for concentrations from 0.5 mol % to 2.5 mol %, obtained by combining three independent 1000 ns MD trajectories. Data for individual runs is provided in Table S2.

Ethanol, propanol, and isobutanol exhibited steeper gains per mole percent added than the acids, reflecting their weaker interfacial hydrogen bonding than the carboxylic acid compounds. Ethanol remained the least permeable alcohol (mean log_10_ *Pm* = −2.46) because its small nonpolar patch is outweighed by the desolvation penalty of the hydroxyl group. Linear propanol improved to −1.49 at 2.5 mol %, whereas branched isobutanol underwent 443 crossings in the same window, yielding log_10_ *Pm* = −0.98. Branching evidently disrupts lipid packing more effectively (Figure 5), lowering the activation barrier (Figure 7) for spontaneous pore-like defects through which multiple molecules opportunistically slip. These results directly expose the substantial kinetic hurdle caused by the molecule having to lose its water shell and pushing methyl groups through tightly packed acyl chains.

Acetaldehyde displayed intermediate permeability, climbing from −1.57 at 0.5 mol % to −1.33 at 2.0 mol % (Table S3) across the loading series. Acetone, however, remained transport-limited; fewer than 10 full crossings were recorded in any replica, capping log_10_ *Pm* at −2.28 (Table S3).

The permeabilities of furfural and HMF diverged significantly. Furfural’s behavior was comparable to unbranched C_3_ alcohols; its five-membered ring partitioned readily into the glycerol backbone region, and its log_10_ *P*_*m*_ increased from −1.57 to −1.20 with concentration. HMF, in contrast, did not register a single translocation event in any of the 1000 ns MD simulations. We attribute this complete impermeability to persistent hydrogen bonds between HMF’s hydroxymethyl group and interfacial water molecules, which stabilize surface-bound states and effectively suppress excursions into the bilayer core.

In summary, the permeability ranking, averaged across concentrations, is propanoic acid ≈ isobutanol > furfural > propanol > acetaldehyde > formic acid ≈ acetic acid > ethanol > acetone > HMF. The trend shows that permeability generally increases with hydrophobicity, as reflected in the higher transport of propanoic acid and isobutanol compared to smaller or more polar molecules. While it is unsurprising that lipid bilayers are more permeable to hydrophobic compounds, this has downstream implications for biomaterial and biofuel target selection within a biorefinery, as increasing hydrophobicity may make the molecule a larger membrane disruptor.

### Effect of Hopanoids on Solvent-Stressed Membrane Properties

We further extended our study to investigate the stress response of the hypothetical membrane in the absence of hopanoids, for which we calculated the parameters in Figure 3. Figure 9 shows the concentration-dependent response of bilayers that either lack hopanoids (solid traces) or retain 50% hopanoid present in a typical *Z. mobilis* membrane (dashed traces; lipid composition of the *Z. mobilis* model membrane is provided in Table 1). Removing hopanoids roughly doubles the slope, i.e., doubles the sensitivity of the bilayer to a given dose of solvent molecules. This clearly demonstrates the value in using hopanoids to stiffen the membrane to mitigate solvent stress, highlighting why *Z. mobilis* can be such a good host for some bioproducts.

**Figure 9:**
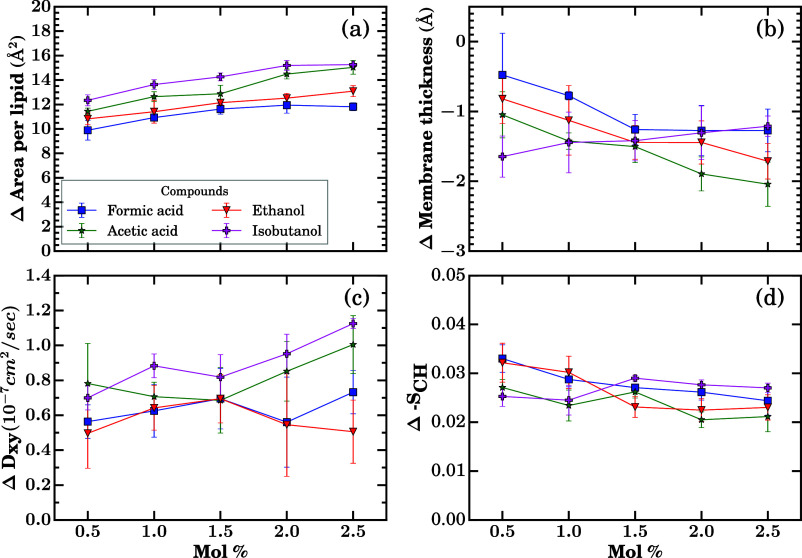
Differences in membrane properties between bilayers without hopanoids and with hopanoids as a function of inhibitor concentration: (a) Δarea per lipid, (b) Δmembrane thickness, (c) Δ*D*_*xy*_, and (d) Δ­(–*S*_CH_). For each quantity, Δ*X* = *X*_without hopanoids_ – *X*_with hopanoids_, so positive values indicate that the property is larger in the absence of hopanoids. Data are shown for formic acid, acetic acid, ethanol, and isobutanol over the 0.5–2.5 mol % inhibitor concentration range. Simulations were performed in the NpT ensemble at 300 K and 1 bar. Each data point represents the difference of mean values for the final 800 ns of three independent MD runs. These differences quantify the hopanoid-induced modulation of bilayer structure, dynamics, and ordering. Exact numerical values for the underlying membrane properties are given in Tables S4–S7.

In hopanoid-free membranes, the area per lipid (Figure 9a) grows in proportion to both inhibitor hydrophobicity and concentration. Formic acid broadens the bilayer by only ∼3 Å^2^ (≈4.5%) across the full concentration series, whereas acetic acid and ethanol produce intermediate expansions of 5–7 Å^2^ (≈10%). Isobutanol is by far the most disruptive: area per lipid rises from ≈67 to ≈85 Å^2^, a ∼27 Å^2^ (≈35%) increase. Introducing hopanoids uniformly shifts the area per lipid curves downward by ∼10 Å^2^, consistent with tighter lateral packing. Isobutanol expands the bilayer in the hopanoid-rich membrane, but only by ∼15 Å^2^ (≈21%). The less hydrophobic inhibitors cause a lesser change to the area per lipid: the net area per lipid change falls to ≤4 Å^2^ for acetic acid and ≤2 Å^2^ for ethanol and formic acid (Table S4).

Membrane thickness (Figure 9b) responds inversely to the area per lipid changes. Without hopanoids, on increasing the inhibitor concentration, bilayer contraction is observed (Figures 4 and 5). On removal of hopanoids, the change in membrane thickness was small for formic acid, acetic and ethanol, while isobutanol drives a pronounced ∼4 Å thinning. The similarity in the membrane thickness values of hopanoid-rich and hopanoid-less membranes demonstrates that hopanoids cannot fully resist the deepest-penetrating larger alkyl-chain-branched alcohol (Table S5).

Changes in the lateral diffusion coefficient *D*_*xy*_ (Figure 9c) mirror the structural response. The hopanoid-free bilayer starts at ∼2.0 × 10^–7^cm^2^ s^–1^ and accelerates modestly (≲ 10%) in the presence of formic acid, ethanol, and acetic acid but by ∼25% in the isobutanol series. Hopanoids set a lower baseline mobility (∼1.5 × 10^–7^cm^2^ s^–1^) and restrict its growth to ≲10% for formic acid and acetic acid inhibitors, but ethanol and isobutanol still reach ≈1.8 × 10^–7^cm^2^ s^–1^ (Table S6).

The deuterium-order parameter *S*_CH_ (Figure 9d) begins higher in hopanoid-free membranes (∼0.14 for formic acid) than in hopanoid-containing ones (∼0.11), indicating that native hopanoids loosen, rather than tighten, the chain backbone at baseline. Nevertheless, hopanoids stabilize the order against further solvent perturbation. Across the 0.5–2.5 mol % range, *S*_CH_ falls by ≲ 0.002 for formic acid, ∼0.0051 for ethanol, ∼0.0147 for acetic acid, and ∼0.031 for isobutanol. The corresponding drops in hopanoid-free membranes are ∼0.010, ∼0.014, ∼0.021, and ∼0.035, respectively. Hence, hopanoids curtail the rate of disorder accumulation, even though their presence confers a slightly more fluid ground state (Table S7).

Taken together, the updated metrics confirm that hopanoids endow the *Z. mobilis* membrane with a quantitative, though not absolute, resilience to lignocellulosic inhibitors. They (i) precompress the bilayer laterally, (ii) raise the hydrophobic-core thickness, (iii) lower the intrinsic lateral diffusivity, and (iv) slow the accrual of acyl-chain disorder. The comparable ∼2-fold slope enhancement seen for the studied inhibitors suggests that hopanoids counter the generic physical effects of small molecules, independent of the chemical functional group, by reinforcing lateral packing and vertical cohesion. This buffering is especially valuable at low inhibitor loadings, where small absolute concentration changes would otherwise produce disproportionately large biophysical responses (Figure 9). The magnitude of protection diminishes as the solute’s hydrophobicity increase, with isobutanol remaining the most challenging molecule even in a hopanoid-rich lipid membrane system.

## Discussion

For lignocellulosic biorefineries to be cost-effective, we need strong, reliable microbes that can survive the tough chemical conditions in biomass hydrolysates. While *Z. mobilis* is a promising platform for biofuel production due to its high ethanol productivity and tolerance, its industrial application is often limited by inhibitory compounds. Several studies have investigated *Z. mobilis* responses to stressors such as ethanol, organic acids, and oxygen.^70–72^ Recent efforts have shifted toward engineering *Z. mobilis* for isobutanol production, yet the microbe’s growth is rapidly arrested by isobutanol at concentrations far below those tolerated for ethanol.^31,32^ This pronounced sensitivity now represents the main bottleneck to achieving the titers and yields required for commercial-scale isobutanolfermentation in engineered strains.

Batch cultures show that growth rates slow once external isobutanol reaches 8–12 g L^–1^ and are virtually abolished at 16 g L^–1^.^32^ Multiomics profiling links this stress to marked membrane swelling, global lipid remodeling, and the appearance of intracellular GFP aggregates at only 0.10–0.15 M.^31^ Our atomistic simulations provide the missing mechanistic link: the branched C_4_ alcohol diminishes the hydrophobic barrier that is able to repel shorter alcohols, enabling deep-core partitioning that expands the area per lipid, lowers acyl-chain order, and thins the hopanoid-rich bilayer. This pronounced structural disruption provides a direct physical explanation for the membrane leakage and protein aggregation phenotypes observed experimentally,^73–76^ highlighting the need for targeted membrane engineering to overcome this challenge.

A key principle for engineering such resilience lies in leveraging the membrane’s native protective components. Our simulations reveal that hopanoids, which are prevalent in the *Z. mobilis* membrane, play a vital, sterol-like condensing role that directly counteracts inhibitor-induced damage. Replacing 50 mol % of hopanoids with phospholipids in silico increased lateral diffusion by 19% and area per lipid by 15%, confirming their sterol-like condensing role. This pre-existing rigidity partially offsets the fluidization imposed by inhibitors and rationalizes why hopanoid-deficient mutants are hypersensitive to stress in vivo.^77,78^ Because hopanoid abundance in *Z. mobilis* can vary with growth conditions and ethanol exposure,^79^ the hopanoid-containing membrane modeled here should be interpreted as a representative of a hopanoid-rich state relevant to ethanol-stressed cells rather than a universal baseline composition. Intriguingly, Rivera-Vázquez et al.^31^ reported that *Z. mobilis* boosts cyclopropane fatty acid (CFA) content under ethanol but fails to do so under isobutanol, despite upregulating CFA synthase.^31^ Our data illuminate the biophysical stakes: CFA rings would counteract the expansion driven by isobutanol, but their synthesis appears blocked, possibly because isobutanol denatures CFA synthase itself, an idea consistent with our observation that isobutanol partitions near the glycerol backbone, where the enzyme acts. Engineering routes that enforce CFA (or hopanoid) overproduction may therefore restore bilayer tightness and raise the lethal isobutanol threshold.^80,81^

Extending this molecular lens to the broader inhibitor palette clarifies empirical trends cataloged in hydrolysate-tolerance reviews.^82^ Yang et al. summarized early high-throughput screens showing that inhibitory strength tracks with compound hydrophobicity across acids, aldehydes, and furans and that combined inhibitors behave additive rather than synergistic effects.^82^ Our systematic homologous-series data quantify that trend: each additional methylene lowers the core free energy barrier for both carboxylic acids and alcohols while simultaneously widening the interfacial free energy well. Weak acids such as acetic acid remain confined to the headgroup region and face barriers to the bilayer core, so their principal effect is electrostatic crowding rather than deep structural disruption. Adding methylene units or reducing the polarity diminishes that barrier and widens the interfacial well, producing a permeability hierarchy that mirrors hydrophobicity-based toxicity rankings from fermenter screens. These atomistic insights convert qualitative stress phenotypes into quantitative design rules, highlighting levers such as hopanoid-pathway optimization, stabilization of CFA synthase, and interface-targeted efflux pumps, which can now be integrated into rational strain-engineering and process-design strategies to create genuinely inhibitor-resistant *Z. mobilis* and robust microbial platforms essential for a circular bioeconomy.

## Conclusions

The MD simulations presented in this study provide a detailed characterization of the interactions between small molecules and the *Z. mobilis* membrane. The increased disorder and thinning at higher concentrations suggest potential compromises in membrane integrity, impacting cell viability in the biorefinery to make fuels or materials. The probability density distributions reveal distinct localization patterns with hydrophilic molecules preferentially residing at the membrane interface and hydrophobic molecules displaying greater penetration into the bilayer core. This suggests that there are fundamental limits to the concentration of organic molecules in solution before the membrane becomes leaky.

The free energy profiles further quantify these trends. We observed that increasing the alkyl-chain length reduces the energetic barrier to cross the lipid membrane, demonstrating that permeability through *Z. mobilis* membranes is primarily governed by solute hydrophobicity, as it is in other systems. These findings have significant implications for biofuel production, microbial tolerance to fermentation inhibitors, and membrane adaptation strategies. Understanding the molecular determinants of permeability in *Z. mobilis* provides a foundation for future efforts in strain engineering, aiming to enhance robustness and optimize yield in industrial fermentation processes.

Given these insights, future efforts to improve stress tolerance in *Z. mobilis* may employ several adaptive strategies to mitigate the impact of toxic compounds on membrane integrity. These could include modifications to lipid composition to alter bilayer permeability, upregulation of efflux transporters to expel toxic metabolites, and adjustments in membrane properties to reduce interactions with inhibitory compounds. Such adaptations could be leveraged in metabolic engineering efforts to enhance microbial robustness in the biorefinery context to enable robust biofuel and bioproduct formation.

## Supplementary Material



## Data Availability

The reduced directory structure that includes analysis scripts, inputs and selected raw outputs used for this publication is available at http://doi.org/10.5281/zenodo.16375964.

## Notes

Representative simulation setup of the *Z. mobilis* membrane system; the time series data for the control membrane in the presence and absence of hopanoids (Table S1, Figures S2 and S3); the time series data for the interaction of small molecules with the hopanoid-rich membrane (Figures S4–S48); grouped lipid enrichment indices for hopanoids (HOP) and phospholipid tail classes in *Z. mobilis* membranes; probability density distributions (Figures S51 and S52); effect of small molecules on the membrane properties when no hopanoids were present in the membrane (Figures S53–S72); permeability values of different compounds at five different concentration ranges (0.5–2.5 mol %) calculated by counting the complete leaflet-to-leaflet crossings (Figure S73); number of membrane-crossing events and calculated permeability coefficients (log  P, cm s^–1^) for different small molecules at five bulk mole fractions; translocation events are counted over a period of 1000 ns for three independent runs (Table S3); and area per lipid, membrane thickness, diffusion coefficient, and *S*_CH_ at two inhibitor concentrations with and without the hopanoid in membrane (Tables S4–S7) (PDF)
