# Supplementary material for: Quantifying Membrane Structure and Dynamics during Bioproduct Production in Zymomonas mobilis by Molecular Simulation
Source: J Phys Chem B. 2026 Feb 18;130(9):2539–53. doi: 10.1021/acs.jpcb.5c06231 (PMC12969272; doi:10.1021/acs.jpcb.5c06231)
Supplement: Supplementary file 1 [file jp5c06231_si_001.pdf]

Quantifying Membrane Structure and Dynamics During Bioproduct Production in *Zymomonas mobilis* by Molecular Simulation

Nitin Kumar Singh[1,2] and Josh V. Vermaas*[1,2,3]

[1]MSU-DOE Plant Research Laboratory, Michigan State University, 612 Wilson Road, East Lansing, MI 48824 USA
[2]DOE Great Lakes Bioenergy Research Center, Michigan State University, 612 Wilson Road, East Lansing, MI 48824 USA
[3]Deparment of Biochemistry and Molecular Biology, Michigan State University, 612 Wilson Road, East Lansing, MI 48824 USA

*Email: vermaasj@msu.edu

Control Membrane Structure and Dynamics

Table S1: Baseline membrane properties with and without hopanoids.

**Parameter**	**With hopanoids**	**Without hopanoids**	Δ (%)
Membrane thickness (Å)	39.33 ± 0.48	39.90 ± 0.02	+0.8
Area per lipid (Å^2)	54.98 ± 0.82	62.75 ± 0.03	+14.8
D_{xy} ($10^{-7}\,\mathrm{cm^2\,s^{-1}}$)	1.61 ± 0.10	1.88 ± 0.08	+18.6
$-S_{\mathrm{CH}}$	0.1003 ± 0.0048	0.1411 ± 0.0002	+40.0

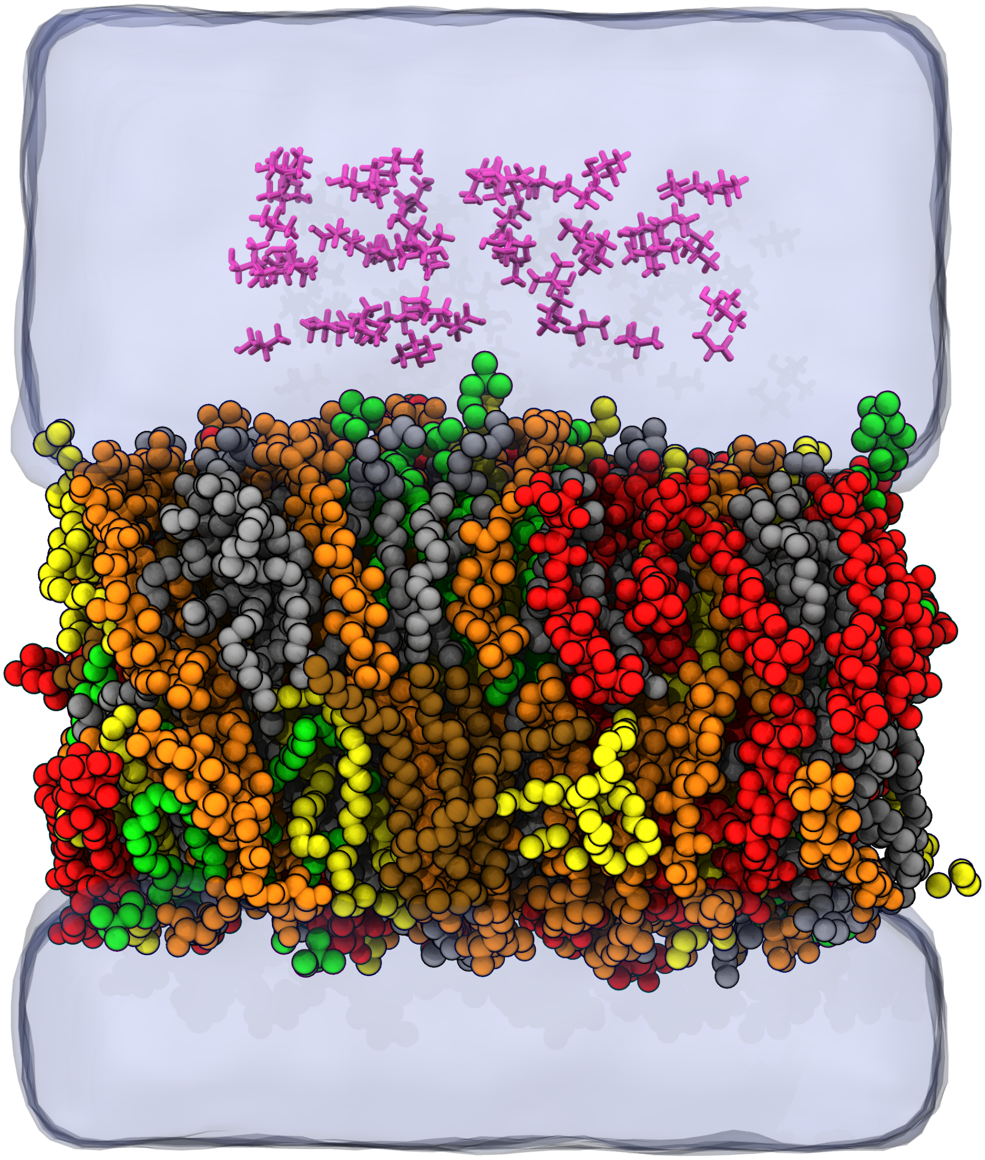

Figure S1: **Representative simulation setup of the *Z. mobilis* membrane system.** The lipid bilayer is depicted in van der Waals representation (multicolored spheres), solvated by water (transparent surface). Bioproduct molecules (magenta sticks) are shown in the aqueous phase prior to equilibration.

Molecular-Dynamics Simulations of Control Membranes With and Without Hopanoids

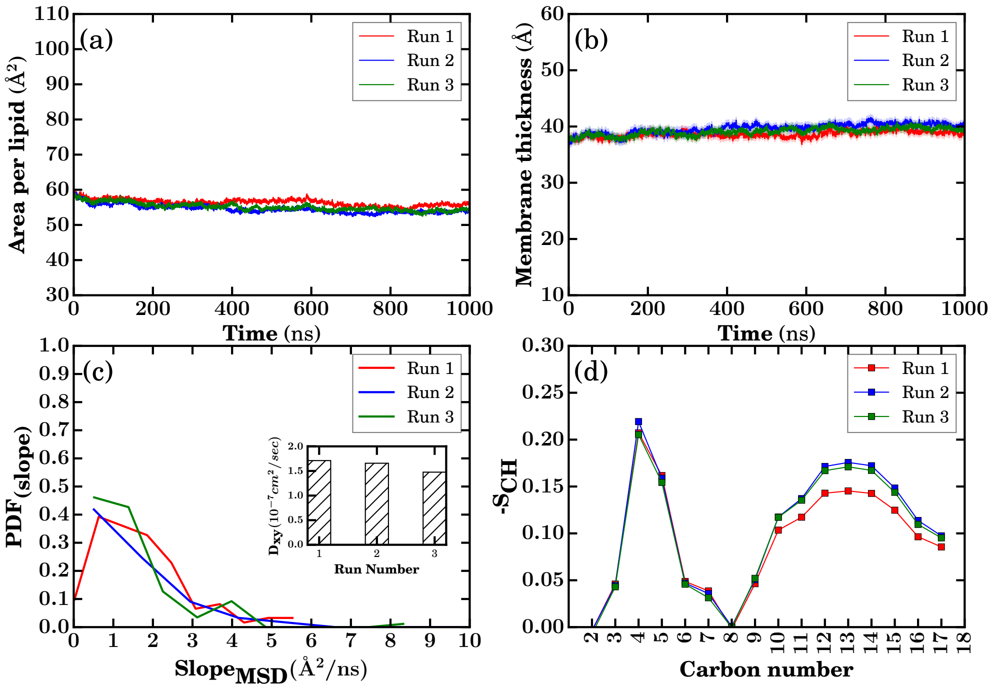

Figure S2: The *Z. mobilis* membrane properties in the presence of Hopanoids in the lipid membrane (a) Area per lipid, (b) Membrane thickness, (c) Distribution of MSD slopes calculated at 10 ns chunks from the MD trajectory with the inset showing the lateral diffusion coefficient (D_{xy}), (d) Deuterium order parameter ($-S_{\mathrm{CH}}$), in an NpT ensemble at 300 K and 1 bar.

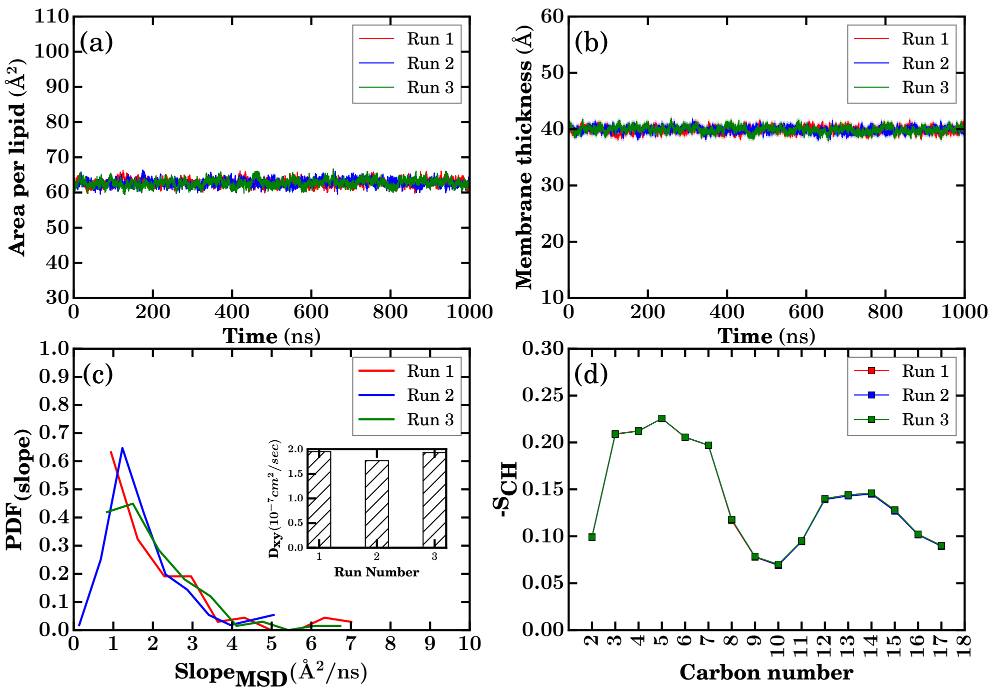

Figure S3: The *Z. mobilis* membrane properties in the absence of Hopanoids in the lipid membrane (a) Area per lipid, (b) Membrane thickness, (c) Distribution of MSD slopes calculated at 10 ns chunks from the MD trajectory with the inset showing the lateral diffusion coefficient (D_{xy}), (d) Deuterium order parameter ($-S_{\mathrm{CH}}$), in an NpT ensemble at 300 K and 1 bar.

Effect of Different Classes of Molecules on Membrane Dynamics

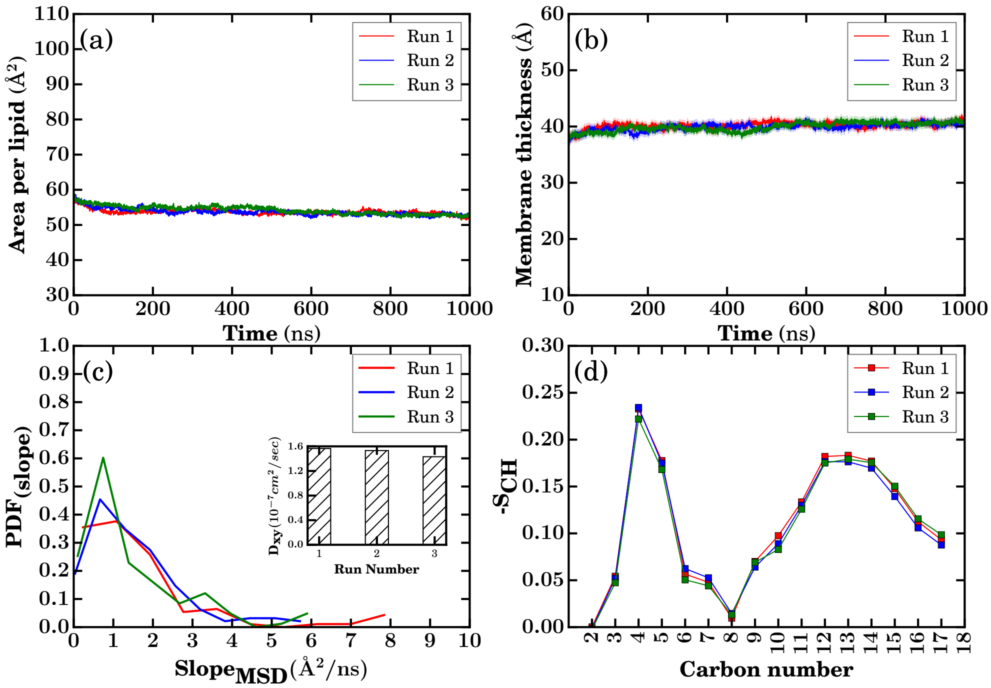

Figure S4: Effect of 0.50 mol% Acetic acid on membrane properties (a) Area per lipid, (b) Membrane thickness, (c) Distribution of MSD slopes calculated at 10 ns chunks from the MD trajectory with the inset showing the lateral diffusion coefficient (D_{xy}), (d) Deuterium order parameter ($-S_{\text{CH}}$), in an NpT ensemble at 300 K and 1 bar.

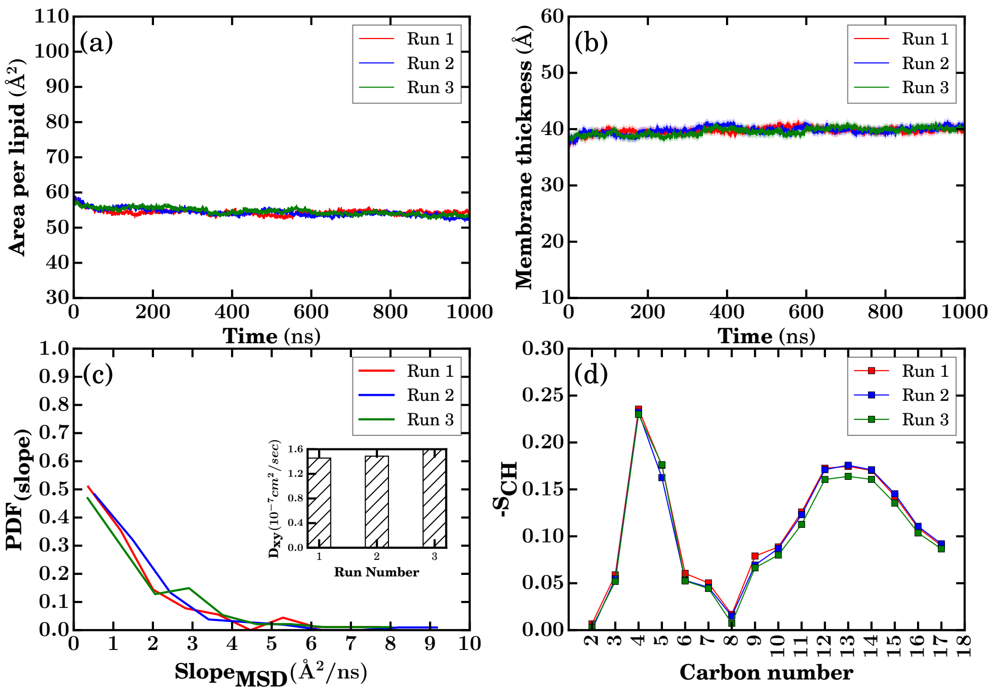

Figure S5: Effect of 1.00 mol% Acetic acid on membrane properties (a) Area per lipid, (b) Membrane thickness, (c) Distribution of MSD slopes calculated at 10 ns chunks from the MD trajectory with the inset showing the lateral diffusion coefficient (D_{xy}), (d) Deuterium order parameter ($-S_{\mathrm{CH}}$), in an NpT ensemble at 300 K and 1 bar.

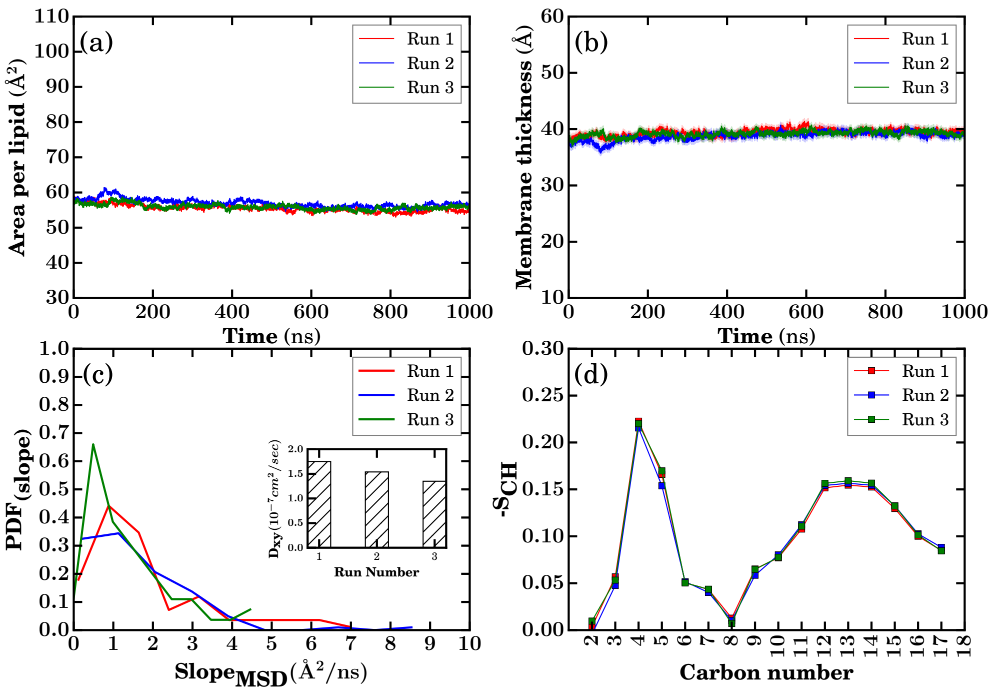

Figure S6: Effect of 1.50 mol% Acetic acid on membrane properties (a) Area per lipid, (b) Membrane thickness, (c) Distribution of MSD slopes calculated at 10 ns chunks from the MD trajectory with the inset showing the lateral diffusion coefficient (D_{xy}), (d) Deuterium order parameter ($-S_{\mathrm{CH}}$), in an NpT ensemble at 300 K and 1 bar.

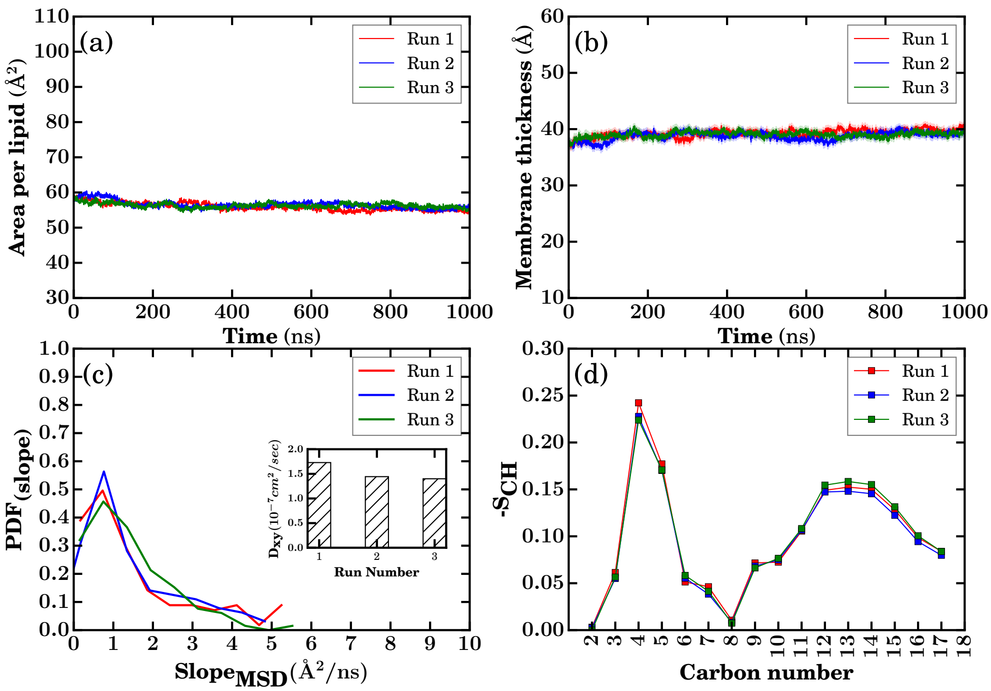

Figure S7: Effect of 2.00 mol% Acetic acid on membrane properties (a) Area per lipid, (b) Membrane thickness, (c) Distribution of MSD slopes calculated at 10 ns chunks from the MD trajectory with the inset showing the lateral diffusion coefficient (D_{xy}), (d) Deuterium order parameter ($-S_{\mathrm{CH}}$), in an NpT ensemble at 300 K and 1 bar.

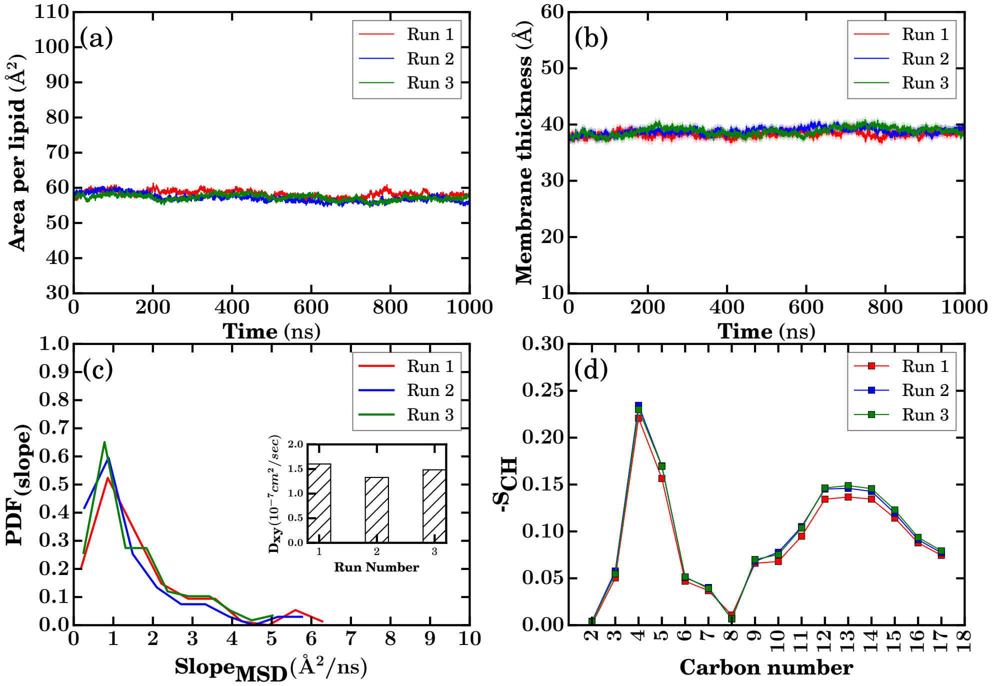

Figure S8: Effect of 2.50 mol% Acetic acid on membrane properties (a) Area per lipid, (b) Membrane thickness, (c) Distribution of MSD slopes calculated at 10 ns chunks from the MD trajectory with the inset showing the lateral diffusion coefficient (D_{xy}), (d) Deuterium order parameter ($-S_{\mathrm{CH}}$), in an NpT ensemble at 300 K and 1 bar.

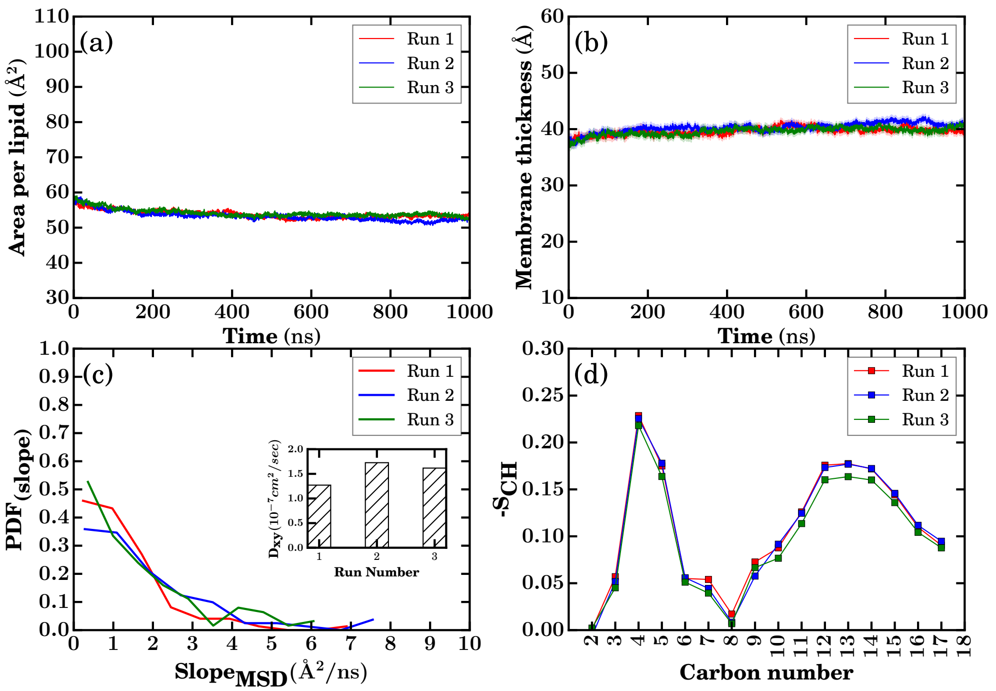

Figure S9: Effect of 0.50 mol% Ethanol on membrane properties (a) Area per lipid, (b) Membrane thickness, (c) Distribution of MSD slopes calculated at 10 ns chunks from the MD trajectory with the inset showing the lateral diffusion coefficient (D_{xy}), (d) Deuterium order parameter ($-S_{\text{CH}}$), in an NpT ensemble at 300 K and 1 bar.

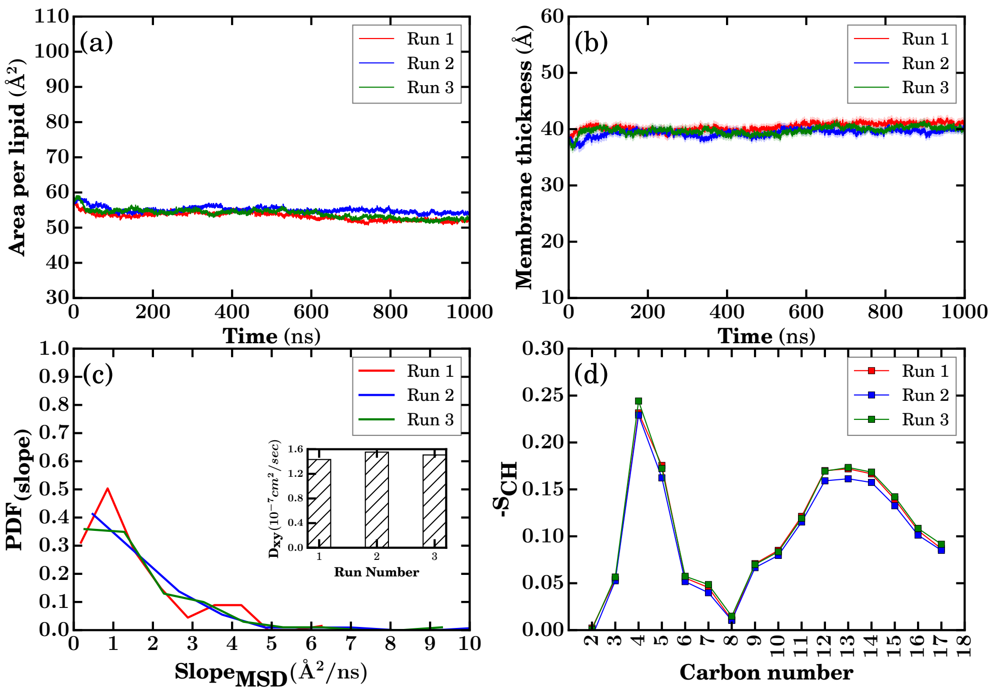

Figure S10: Effect of 1.00 mol% Ethanol on membrane properties (a) Area per lipid, (b) Membrane thickness, (c) Distribution of MSD slopes calculated at 10 ns chunks from the MD trajectory with the inset showing the lateral diffusion coefficient (D_{xy}), (d) Deuterium order parameter ($-S_{\mathrm{CH}}$), in an NpT ensemble at 300 K and 1 bar.

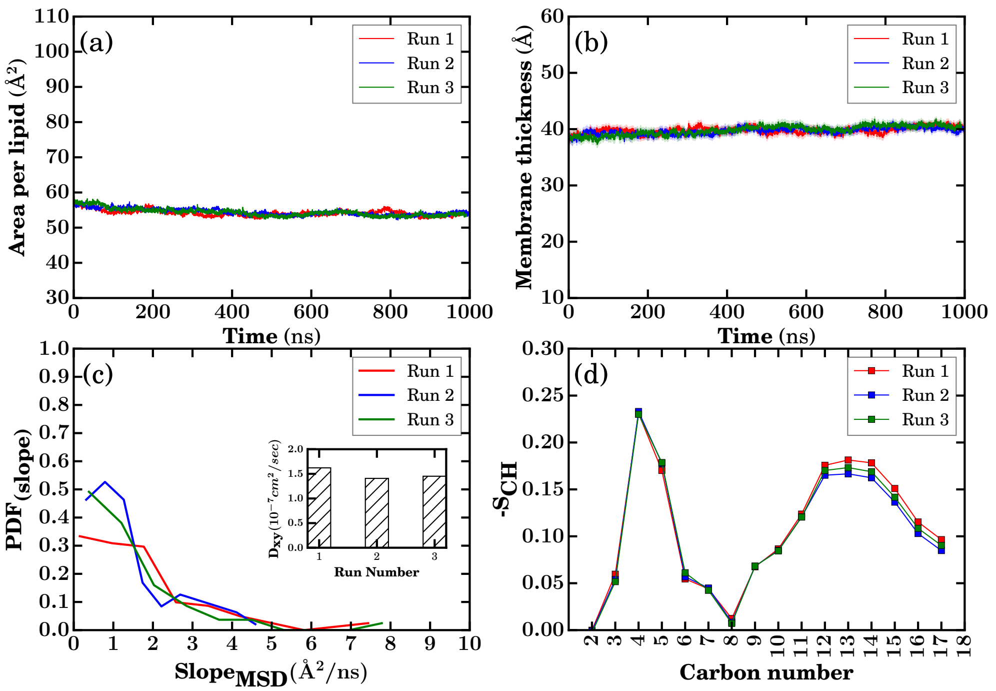

Figure S11: Effect of 1.50 mol% Ethanol on membrane properties (a) Area per lipid, (b) Membrane thickness, (c) Distribution of MSD slopes calculated at 10 ns chunks from the MD trajectory with the inset showing the lateral diffusion coefficient (D_{xy}), (d) Deuterium order parameter ($-S_{\mathrm{CH}}$), in an NpT ensemble at 300 K and 1 bar.

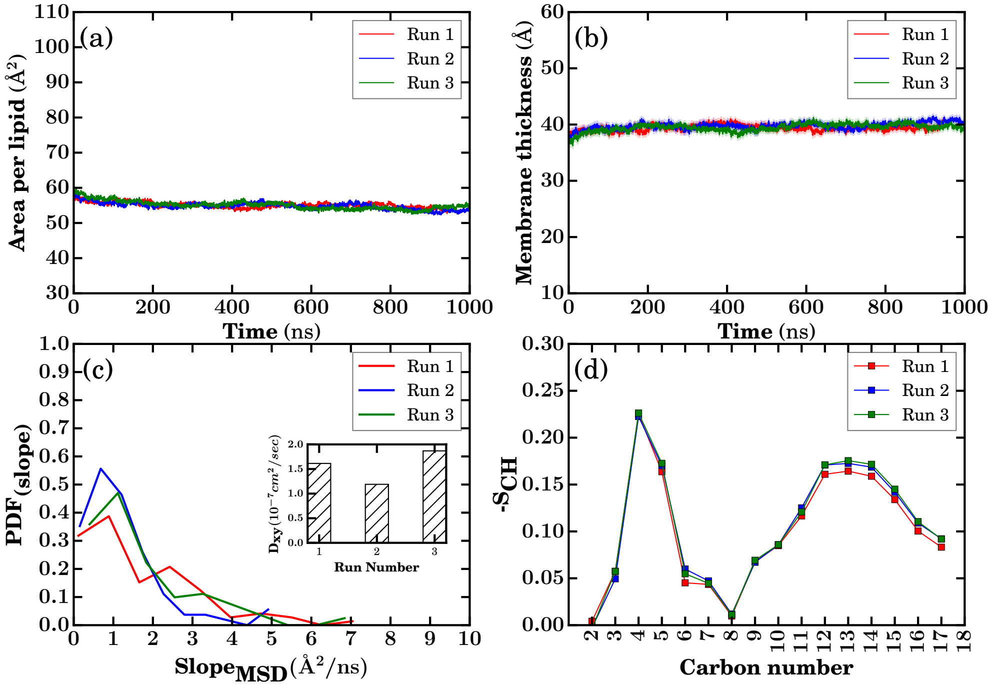

Figure S12: Effect of 2.00 mol% Ethanol on membrane properties (a) Area per lipid, (b) Membrane thickness, (c) Distribution of MSD slopes calculated at 10 ns chunks from the MD trajectory with the inset showing the lateral diffusion coefficient (D_{xy}), (d) Deuterium order parameter ($-S_{\mathrm{CH}}$), in an NpT ensemble at 300 K and 1 bar.

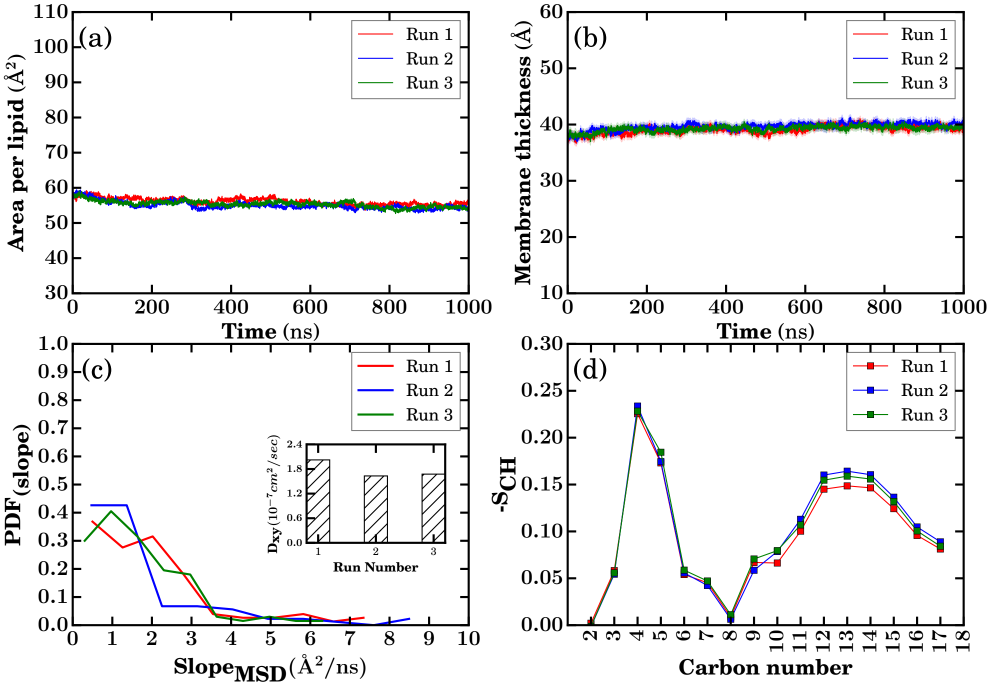

Figure S13: Effect of 2.50 mol% Ethanol on membrane properties (a) Area per lipid, (b) Membrane thickness, (c) Distribution of MSD slopes calculated at 10 ns chunks from the MD trajectory with the inset showing the lateral diffusion coefficient (D_{xy}), (d) Deuterium order parameter ($-S_{\mathrm{CH}}$), in an NpT ensemble at 300 K and 1 bar.

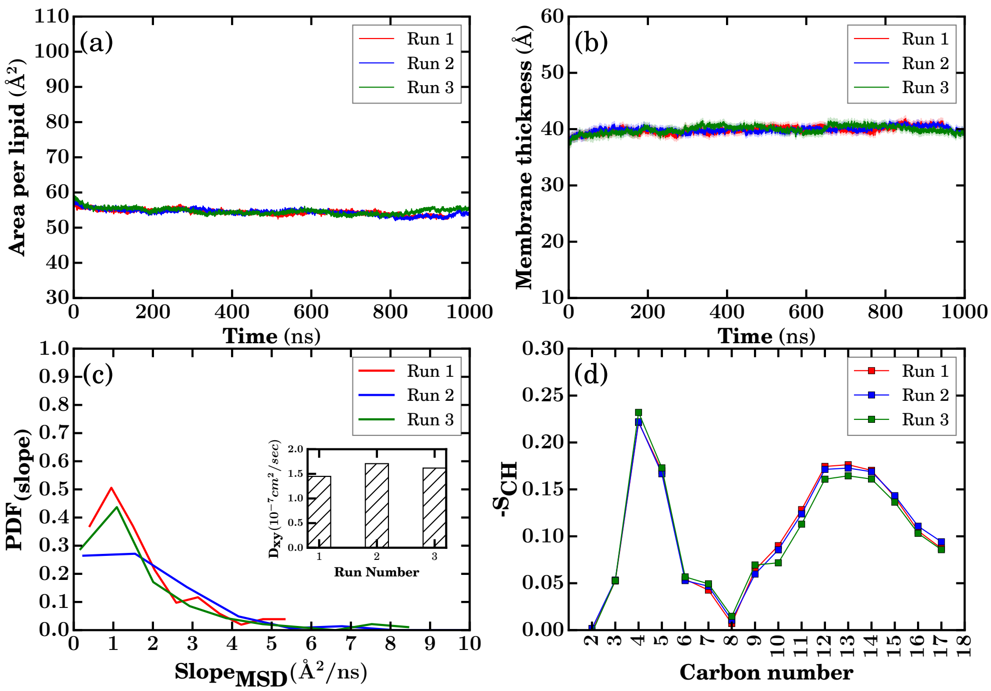

Figure S14: Effect of 0.50 mol% Furfural on membrane properties (a) Area per lipid, (b) Membrane thickness, (c) Distribution of MSD slopes calculated at 10 ns chunks from the MD trajectory with the inset showing the lateral diffusion coefficient (D_{xy}), (d) Deuterium order parameter ($-S_{\mathrm{CH}}$), in an NpT ensemble at 300 K and 1 bar.

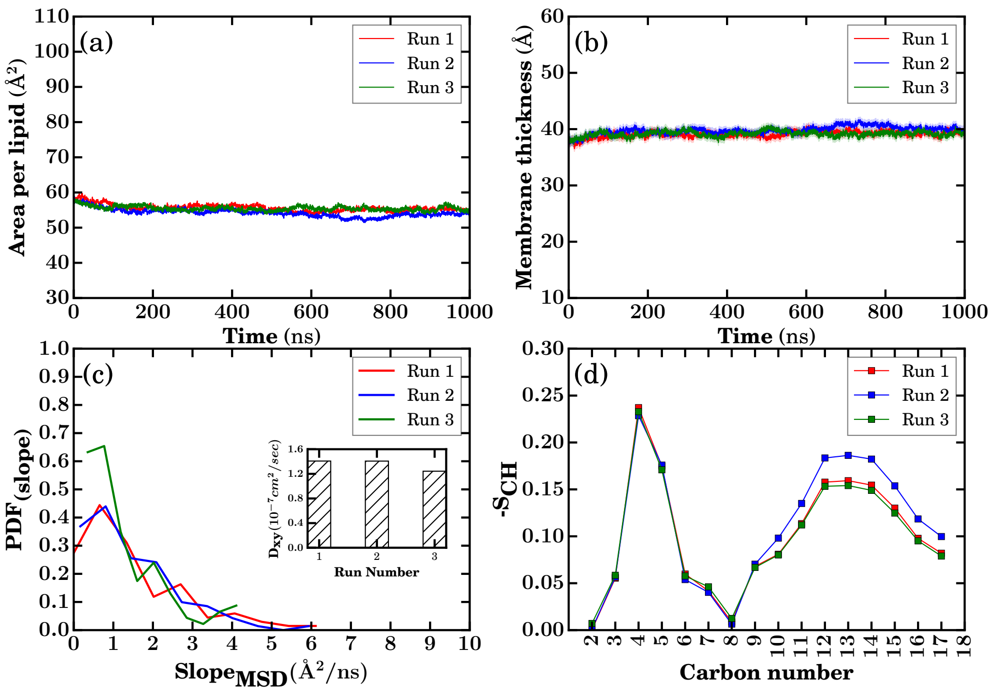

Figure S15: Effect of 1.00 mol% Furfural on membrane properties (a) Area per lipid, (b) Membrane thickness, (c) Distribution of MSD slopes calculated at 10 ns chunks from the MD trajectory with the inset showing the lateral diffusion coefficient (D_{xy}), (d) Deuterium order parameter ($-S_{\mathrm{CH}}$), in an NpT ensemble at 300 K and 1 bar.

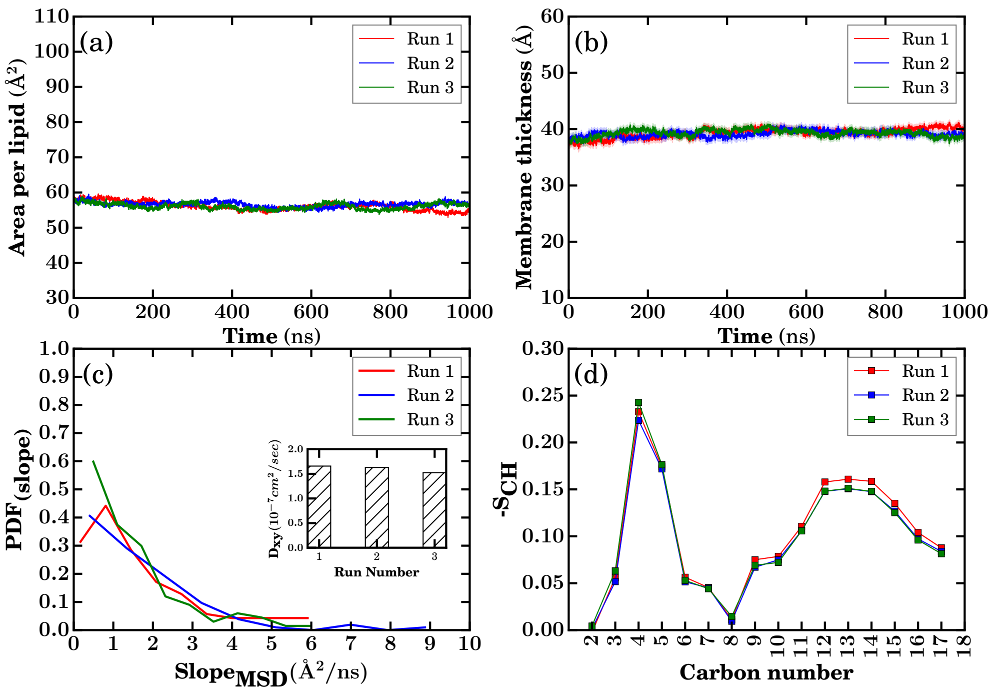

Figure S16: Effect of 1.50 mol% Furfural on membrane properties (a) Area per lipid, (b) Membrane thickness, (c) Distribution of MSD slopes calculated at 10 ns chunks from the MD trajectory with the inset showing the lateral diffusion coefficient (D_{xy}), (d) Deuterium order parameter ($-S_{\mathrm{CH}}$), in an NpT ensemble at 300 K and 1 bar.

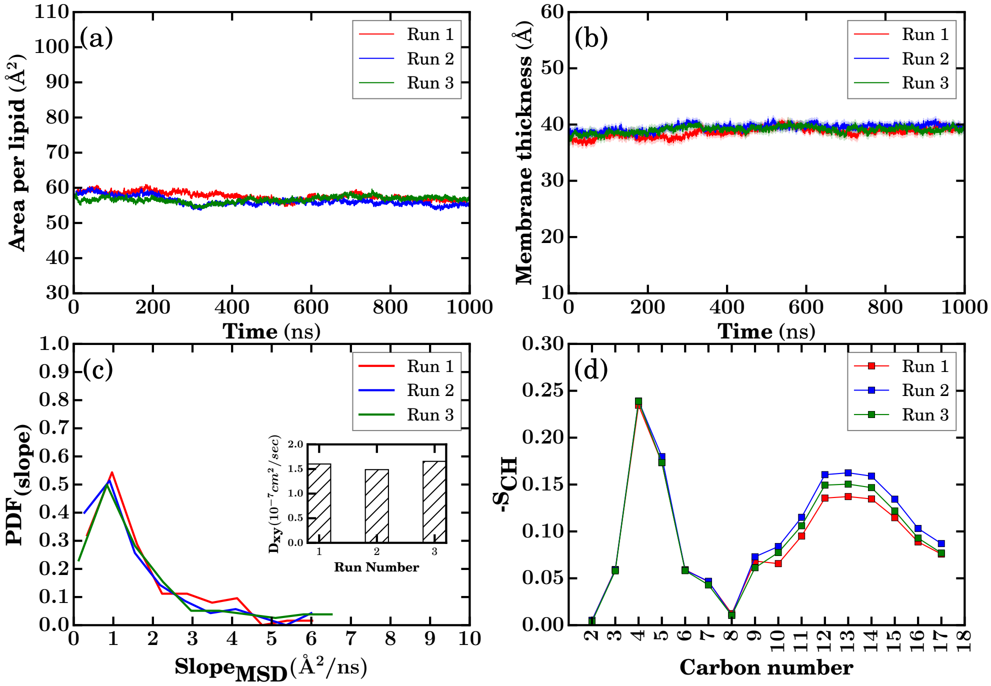

Figure S17: Effect of 2.00 mol% Furfural on membrane properties (a) Area per lipid, (b) Membrane thickness, (c) Distribution of MSD slopes calculated at 10 ns chunks from the MD trajectory with the inset showing the lateral diffusion coefficient (D_{xy}), (d) Deuterium order parameter ($-S_{\mathrm{CH}}$), in an NpT ensemble at 300 K and 1 bar.

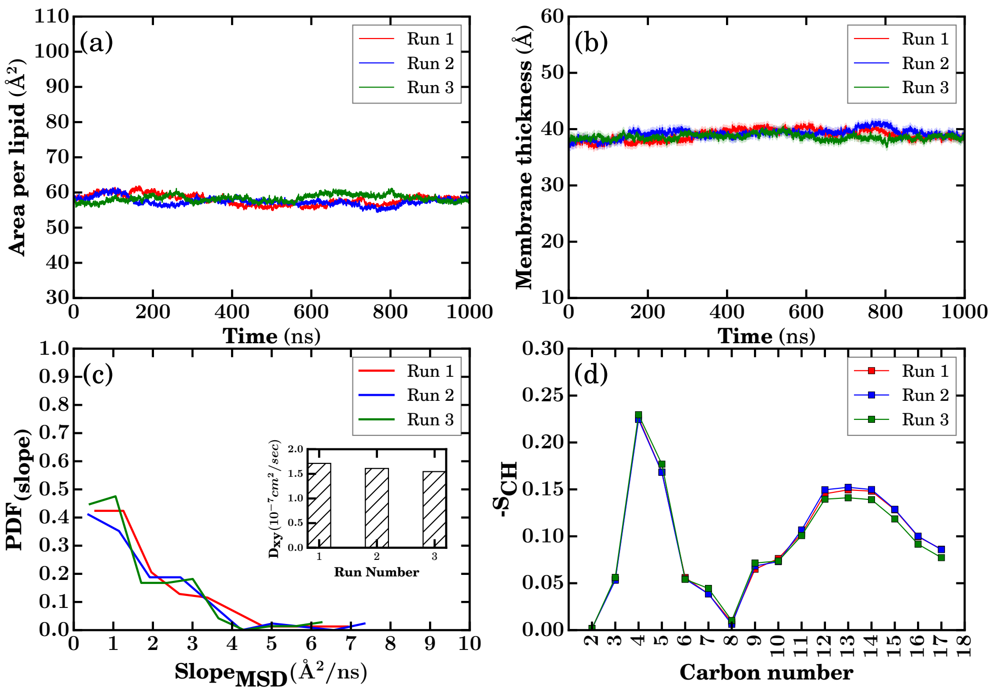

Figure S18: Effect of 2.50 mol% Furfural on membrane properties (a) Area per lipid, (b) Membrane thickness, (c) Distribution of MSD slopes calculated at 10 ns chunks from the MD trajectory with the inset showing the lateral diffusion coefficient (D_{xy}), (d) Deuterium order parameter ($-S_{\mathrm{CH}}$), in an NpT ensemble at 300 K and 1 bar.

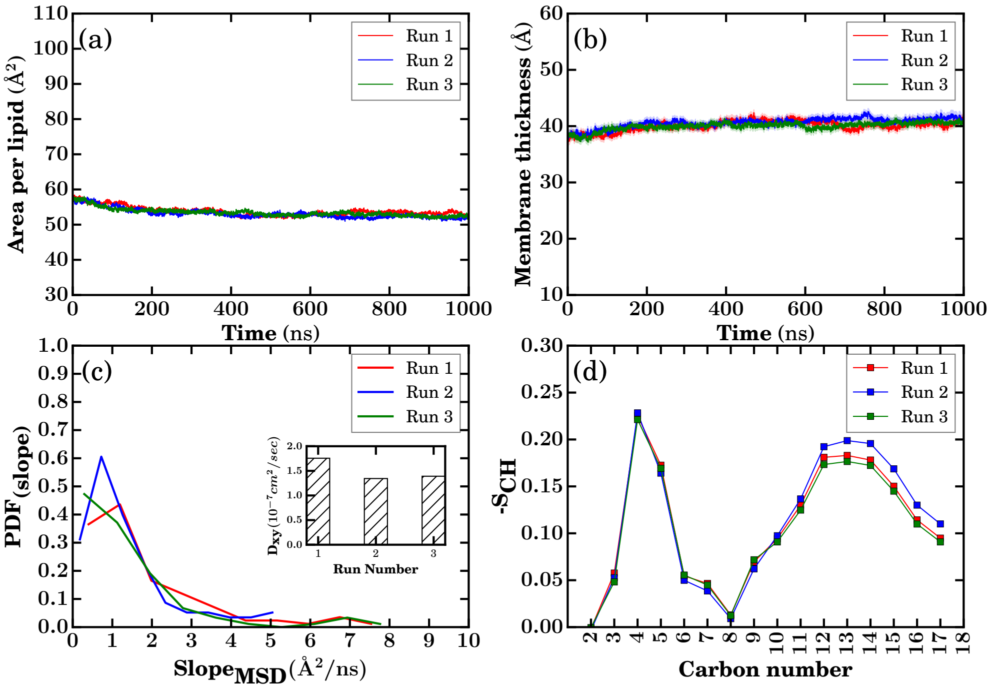

Figure S19: Effect of 0.50 mol% Acetone on membrane properties (a) Area per lipid, (b) Membrane thickness, (c) Distribution of MSD slopes calculated at 10 ns chunks from the MD trajectory with the inset showing the lateral diffusion coefficient (D_{xy}), (d) Deuterium order parameter ($-S_{\mathrm{CH}}$), in an NpT ensemble at 300 K and 1 bar.

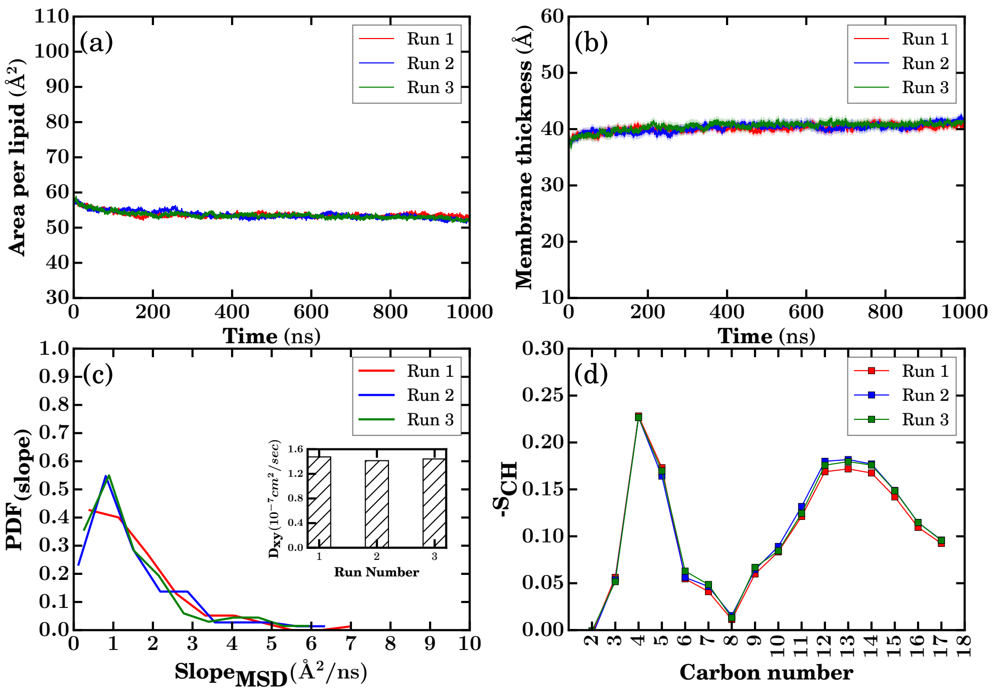

Figure S20: Effect of 1.00 mol% Acetone on membrane properties (a) Area per lipid, (b) Membrane thickness, (c) Distribution of MSD slopes calculated at 10 ns chunks from the MD trajectory with the inset showing the lateral diffusion coefficient (D_{xy}), (d) Deuterium order parameter ($-S_{\rm CH}$), in an NpT ensemble at 300 K and 1 bar.

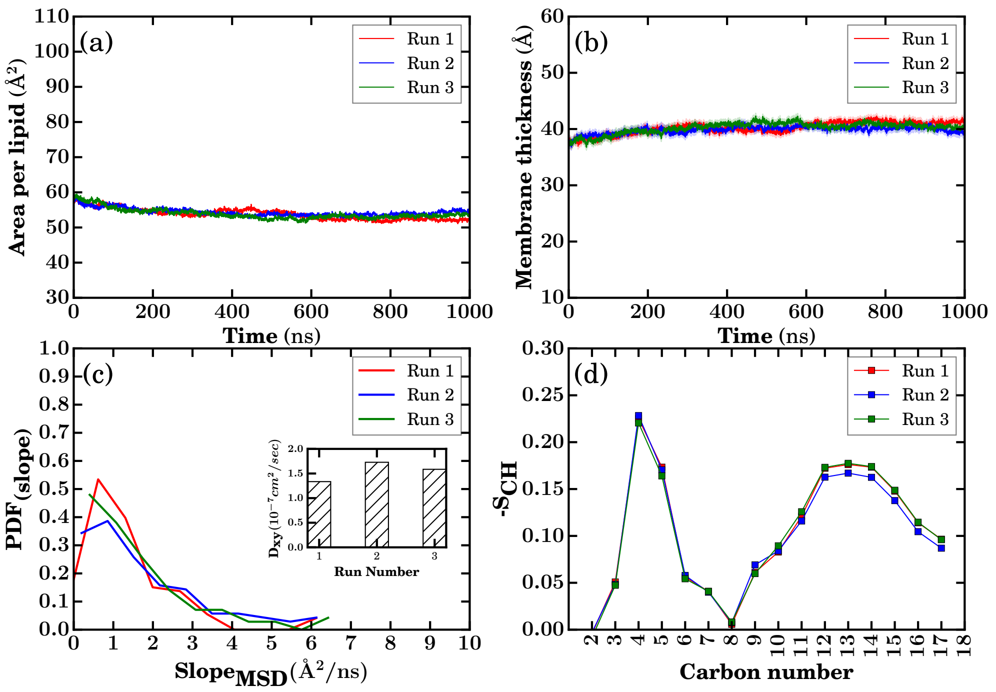

Figure S21: Effect of 1.50 mol% Acetone on membrane properties (a) Area per lipid, (b) Membrane thickness, (c) Distribution of MSD slopes calculated at 10 ns chunks from the MD trajectory with the inset showing the lateral diffusion coefficient (D_{xy}), (d) Deuterium order parameter ($-S_{\mathrm{CH}}$), in an NpT ensemble at 300 K and 1 bar.

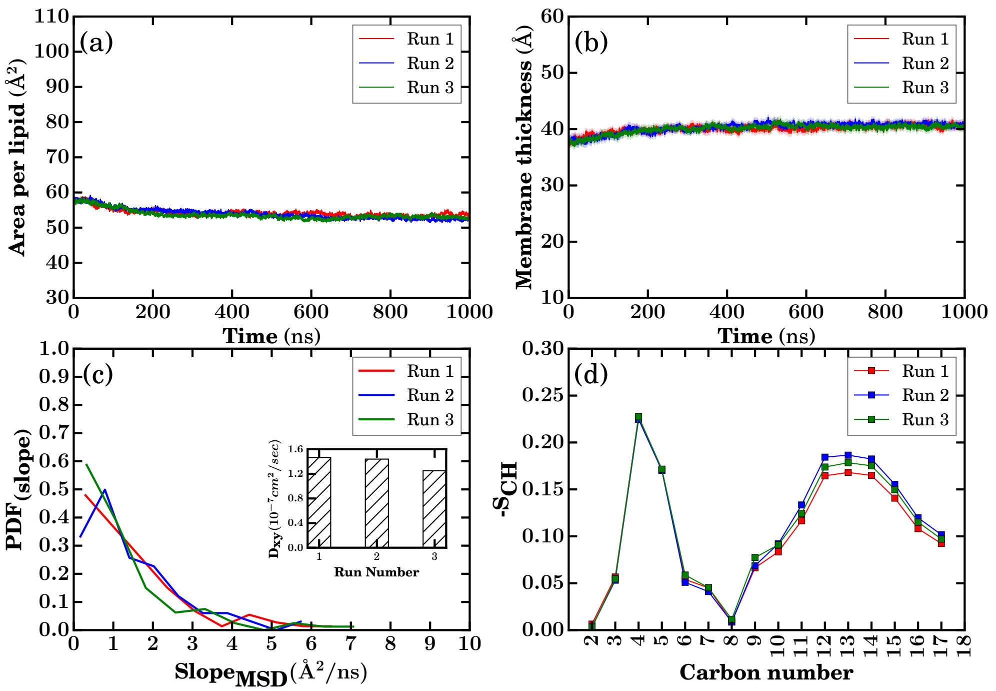

Figure S22: Effect of 2.00 mol% Acetone on membrane properties (a) Area per lipid, (b) Membrane thickness, (c) Distribution of MSD slopes calculated at 10 ns chunks from the MD trajectory with the inset showing the lateral diffusion coefficient (D_{xy}), (d) Deuterium order parameter ($-S_{\rm CH}$), in an NpT ensemble at 300 K and 1 bar.

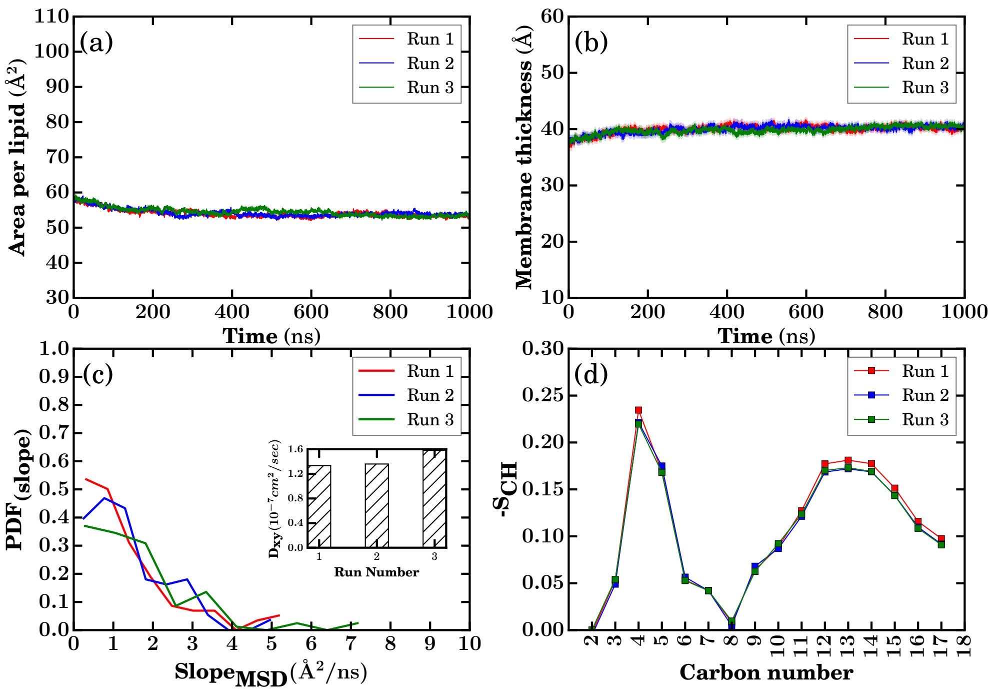

Figure S23: Effect of 2.50 mol% Acetone on membrane properties (a) Area per lipid, (b) Membrane thickness, (c) Distribution of MSD slopes calculated at 10 ns chunks from the MD trajectory with the inset showing the lateral diffusion coefficient (D_{xy}), (d) Deuterium order parameter ($-S_{\mathrm{CH}}$), in an NpT ensemble at 300 K and 1 bar.

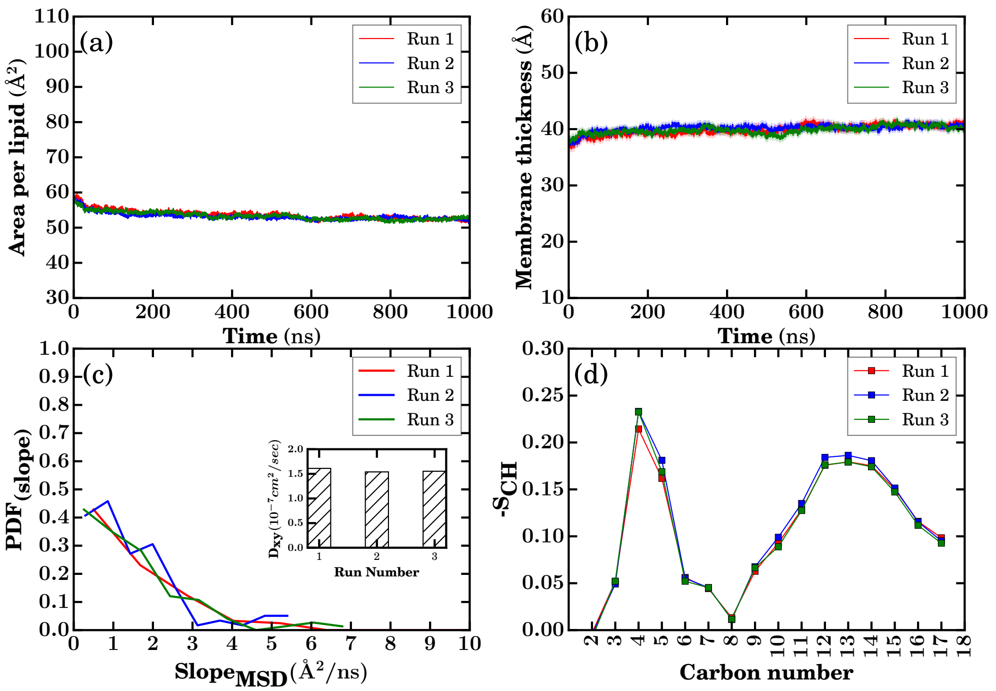

Figure S24: Effect of 0.50 mol% Acetaldehyde on membrane properties (a) Area per lipid, (b) Membrane thickness, (c) Distribution of MSD slopes calculated at 10 ns chunks from the MD trajectory with the inset showing the lateral diffusion coefficient (D_{xy}), (d) Deuterium order parameter ($-S_{\mathrm{CH}}$), in an NpT ensemble at 300 K and 1 bar.

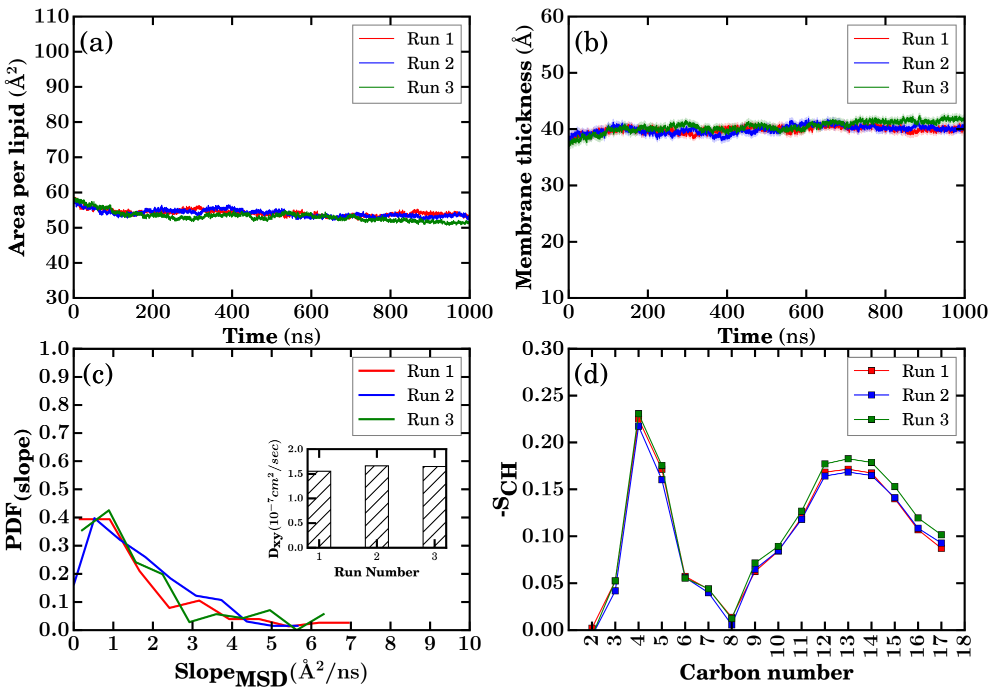

Figure S25: Effect of 1.00 mol% Acetaldehyde on membrane properties (a) Area per lipid, (b) Membrane thickness, (c) Distribution of MSD slopes calculated at 10 ns chunks from the MD trajectory with the inset showing the lateral diffusion coefficient (D_{xy}), (d) Deuterium order parameter ($-S_{\mathrm{CH}}$), in an NpT ensemble at 300 K and 1 bar.

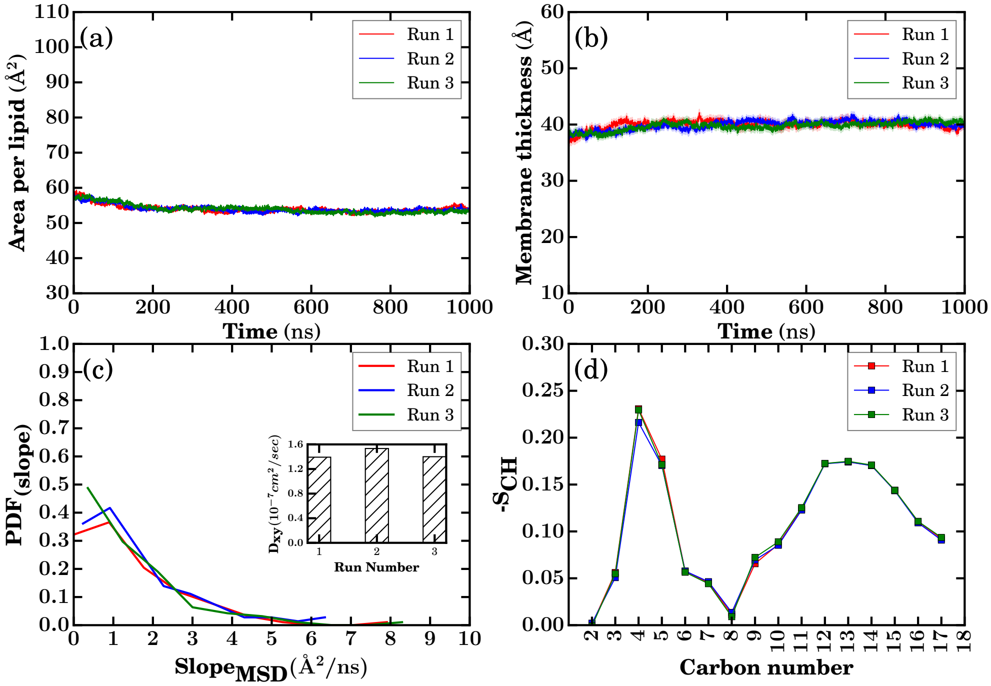

Figure S26: Effect of 1.50 mol% Acetaldehyde on membrane properties (a) Area per lipid, (b) Membrane thickness, (c) Distribution of MSD slopes calculated at 10 ns chunks from the MD trajectory with the inset showing the lateral diffusion coefficient (D_{xy}), (d) Deuterium order parameter ($-S_{\text{CH}}$), in an NpT ensemble at 300 K and 1 bar.

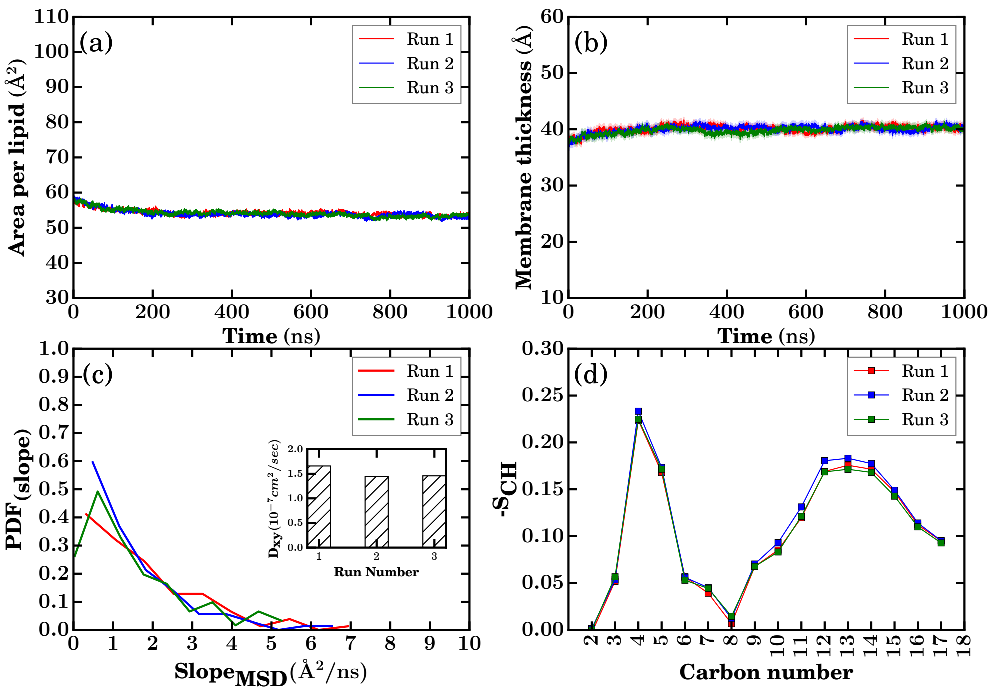

Figure S27: Effect of 2.00 mol% Acetaldehyde on membrane properties (a) Area per lipid, (b) Membrane thickness, (c) Distribution of MSD slopes calculated at 10 ns chunks from the MD trajectory with the inset showing the lateral diffusion coefficient (D_{xy}), (d) Deuterium order parameter ($-S_{\mathrm{CH}}$), in an NpT ensemble at 300 K and 1 bar.

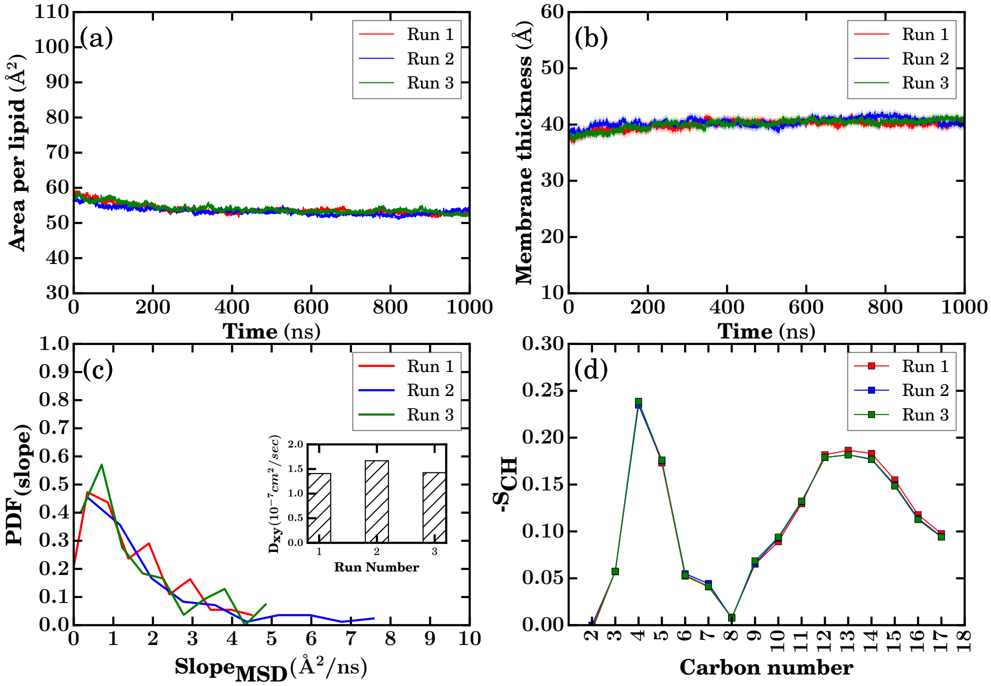

Figure S28: Effect of 2.50 mol% Acetaldehyde on membrane properties (a) Area per lipid, (b) Membrane thickness, (c) Distribution of MSD slopes calculated at 10 ns chunks from the MD trajectory with the inset showing the lateral diffusion coefficient (D_{xy}), (d) Deuterium order parameter ($-S_{\mathrm{CH}}$), in an NpT ensemble at 300 K and 1 bar.

Effect of Chain Length on Membrane Dynamics

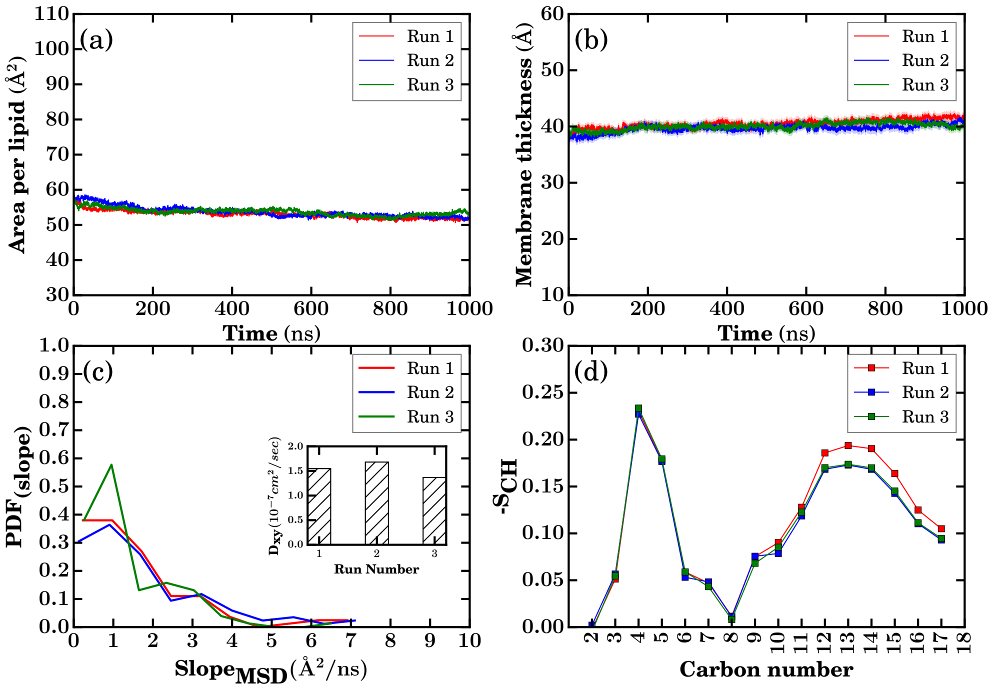

Figure S29: Effect of 0.50 mol% HMF on membrane properties (a) Area per lipid, (b) Membrane thickness, (c) Distribution of MSD slopes calculated at 10 ns chunks from the MD trajectory with the inset showing the lateral diffusion coefficient (D_{xy}), (d) Deuterium order parameter ($-S_{\mathrm{CH}}$), in an NpT ensemble at 300 K and 1 bar.

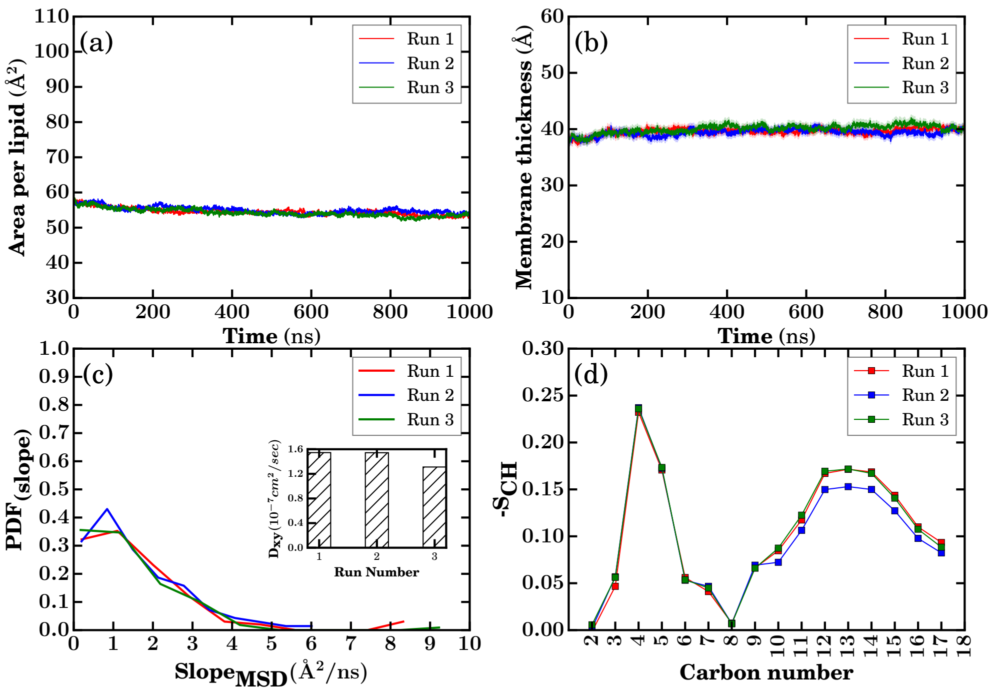

Figure S30: Effect of 1.00 mol% HMF on membrane properties (a) Area per lipid, (b) Membrane thickness, (c) Distribution of MSD slopes calculated at 10 ns chunks from the MD trajectory with the inset showing the lateral diffusion coefficient (D_{xy}), (d) Deuterium order parameter ($-S_{\mathrm{CH}}$), in an NpT ensemble at 300 K and 1 bar.

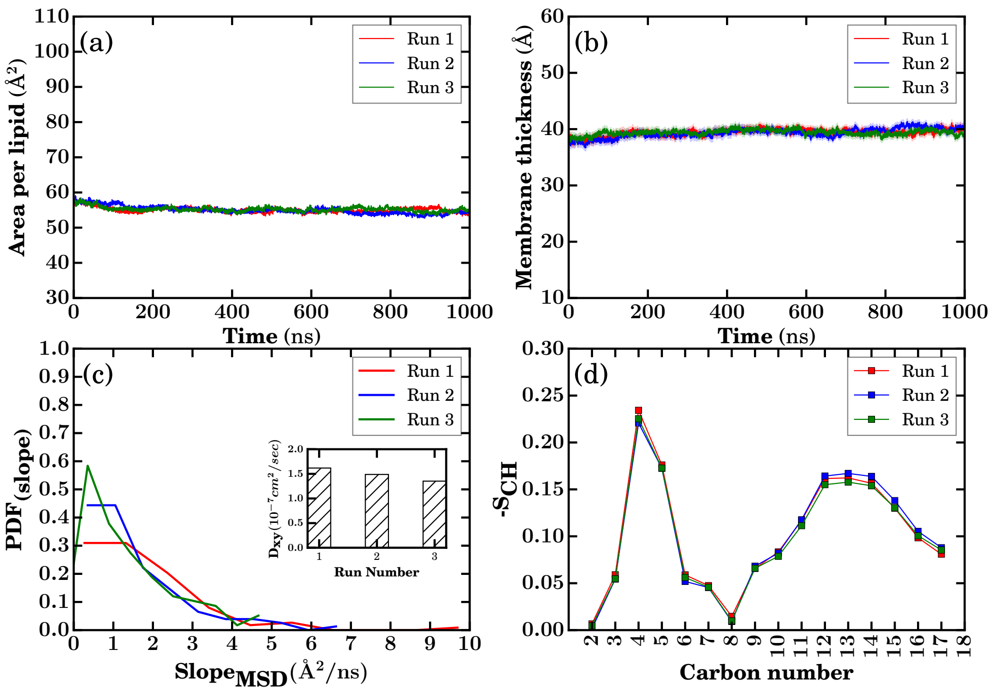

Figure S31: Effect of 1.50 mol% HMF on membrane properties (a) Area per lipid, (b) Membrane thickness, (c) Distribution of MSD slopes calculated at 10 ns chunks from the MD trajectory with the inset showing the lateral diffusion coefficient (D_{xy}), (d) Deuterium order parameter ($-S_{\mathrm{CH}}$), in an NpT ensemble at 300 K and 1 bar.

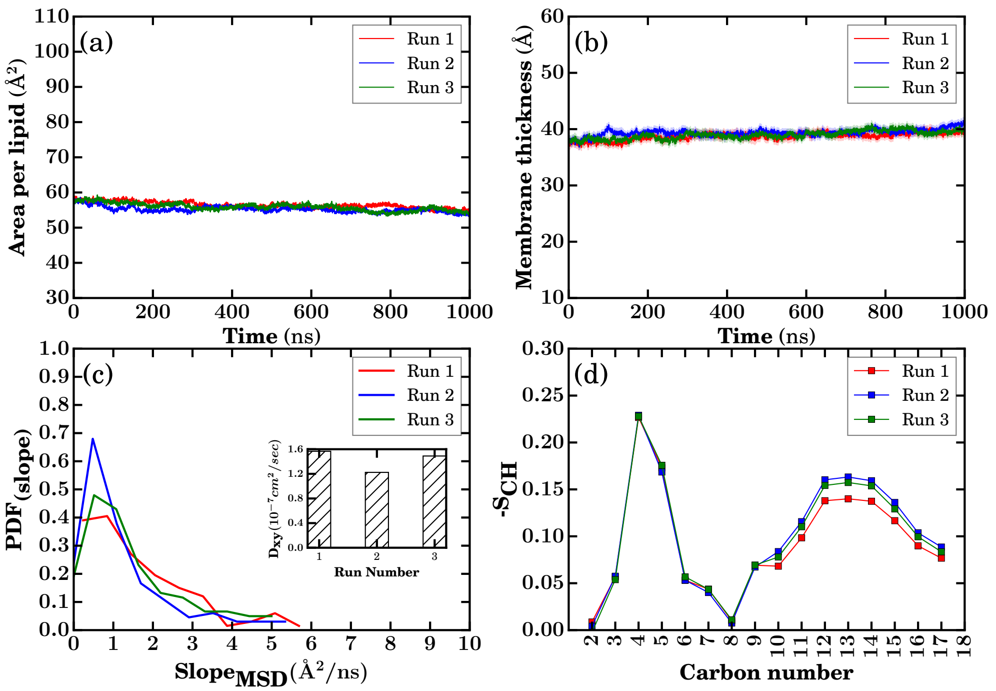

Figure S32: Effect of 2.00 mol% HMF on membrane properties (a) Area per lipid, (b) Membrane thickness, (c) Distribution of MSD slopes calculated at 10 ns chunks from the MD trajectory with the inset showing the lateral diffusion coefficient (D_{xy}), (d) Deuterium order parameter ($-S_{\mathrm{CH}}$), in an NpT ensemble at 300 K and 1 bar.

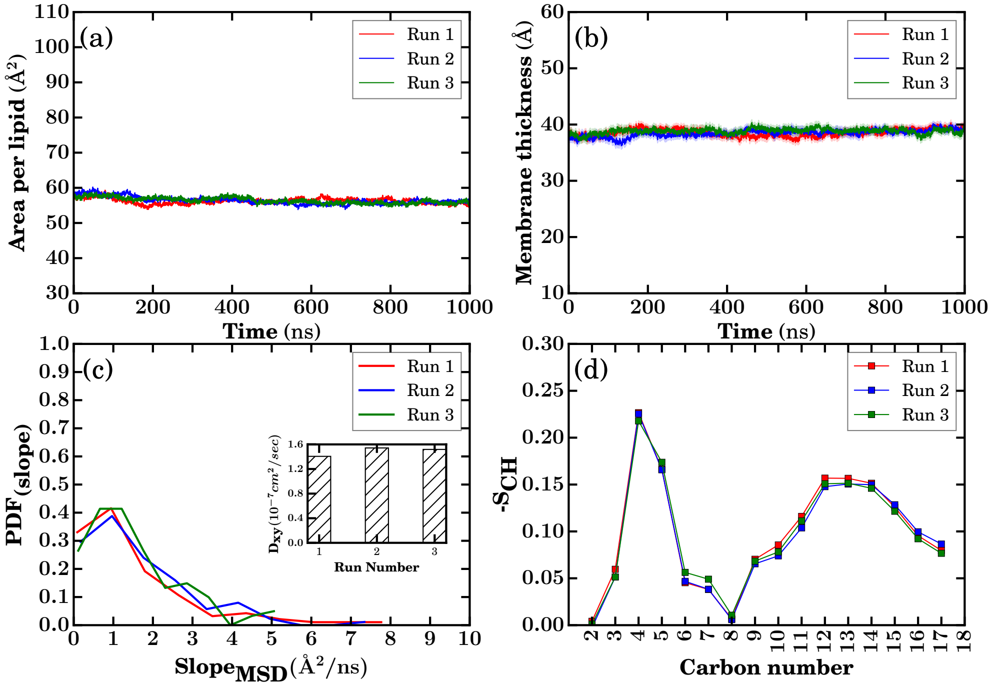

Figure S33: Effect of 2.50 mol% HMF on membrane properties (a) Area per lipid, (b) Membrane thickness, (c) Distribution of MSD slopes calculated at 10 ns chunks from the MD trajectory with the inset showing the lateral diffusion coefficient (D_{xy}), (d) Deuterium order parameter ($-S_{\mathrm{CH}}$), in an NpT ensemble at 300 K and 1 bar.

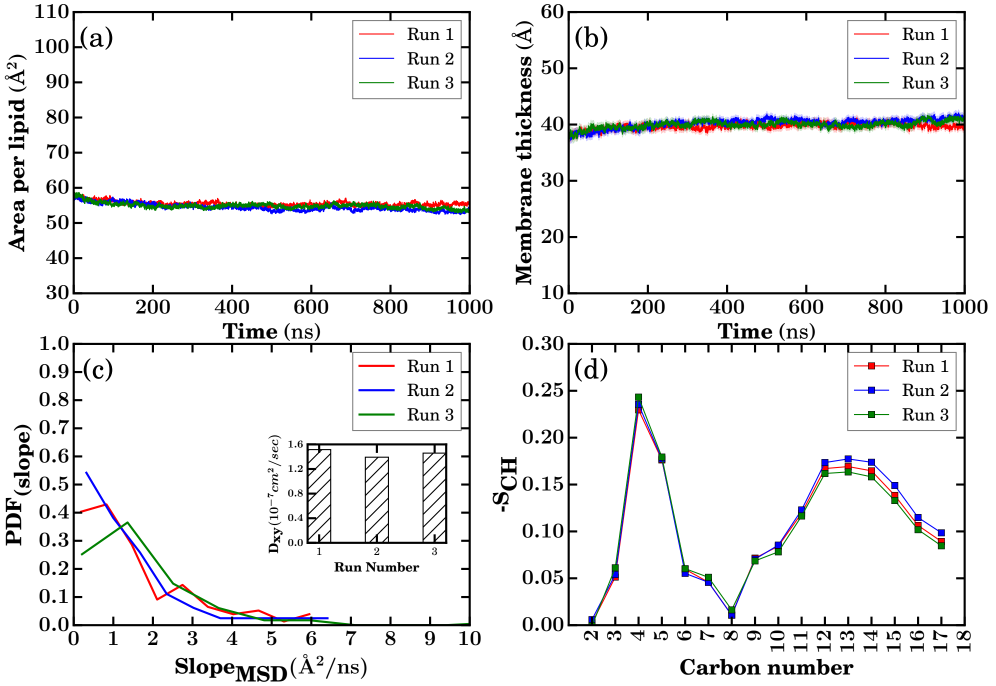

Figure S34: Effect of 0.50 mol% Isobutanol on membrane properties (a) Area per lipid, (b) Membrane thickness, (c) Distribution of MSD slopes calculated at 10 ns chunks from the MD trajectory with the inset showing the lateral diffusion coefficient (D_{xy}), (d) Deuterium order parameter ($-S_{\mathrm{CH}}$), in an NpT ensemble at 300 K and 1 bar.

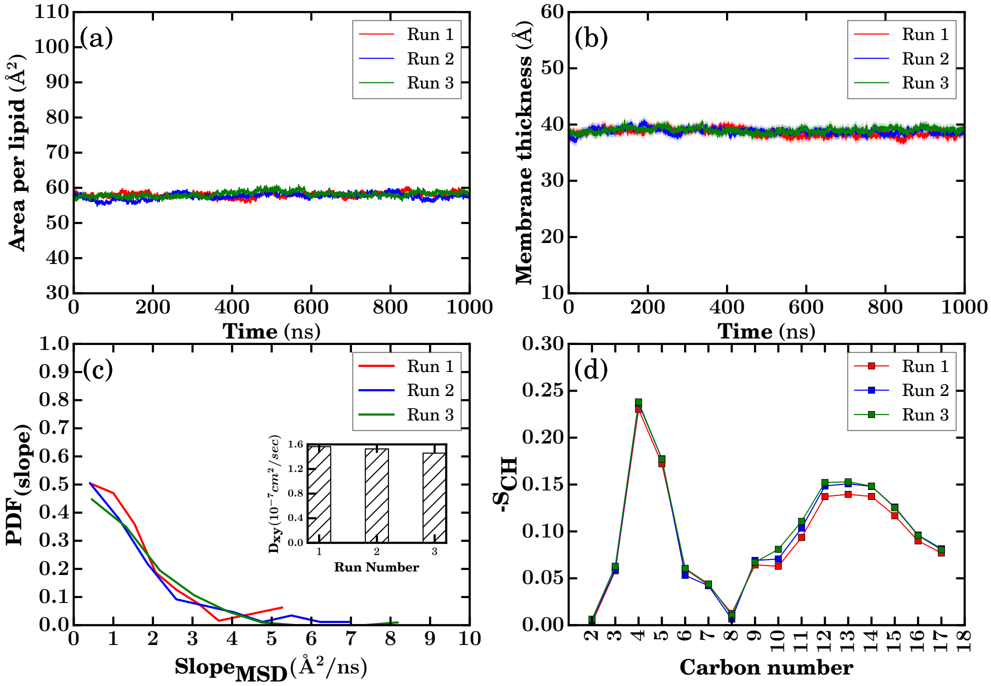

Figure S35: Effect of 1.00 mol% Isobutanol on membrane properties (a) Area per lipid, (b) Membrane thickness, (c) Distribution of MSD slopes calculated at 10 ns chunks from the MD trajectory with the inset showing the lateral diffusion coefficient (D_{xy}), (d) Deuterium order parameter ($-S_{\mathrm{CH}}$), in an NpT ensemble at 300 K and 1 bar.

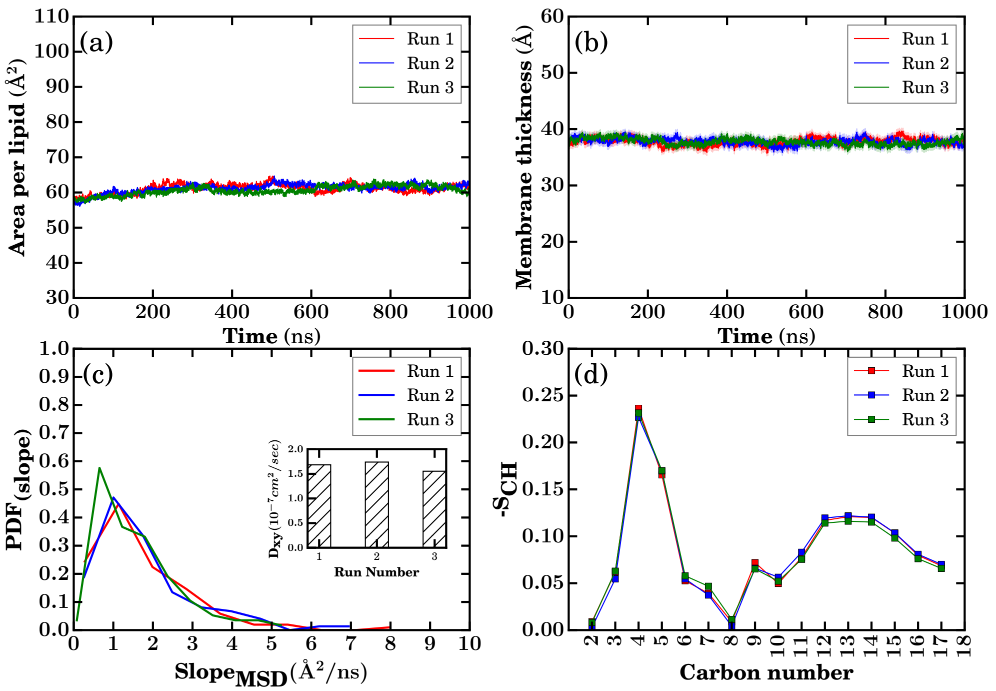

Figure S36: Effect of 1.50 mol% Isobutanol on membrane properties (a) Area per lipid, (b) Membrane thickness, (c) Distribution of MSD slopes calculated at 10 ns chunks from the MD trajectory with the inset showing the lateral diffusion coefficient (D_{xy}), (d) Deuterium order parameter ($-S_{\mathrm{CH}}$), in an NpT ensemble at 300 K and 1 bar.

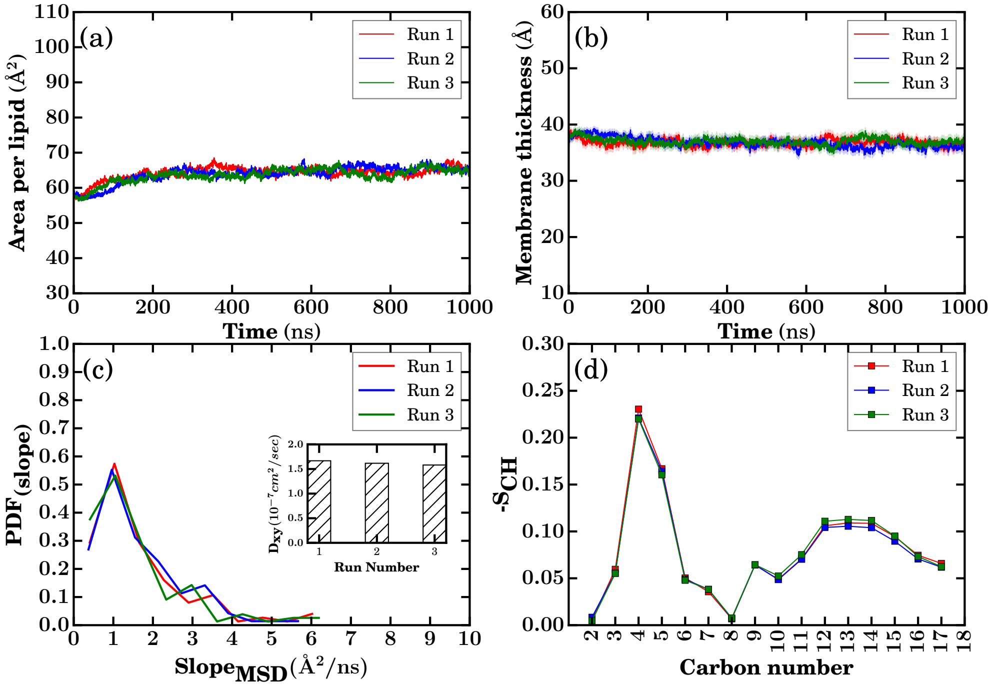

Figure S37: Effect of 2.00 mol% Isobutanol on membrane properties (a) Area per lipid, (b) Membrane thickness, (c) Distribution of MSD slopes calculated at 10 ns chunks from the MD trajectory with the inset showing the lateral diffusion coefficient (D_{xy}), (d) Deuterium order parameter ($-S_{\mathrm{CH}}$), in an NpT ensemble at 300 K and 1 bar.

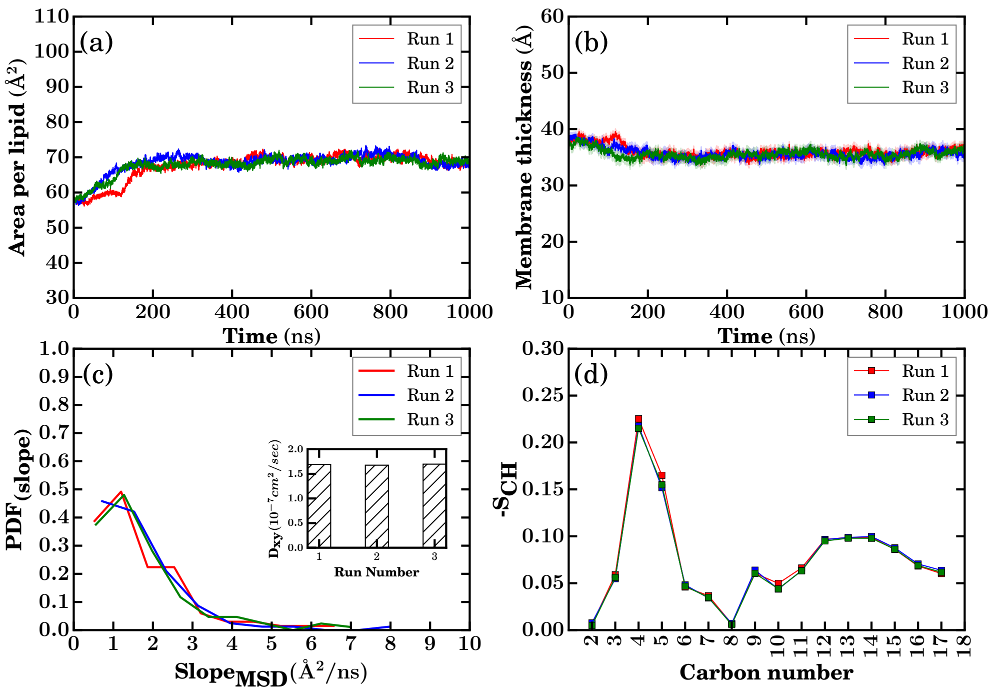

Figure S38: Effect of 2.50 mol% Isobutanol on membrane properties (a) Area per lipid, (b) Membrane thickness, (c) Distribution of MSD slopes calculated at 10 ns chunks from the MD trajectory with the inset showing the lateral diffusion coefficient (D_{xy}), (d) Deuterium order parameter ($-S_{\mathrm{CH}}$), in an NpT ensemble at 300 K and 1 bar.

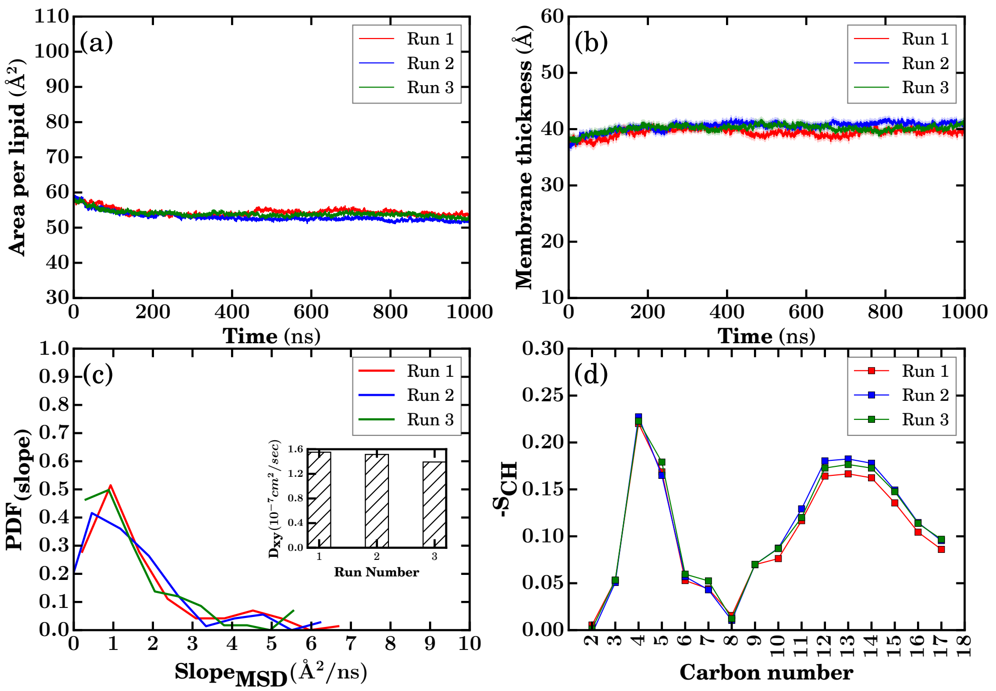

Figure S39: Effect of 0.50 mol% Formic acid on membrane properties (a) Area per lipid, (b) Membrane thickness, (c) Distribution of MSD slopes calculated at 10 ns chunks from the MD trajectory with the inset showing the lateral diffusion coefficient (D_{xy}), (d) Deuterium order parameter ($-S_{\text{CH}}$), in an NpT ensemble at 300 K and 1 bar.

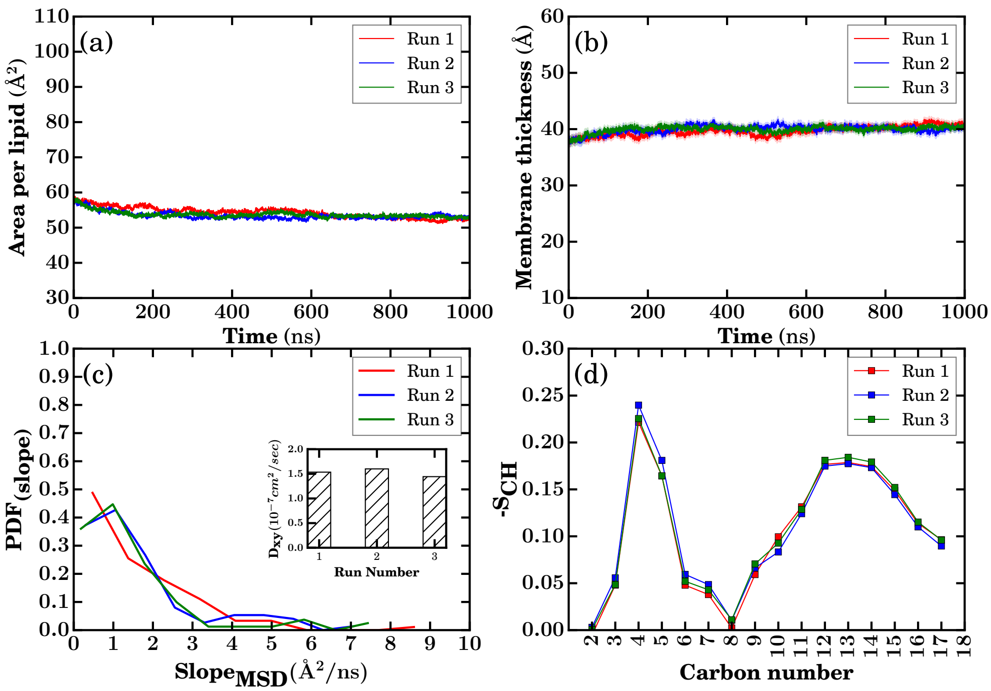

Figure S40: Effect of 1.00 mol% Formic acid on membrane properties (a) Area per lipid, (b) Membrane thickness, (c) Distribution of MSD slopes calculated at 10 ns chunks from the MD trajectory with the inset showing the lateral diffusion coefficient (D_{xy}), (d) Deuterium order parameter ($-S_{\mathrm{CH}}$), in an NpT ensemble at 300 K and 1 bar.

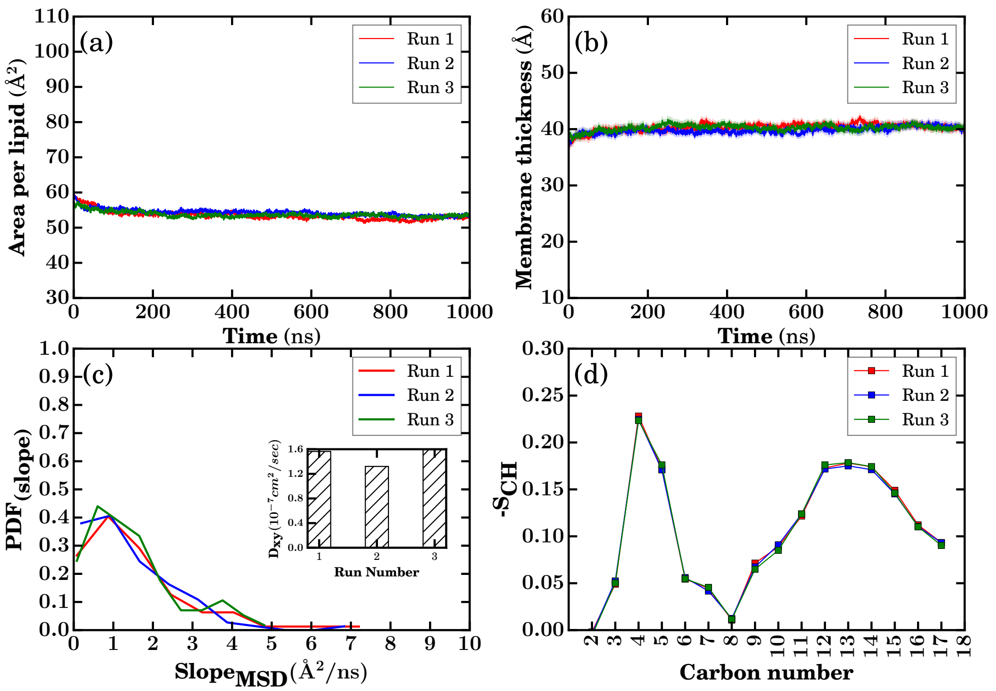

Figure S41: Effect of 1.50 mol% Formic acid on membrane properties (a) Area per lipid, (b) Membrane thickness, (c) Distribution of MSD slopes calculated at 10 ns chunks from the MD trajectory with the inset showing the lateral diffusion coefficient (D_{xy}), (d) Deuterium order paramet XXXX er ($-S_{\mathrm{CH}}$), in an NpT ensemble at 300 K and 1 bar.

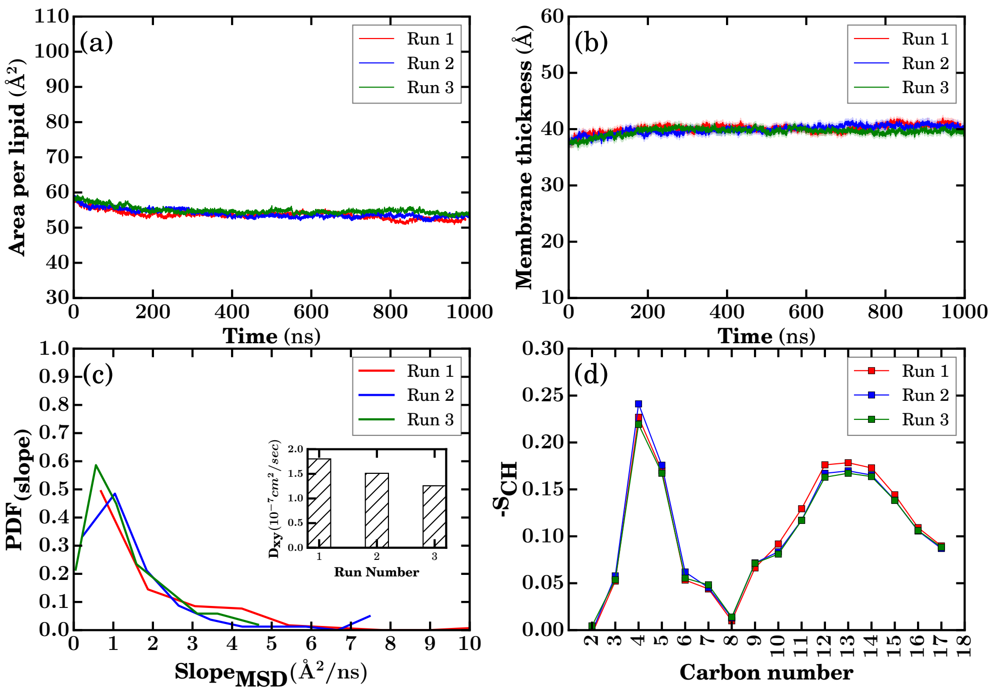

Figure S42: Effect of 2.00 mol% Formic acid on membrane properties (a) Area per lipid, (b) Membrane thickness, (c) Distribution of MSD slopes calculated at 10 ns chunks from the MD trajectory with the inset showing the lateral diffusion coefficient (D_{xy}), (d) Deuterium order parameter ($-S_{\mathrm{CH}}$), in an NpT ensemble at 300 K and 1 bar.

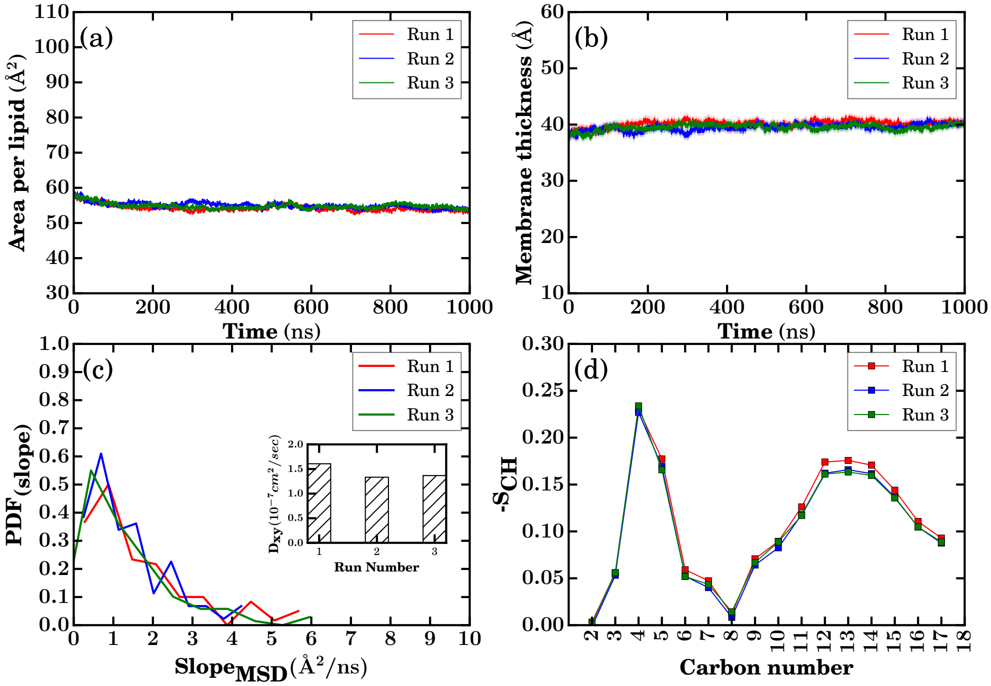

Figure S43: Effect of 2.50 mol% Formic acid on membrane properties (a) Area per lipid, (b) Membrane thickness, (c) Distribution of MSD slopes calculated at 10 ns chunks from the MD trajectory with the inset showing the lateral diffusion coefficient (D_{xy}), (d) Deuterium order parameter ($-S_{\mathrm{CH}}$), in an NpT ensemble at 300 K and 1 bar.

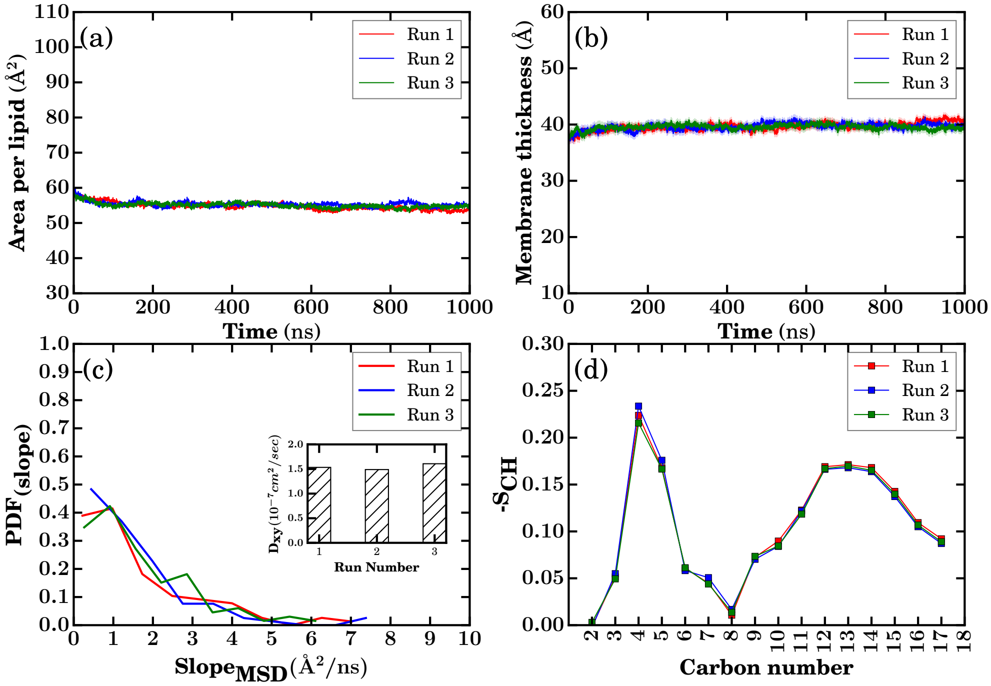

Figure S44: Effect of 0.50 mol% Propanoic acid on membrane properties (a) Area per lipid, (b) Membrane thickness, (c) Distribution of MSD slopes calculated at 10 ns chunks from the MD trajectory with the inset showing the lateral diffusion coefficient (D_{xy}), (d) Deuterium order parameter ($-S_{\mathrm{CH}}$), in an NpT ensemble at 300 K and 1 bar.

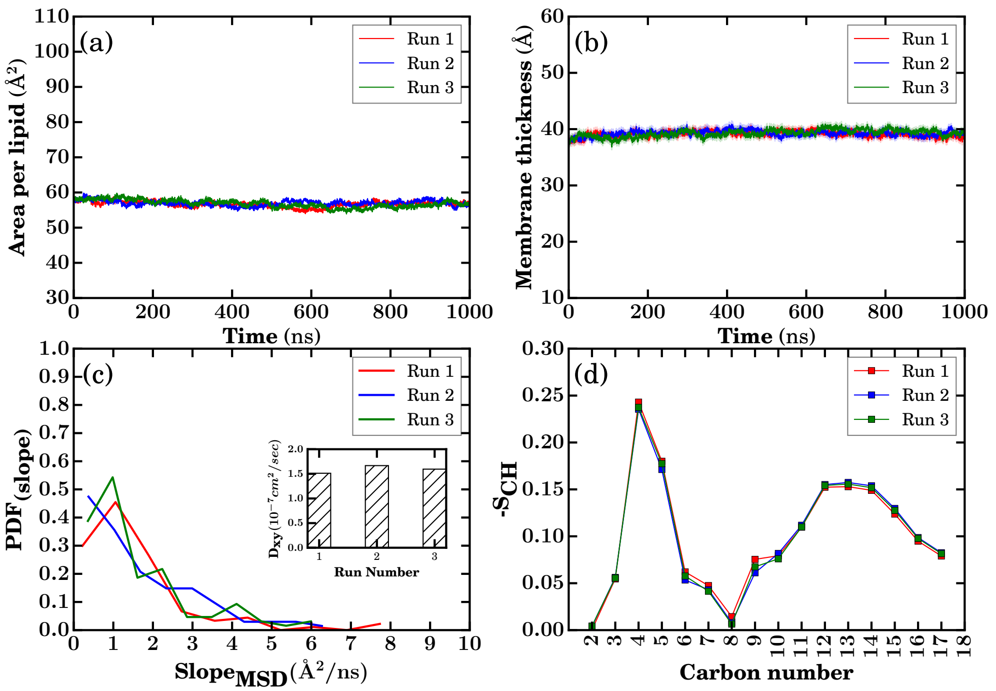

Figure S45: Effect of 1.00 mol% Propanoic acid on membrane properties (a) Area per lipid, (b) Membrane thickness, (c) Distribution of MSD slopes calculated at 10 ns chunks from the MD trajectory with the inset showing the lateral diffusion coefficient (D_{xy}), (d) Deuterium order parameter ($-S_{\mathrm{CH}}$), in an NpT ensemble at 300 K and 1 bar.

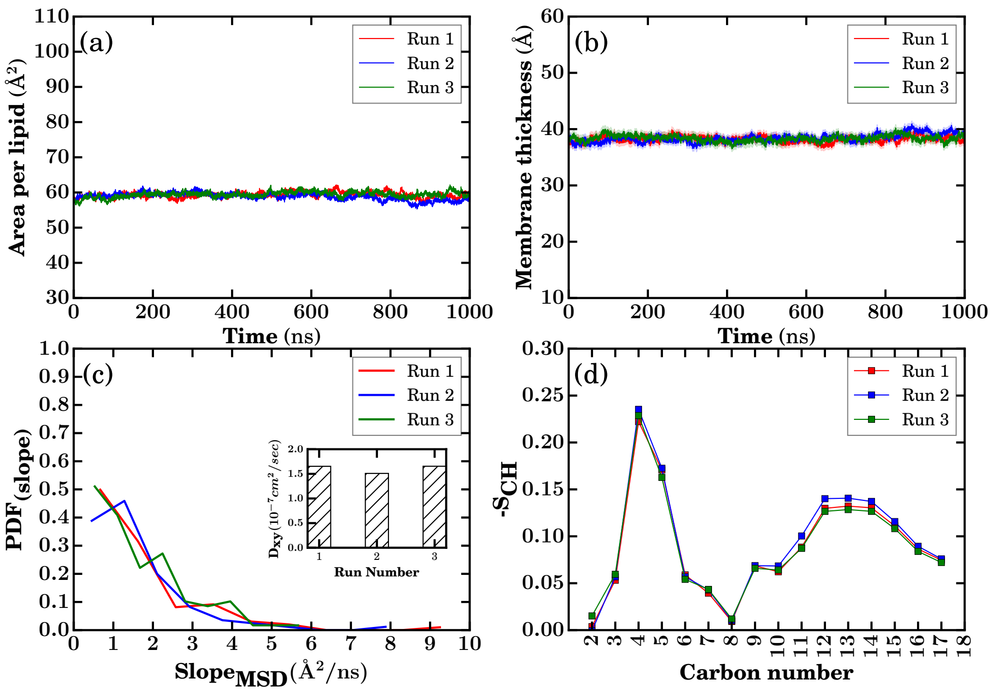

Figure S46: Effect of 1.50 mol% Propanoic acid on membrane properties (a) Area per lipid, (b) Membrane thickness, (c) Distribution of MSD slopes calculated at 10 ns chunks from the MD trajectory with the inset showing the lateral diffusion coefficient (D_{xy}), (d) Deuterium order parameter ($-S_{\mathrm{CH}}$), in an NpT ensemble at 300 K and 1 bar.

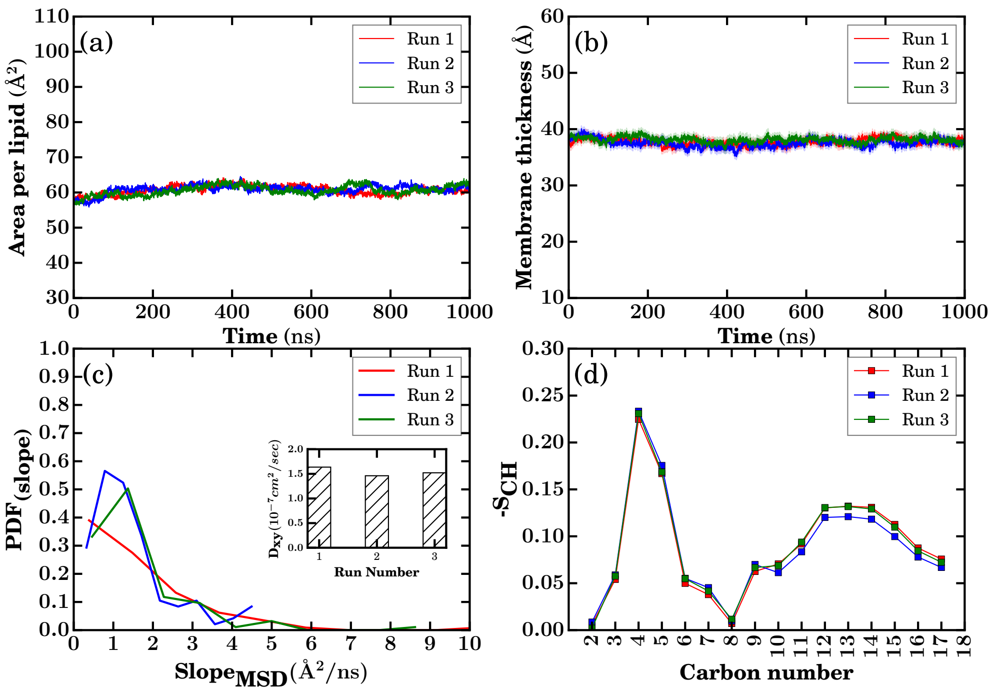

Figure S47: Effect of 2.00 mol% Propanoic acid on membrane properties (a) Area per lipid, (b) Membrane thickness, (c) Distribution of MSD slopes calculated at 10 ns chunks from the MD trajectory with the inset showing the lateral diffusion coefficient (D_{xy}), (d) Deuterium order parameter ($-S_{\mathrm{CH}}$), in an NpT ensemble at 300 K and 1 bar.

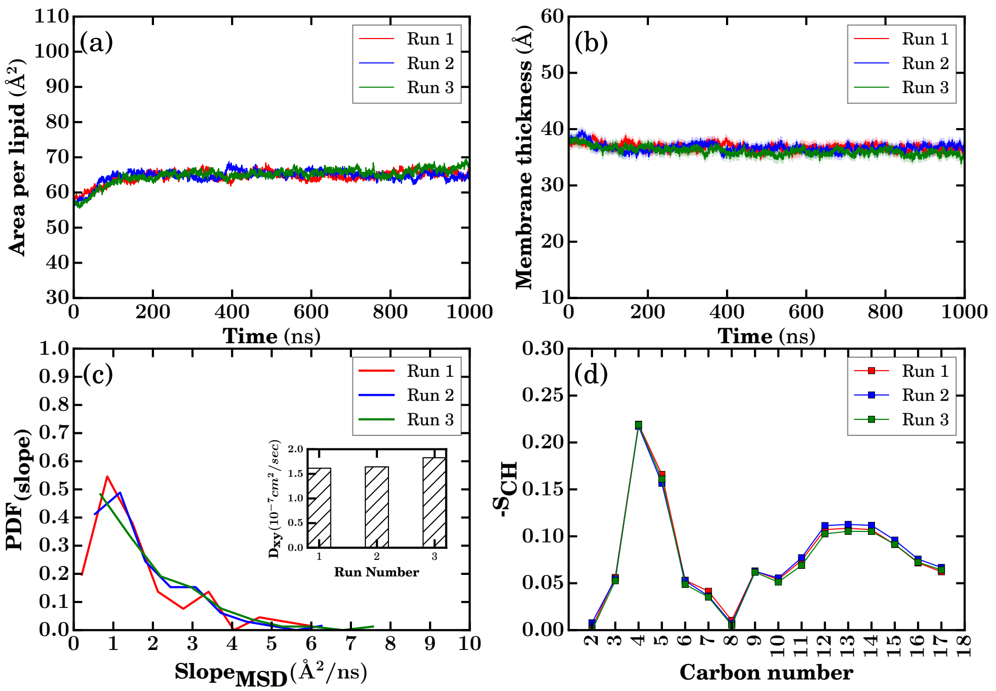

Figure S48: Effect of 2.50 mol% Propanoic acid on membrane properties (a) Area per lipid, (b) Membrane thickness, (c) Distribution of MSD slopes calculated at 10 ns chunks from the MD trajectory with the inset showing the lateral diffusion coefficient (D_{xy}), (d) Deuterium order parameter ($-S_{\mathrm{CH}}$), in an NpT ensemble at 300 K and 1 bar.

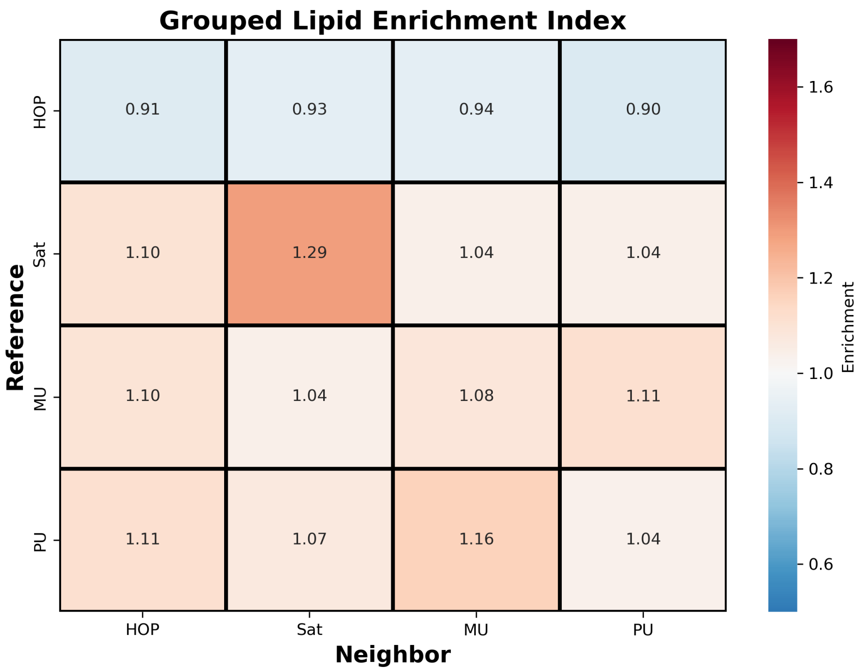

Figure S49: Grouped lipid enrichment indices for hopanoids (HOP) and phospholipid tail classes in *Z. mobilis* control membranes. Rows denote the reference lipid class and columns denote the neighbour class; each matrix element gives the enrichment index of neighbours relative to random mixing (1 = random, > 1 enriched, < 1 depleted). Across both conditions, enrichment values remain close to unity (typically 0.8–1.2), indicating only modest preferences between lipid classes and no clear evidence for strongly segregated hopanoid-rich or hopanoid-poor lateral domains on the simulated length scales.

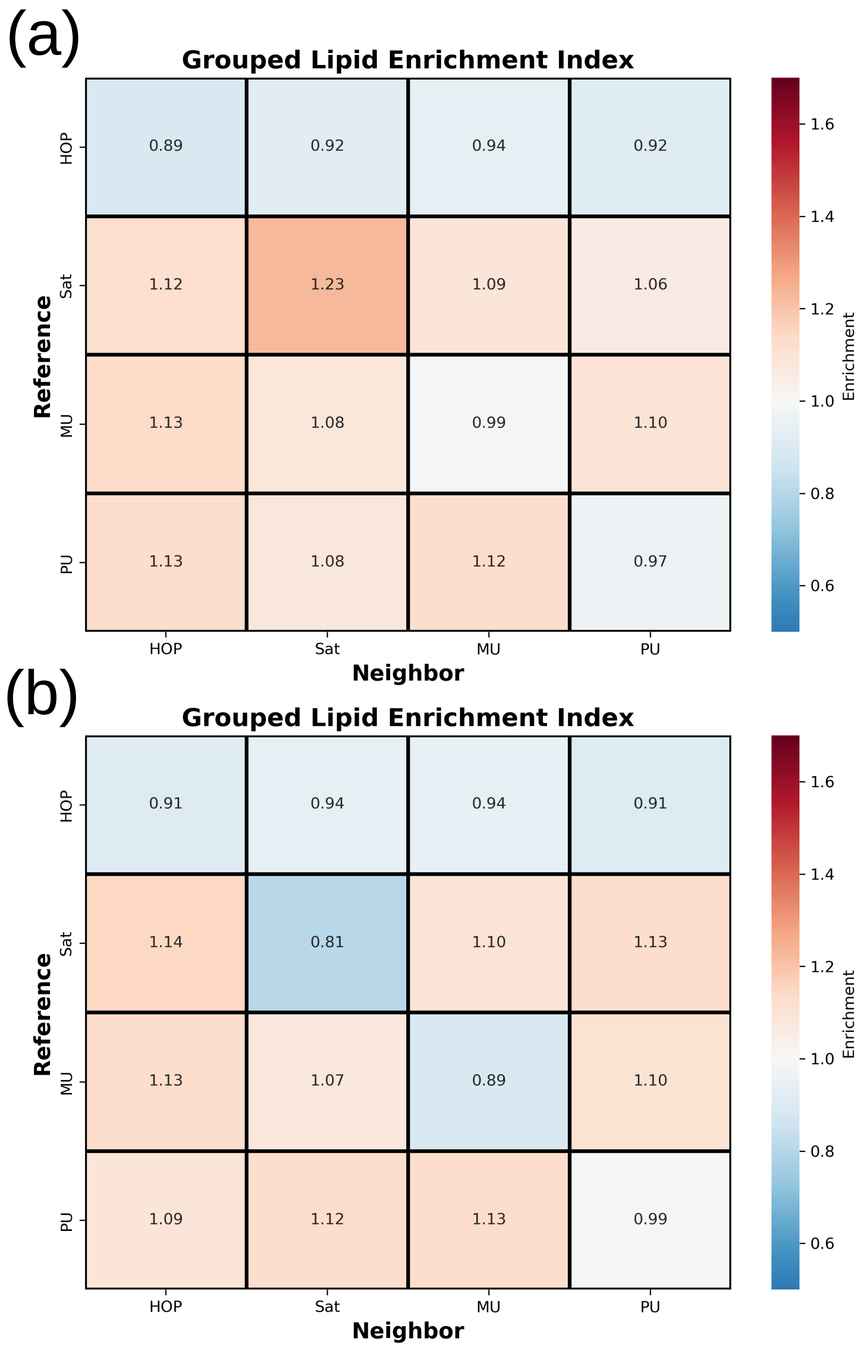

Figure S50: Grouped lipid enrichment indices for hopanoids (HOP) and phospholipid tail classes in *Z. mobilis* membranes. (a) Acetaldehyde at 0.50 mol%. (b) Isobutanol at 2.50 mol%. Rows denote the reference lipid class and columns denote the neighbour class; each matrix element gives the enrichment index of neighbours relative to random mixing (1 = random, > 1 enriched, < 1 depleted). Across both conditions, enrichment values remain close to unity (typically 0.8–1.2), indicating only modest preferences between lipid classes and no clear evidence for strongly segregated hopanoid-rich or hopanoid-poor lateral domains on the simulated length scales.

Molecular distribution and free energy profile across the membrane

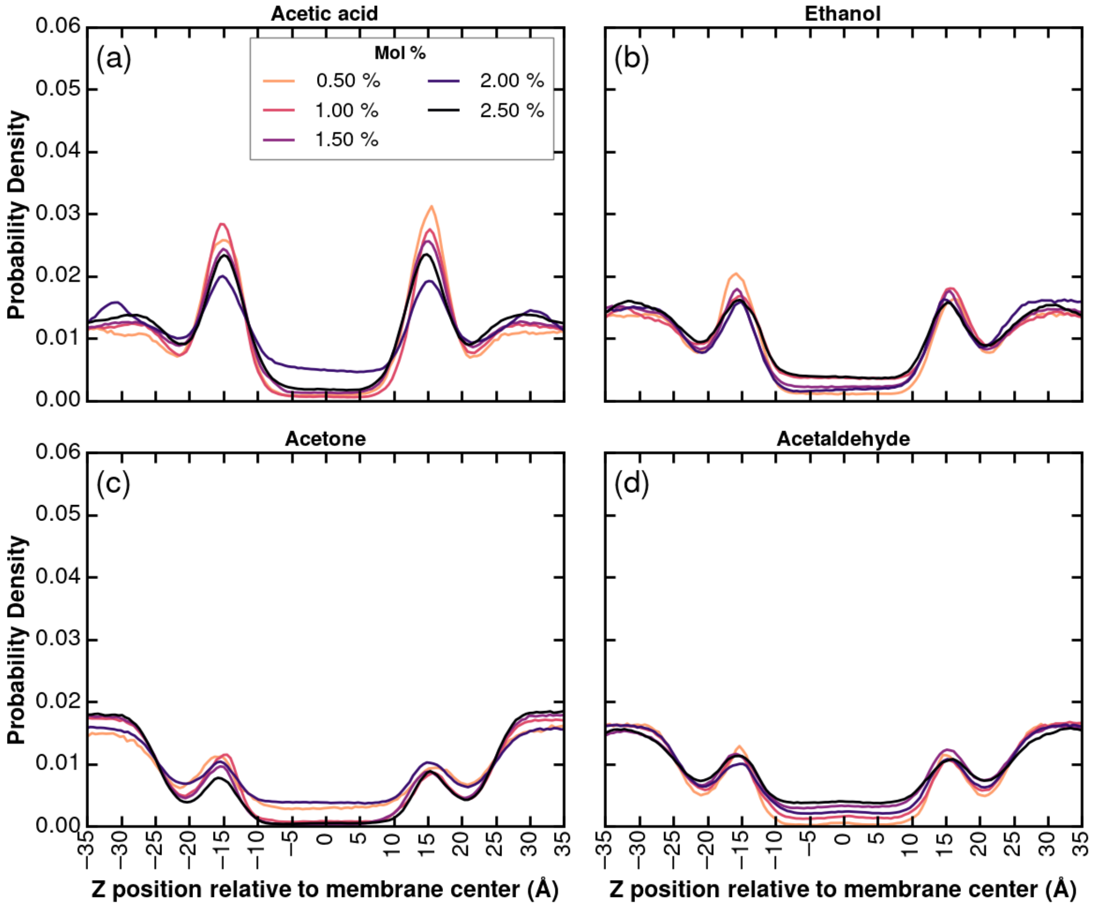

Figure S51: Probability density distributions of (a) acetic acid, (b) ethanol, (c) acetone and (d) acetaldehyde, relative to the membrane center in NpT ensemble at 300 K and 1 bar.

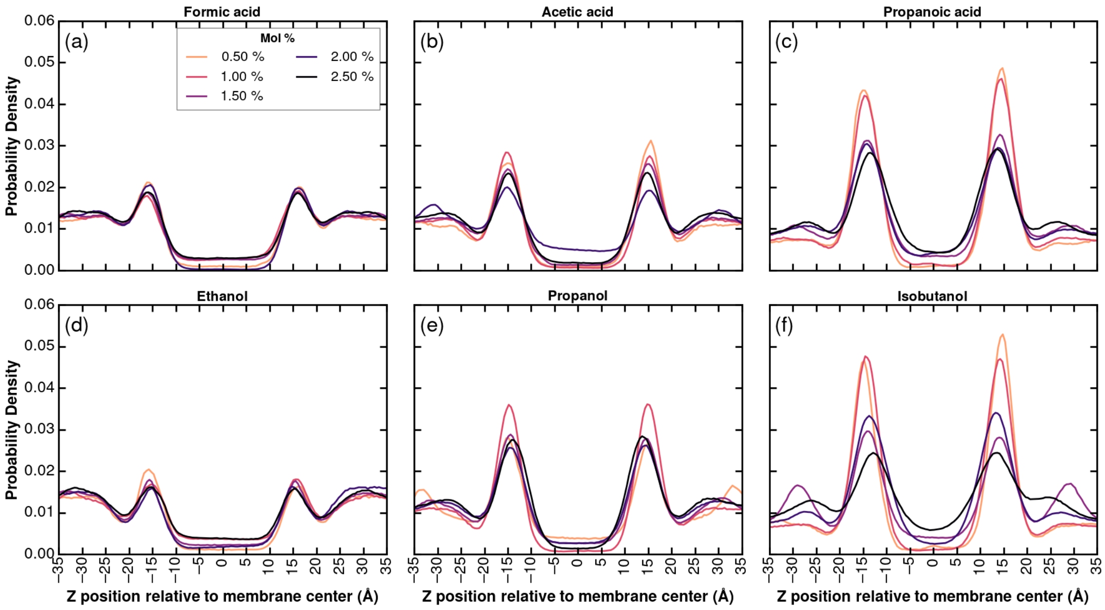

Figure S52: Probability density profiles of carboxylic acids (formic, acetic and propanoic) (a-c) and alcohols (ethanol, propanol and isobutanol)(d-f).

Effect of hopanoids on solvent-stressed membrane properties

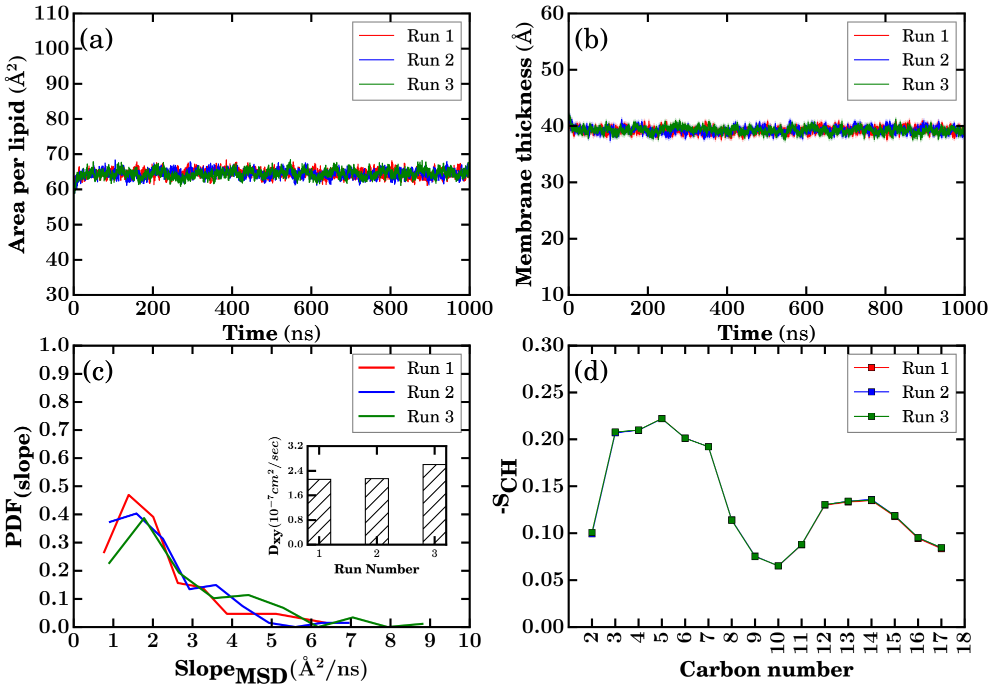

Figure S53: Effect of 0.50 mol% Acetic acid on the membrane properties when no Hopanoids were present in the membrane (a) Area per lipid, (b) Membrane thickness, (c) Distribution of MSD slopes calculated at 10 ns chunks from the MD trajectory with the inset showing the lateral diffusion coefficient (D_{xy}), (d) Deuterium order parameter ($-S_{\mathrm{CH}}$), in an NpT ensemble at 300 K and 1 bar.

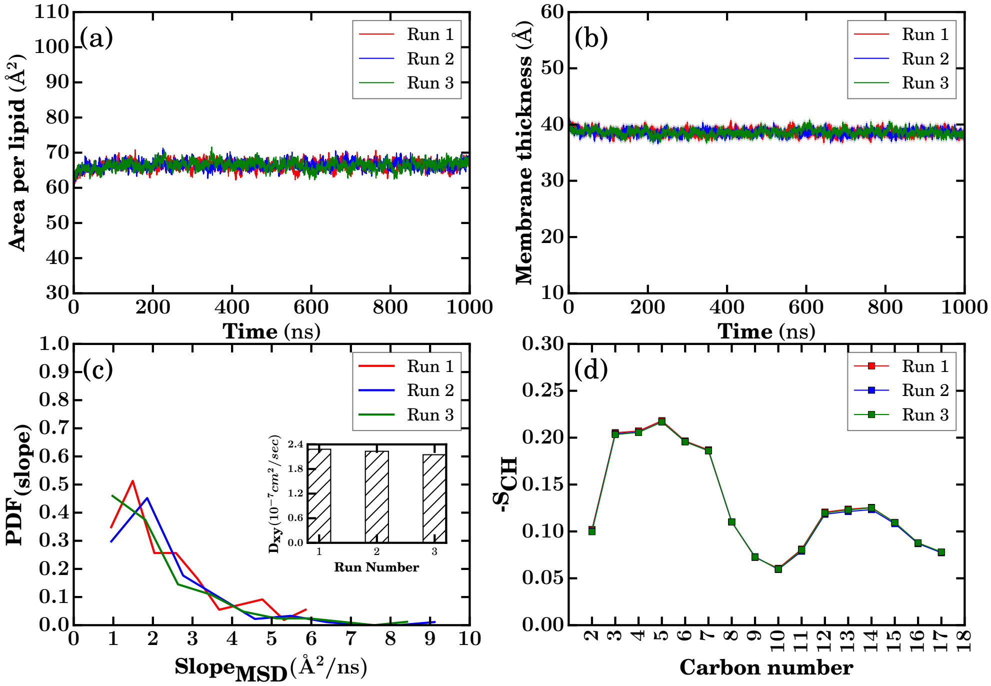

Figure S54: Effect of 1.00 mol% Acetic acid on the membrane properties when no Hopanoids were present in the membrane (a) Area per lipid, (b) Membrane thickness, (c) Distribution of MSD slopes calculated at 10 ns chunks from the MD trajectory with the inset showing the lateral diffusion coefficient (D_{xy}), (d) Deuterium order parameter ($-S_{\mathrm{CH}}$), in an NpT ensemble at 300 K and 1 bar.

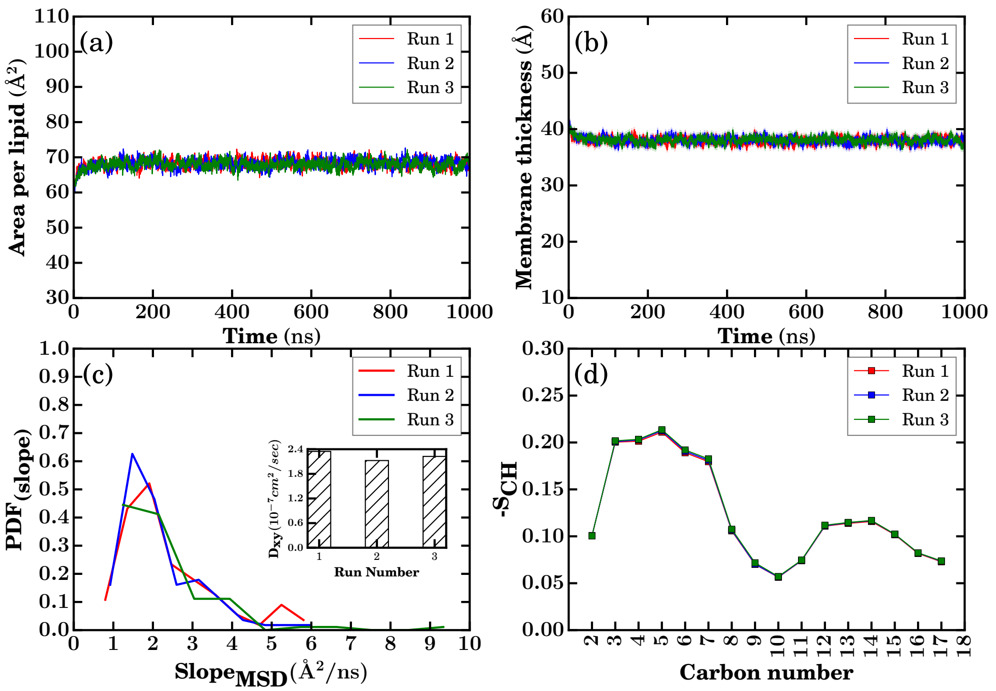

Figure S55: Effect of 1.50 mol% Acetic acid on the membrane properties when no Hopanoids were present in the membrane (a) Area per lipid, (b) Membrane thickness, (c) Distribution of MSD slopes calculated at 10 ns chunks from the MD trajectory with the inset showing the lateral diffusion coefficient (D_{xy}), (d) Deuterium order parameter ($-S_{\mathrm{CH}}$), in an NpT ensemble at 300 K and 1 bar.

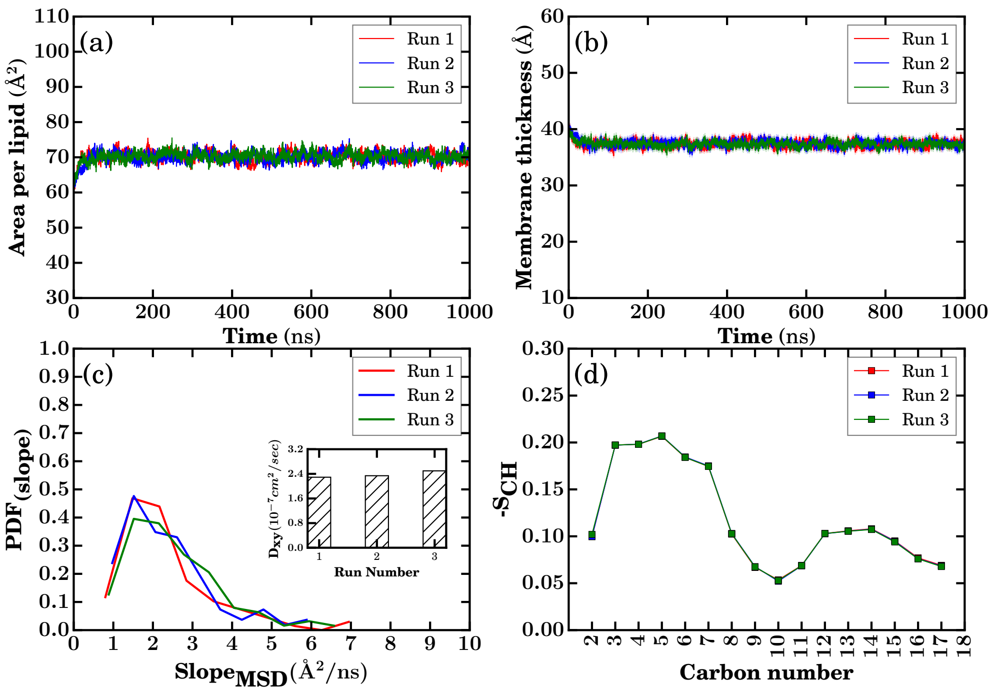

Figure S56: Effect of 2.00 mol% Acetic acid on the membrane properties when no Hopanoids were present in the membrane (a) Area per lipid, (b) Membrane thickness, (c) Distribution of MSD slopes calculated at 10 ns chunks from the MD trajectory with the inset showing the lateral diffusion coefficient (D_{xy}), (d) Deuterium order parameter ($-S_{\mathrm{CH}}$), in an NpT ensemble at 300 K and 1 bar.

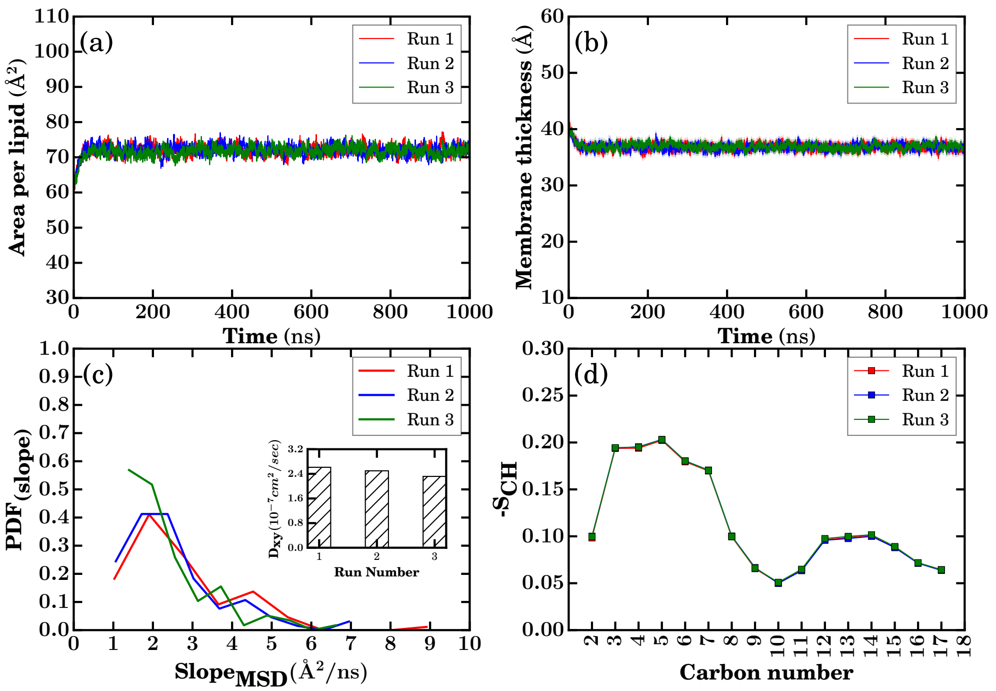

Figure S57: Effect of 2.50 mol% Acetic acid on the membrane properties when no Hopanoids were present in the membrane (a) Area per lipid, (b) Membrane thickness, (c) Distribution of MSD slopes calculated at 10 ns chunks from the MD trajectory with the inset showing the lateral diffusion coefficient (D_{xy}), (d) Deuterium order parameter ($-S_{\mathrm{CH}}$), in an NpT ensemble at 300 K and 1 bar.

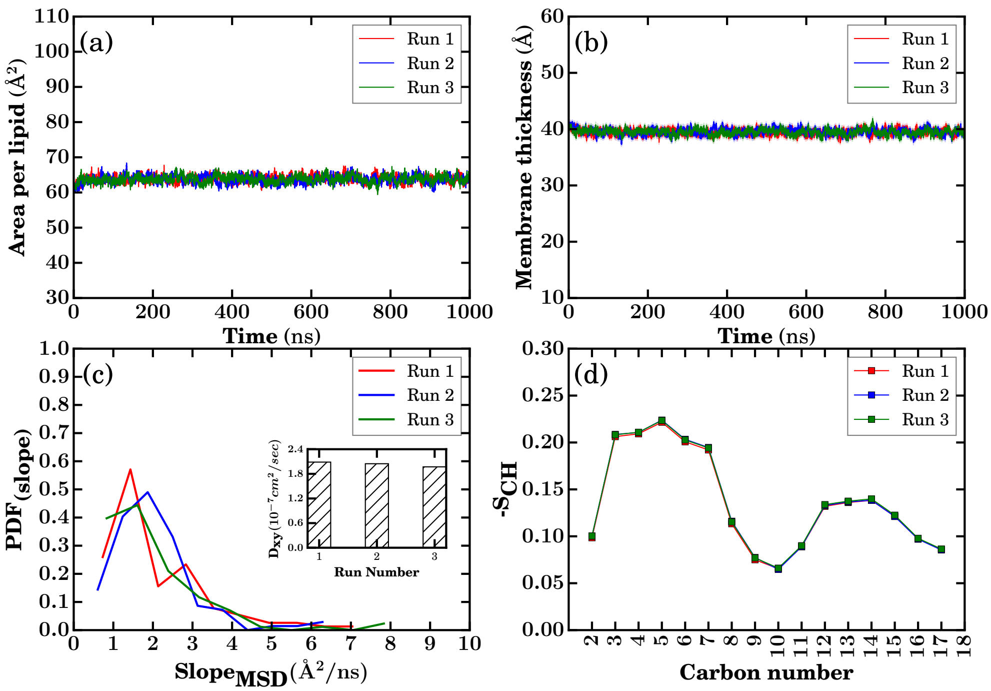

Figure S58: Effect of 0.50 mol% Ethanol on the membrane properties when no Hopanoids were present in the membrane (a) Area per lipid, (b) Membrane thickness, (c) Distribution of MSD slopes calculated at 10 ns chunks from the MD trajectory with the inset showing the lateral diffusion coefficient (D_{xy}), (d) Deuterium order parameter ($-S_{\text{CH}}$), in an NpT ensemble at 300 K and 1 bar.

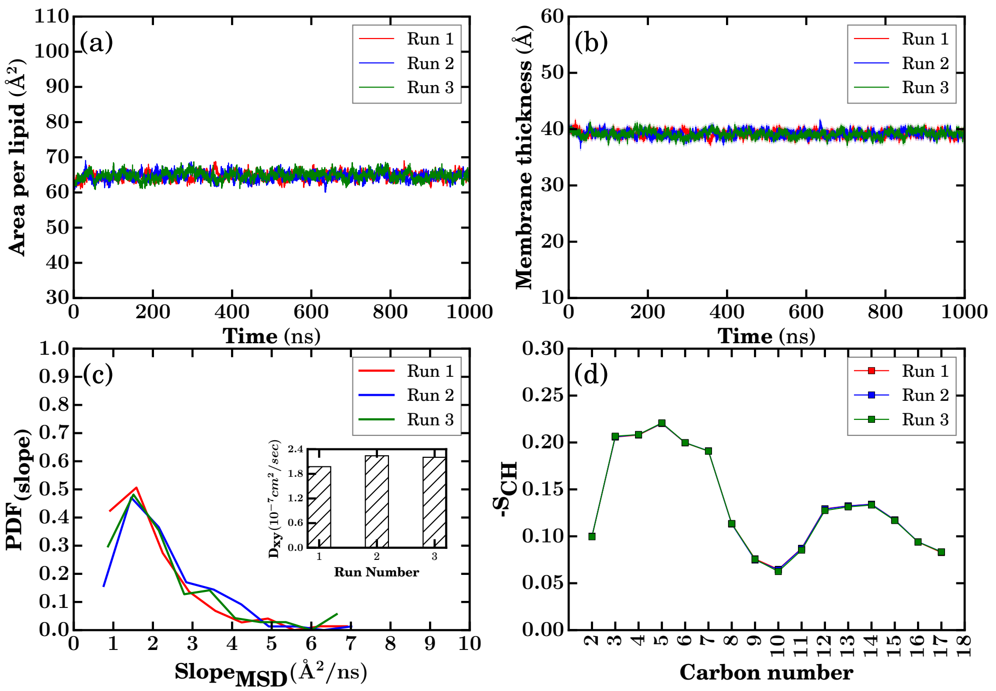

Figure S59: Effect of 1.00 mol% Ethanol on the membrane properties when no Hopanoids were present in the membrane (a) Area per lipid, (b) Membrane thickness, (c) Distribution of MSD slopes calculated at 10 ns chunks from the MD trajectory with the inset showing the lateral diffusion coefficient (D_{xy}), (d) Deuterium order parameter ($-S_{\mathrm{CH}}$), in an NpT ensemble at 300 K and 1 bar.

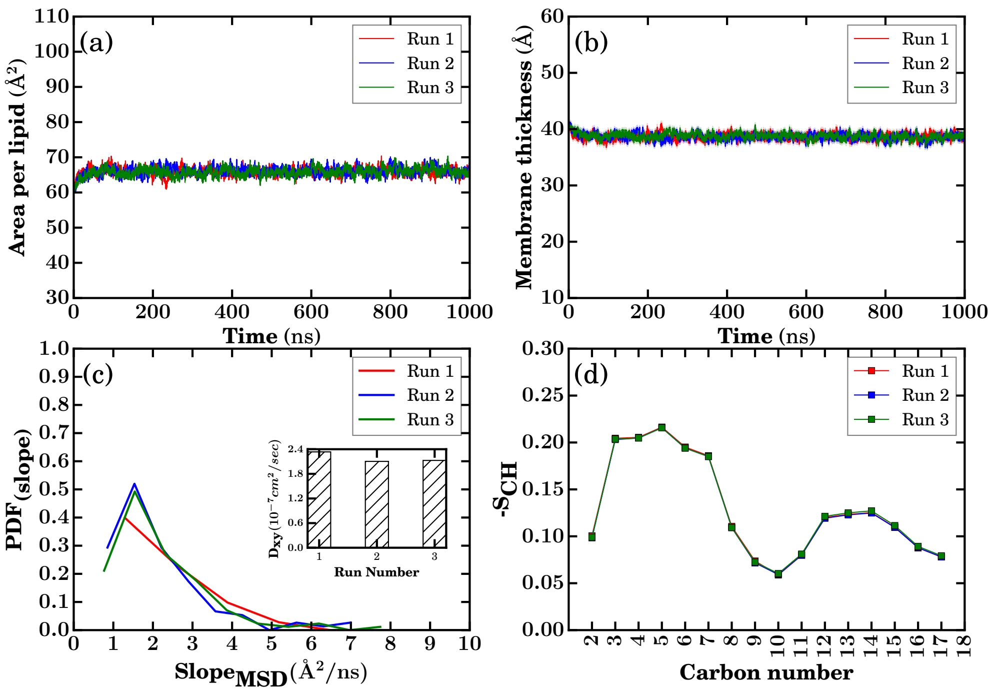

Figure S60: Effect of 1.50 mol% Ethanol on the membrane properties when no Hopanoids were present in the membrane (a) Area per lipid, (b) Membrane thickness, (c) Distribution of MSD slopes calculated at 10 ns chunks from the MD trajectory with the inset showing the lateral diffusion coefficient (D_{xy}), (d) Deuterium order parameter ($-S_{\text{CH}}$), in an NpT ensemble at 300 K and 1 bar.

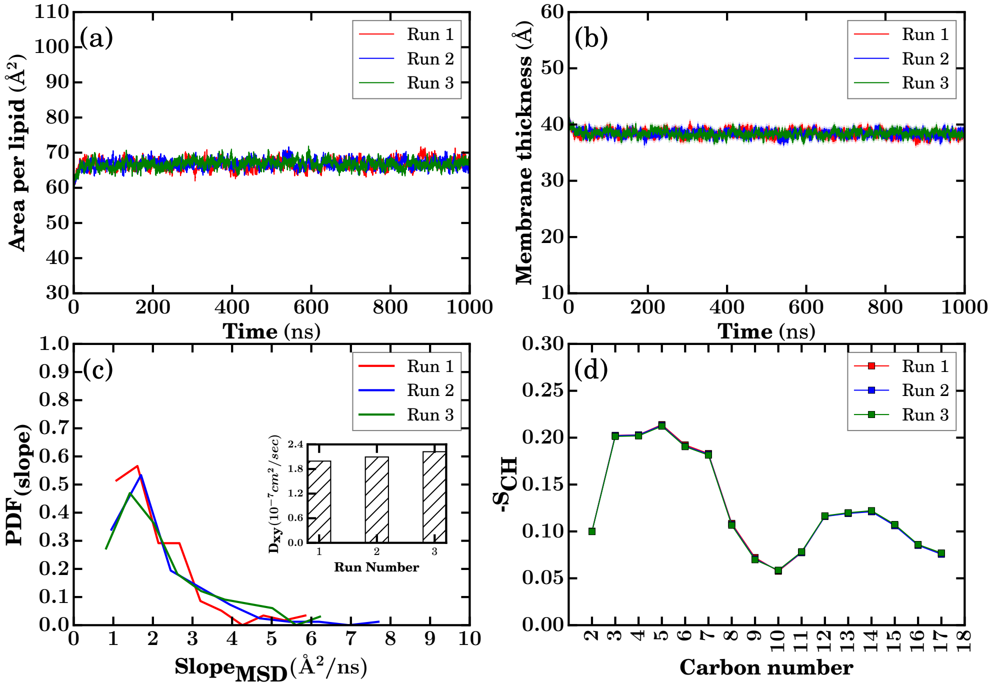

Figure S61: Effect of 2.00 mol% Ethanol on the membrane properties when no Hopanoids were present in the membrane (a) Area per lipid, (b) Membrane thickness, (c) Distribution of MSD slopes calculated at 10 ns chunks from the MD trajectory with the inset showing the lateral diffusion coefficient (D_{xy}), (d) Deuterium order parameter ($-S_{\text{CH}}$), in an NpT ensemble at 300 K and 1 bar.

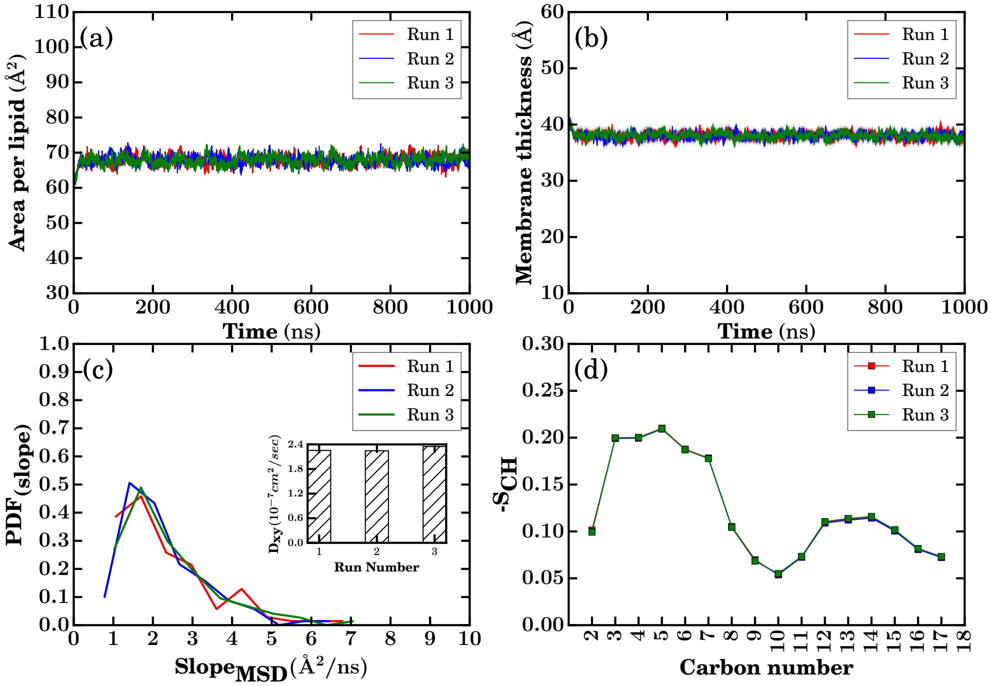

Figure S62: Effect of 2.50 mol% Ethanol on the membrane properties when no Hopanoids were present in the membrane (a) Area per lipid, (b) Membrane thickness, (c) Distribution of MSD slopes calculated at 10 ns chunks from the MD trajectory with the inset showing the lateral diffusion coefficient (D_{xy}), (d) Deuterium order parameter ($-S_{\mathrm{CH}}$), in an NpT ensemble at 300 K and 1 bar.

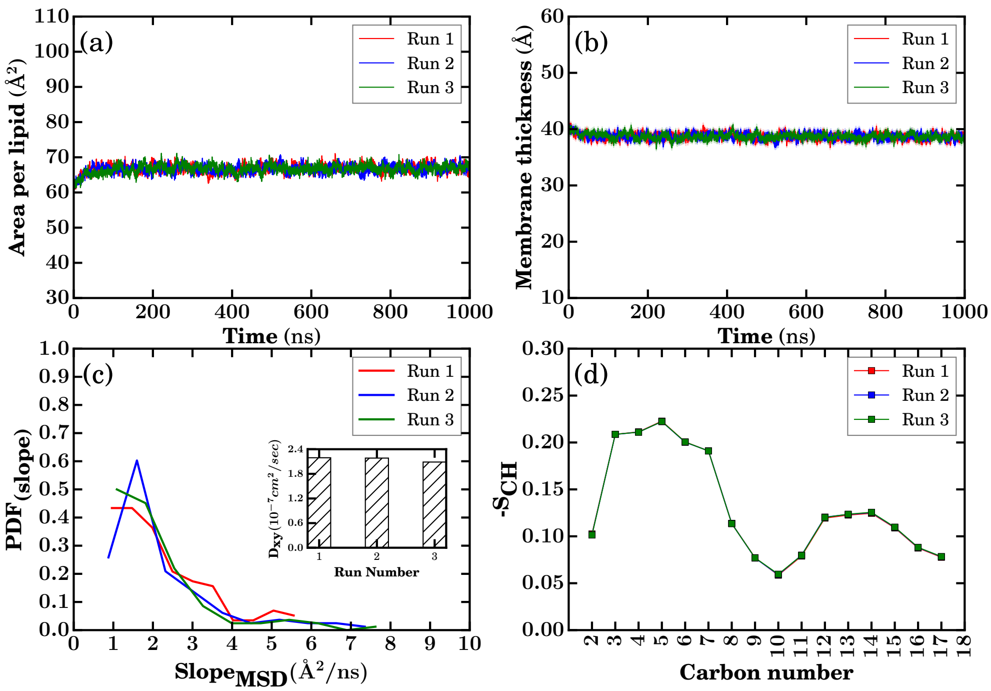

Figure S63: Effect of 0.50 mol% Isobutanol on the membrane properties when no Hopanoids were present in the membrane (a) Area per lipid, (b) Membrane thickness, (c) Distribution of MSD slopes calculated at 10 ns chunks from the MD trajectory with the inset showing the lateral diffusion coefficient (D_{xy}), (d) Deuterium order parameter ($-S_{\mathrm{CH}}$), in an NpT ensemble at 300 K and 1 bar.

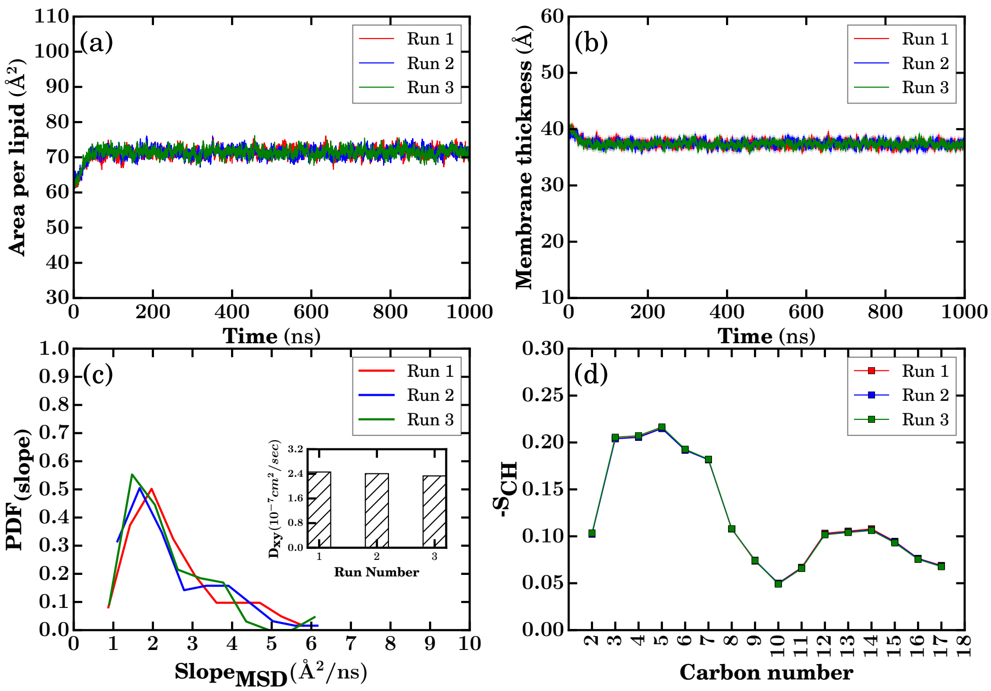

Figure S64: Effect of 1.00 mol% Isobutanol on the membrane properties when no Hopanoids were present in the membrane (a) Area per lipid, (b) Membrane thickness, (c) Distribution of MSD slopes calculated at 10 ns chunks from the MD trajectory with the inset showing the lateral diffusion coefficient (D_{xy}), (d) Deuterium order parameter ($-S_{\mathrm{CH}}$), in an NpT ensemble at 300 K and 1 bar.

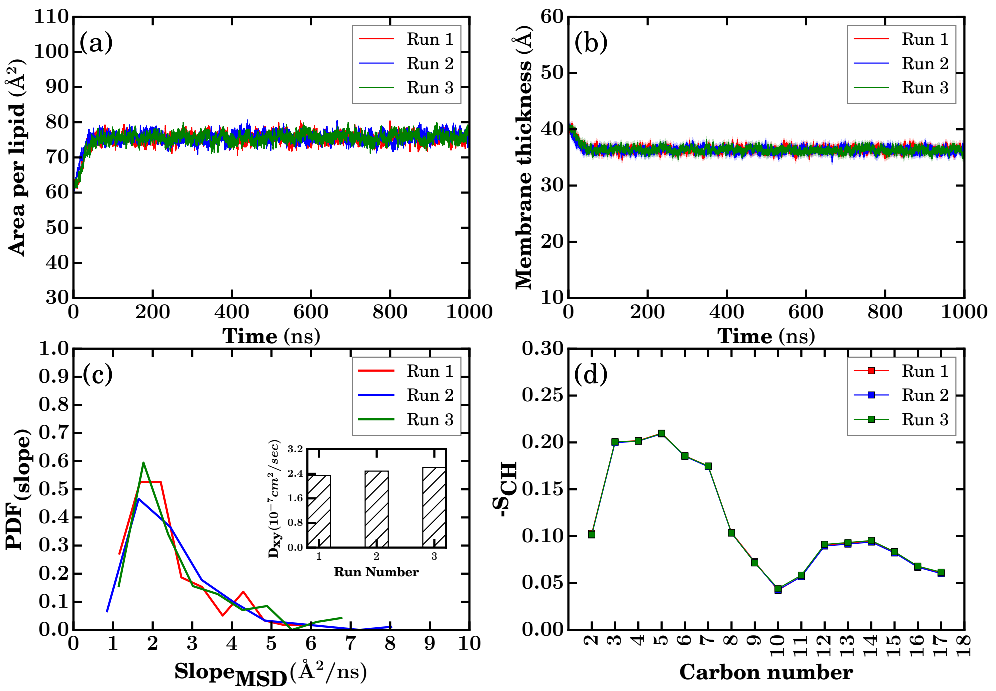

Figure S65: Effect of 1.50 mol% Isobutanol on the membrane properties when no Hopanoids were present in the membrane (a) Area per lipid, (b) Membrane thickness, (c) Distribution of MSD slopes calculated at 10 ns chunks from the MD trajectory with the inset showing the lateral diffusion coefficient (D_{xy}), (d) Deuterium order parameter ($-S_{\mathrm{CH}}$), in an NpT ensemble at 300 K and 1 bar.

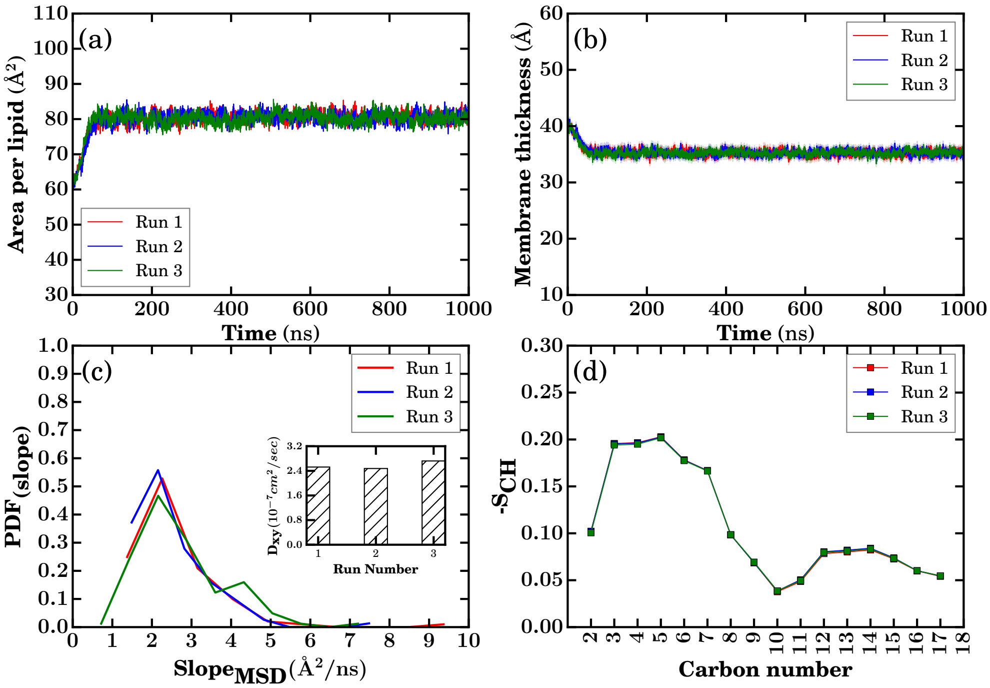

Figure S66: Effect of 2.00 mol% Isobutanol on the membrane properties when no Hopanoids were present in the membrane (a) Area per lipid, (b) Membrane thickness, (c) Distribution of MSD slopes calculated at 10 ns chunks from the MD trajectory with the inset showing the lateral diffusion coefficient (D_{xy}), (d) Deuterium order parameter ($-S_{\mathrm{CH}}$), in an NpT ensemble at 300 K and 1 bar.

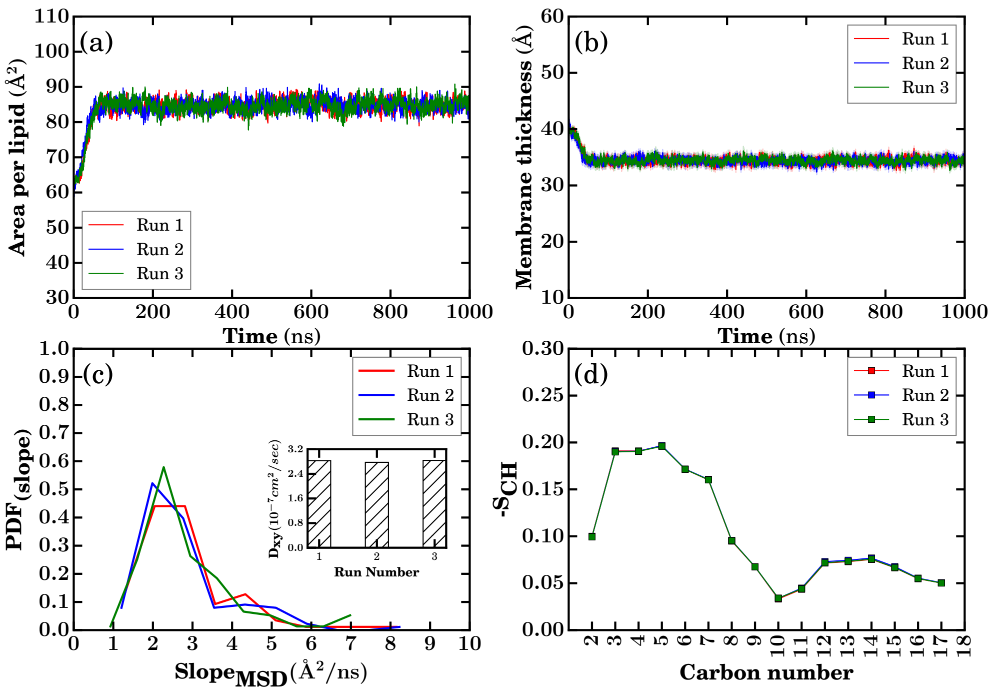

Figure S67: Effect of 2.50 mol% Isobutanol on the membrane properties when no Hopanoids were present in the membrane (a) Area per lipid, (b) Membrane thickness, (c) Distribution of MSD slopes calculated at 10 ns chunks from the MD trajectory with the inset showing the lateral diffusion coefficient (D_{xy}), (d) Deuterium order parameter ($-S_{\mathrm{CH}}$), in an NpT ensemble at 300 K and 1 bar.

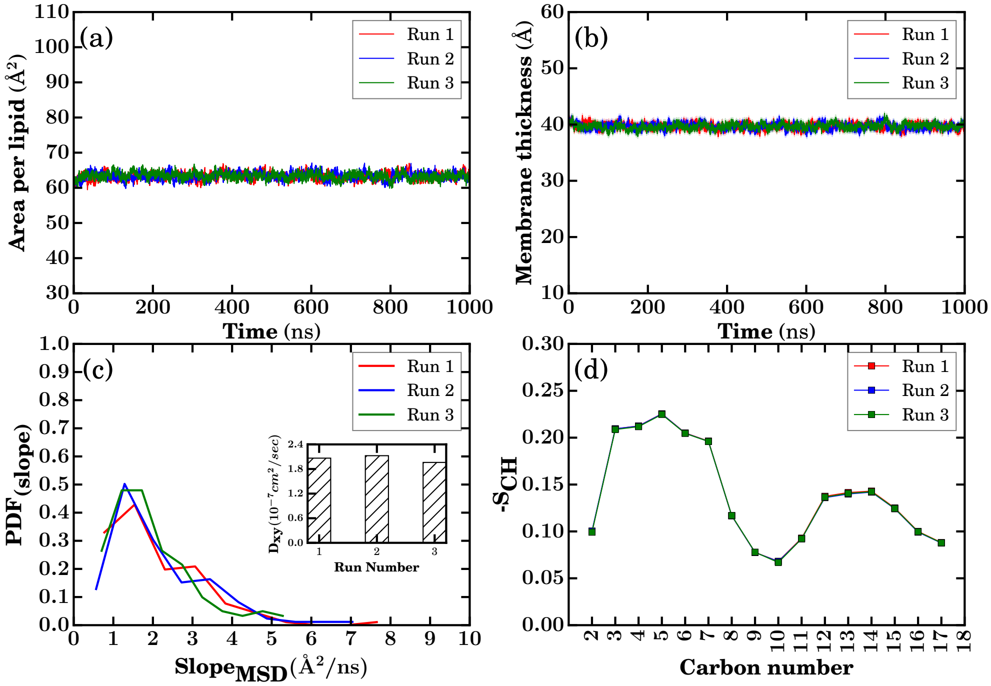

Figure S68: Effect of 0.50 mol% Formic acid on the membrane properties when no Hopanoids were present in the membrane (a) Area per lipid, (b) Membrane thickness, (c) Distribution of MSD slopes calculated at 10 ns chunks from the MD trajectory with the inset showing the lateral diffusion coefficient (D_{xy}), (d) Deuterium order parameter ($-S_{\mathrm{CH}}$), in an NpT ensemble at 300 K and 1 bar.

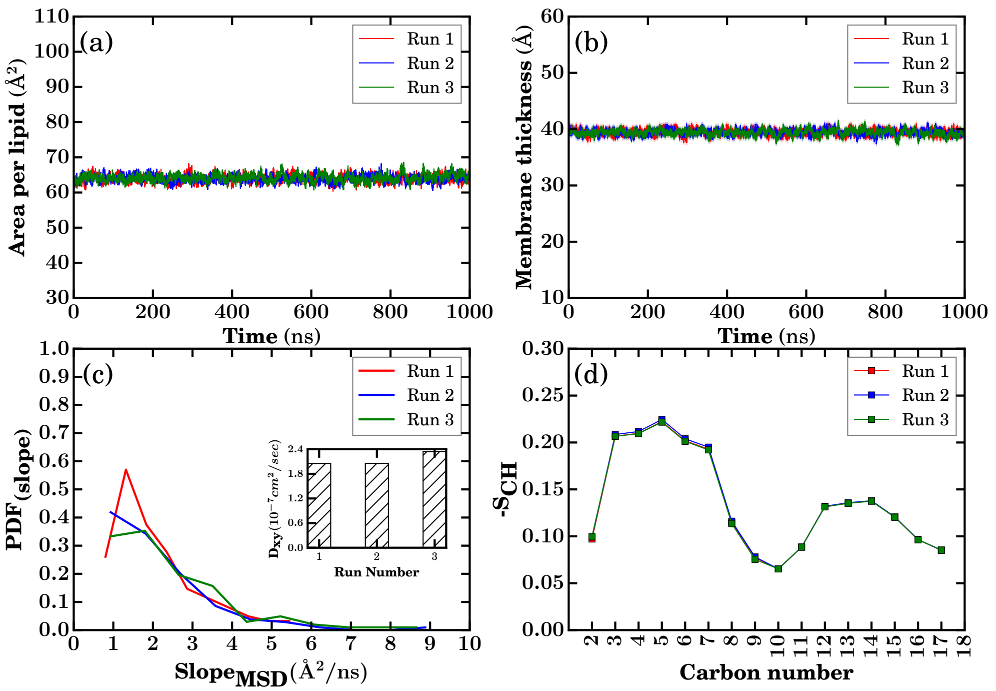

Figure S69: Effect of 1.00 mol% Formic acid on the membrane properties when no Hopanoids were present in the membrane (a) Area per lipid, (b) Membrane thickness, (c) Distribution of MSD slopes calculated at 10 ns chunks from the MD trajectory with the inset showing the lateral diffusion coefficient (D_{xy}), (d) Deuterium order parameter ($-S_{\mathrm{CH}}$), in an NpT ensemble at 300 K and 1 bar.

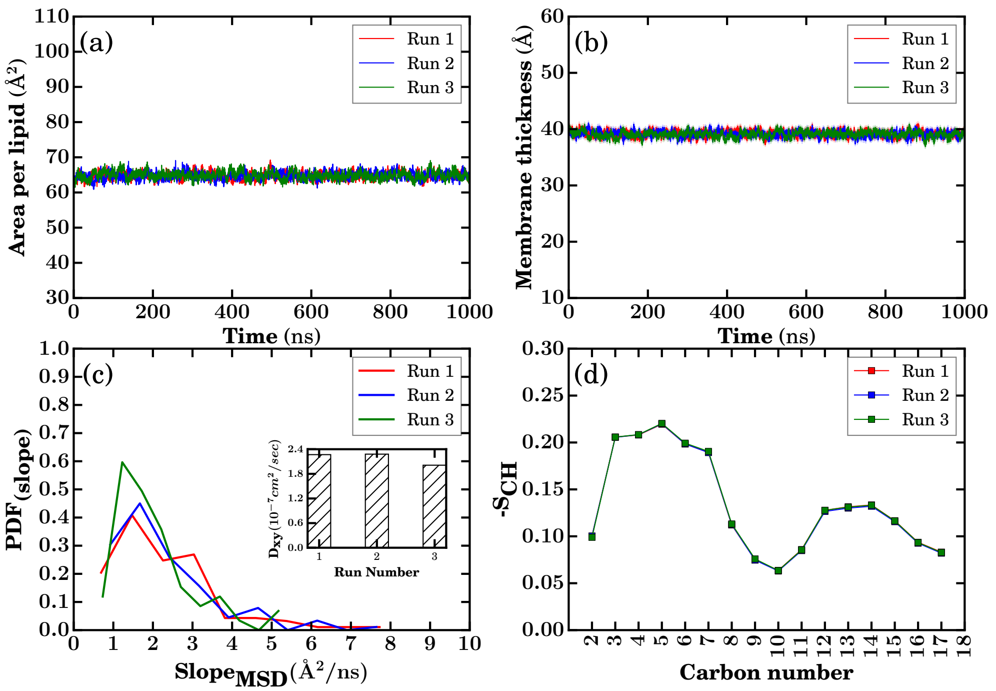

Figure S70: Effect of 1.50 mol% Formic acid on the membrane properties when no Hopanoids were present in the membrane (a) Area per lipid, (b) Membrane thickness, (c) Distribution of MSD slopes calculated at 10 ns chunks from the MD trajectory with the inset showing the lateral diffusion coefficient (D_{xy}), (d) Deuterium order parameter ($-S_{\mathrm{CH}}$), in an NpT ensemble at 300 K and 1 bar.

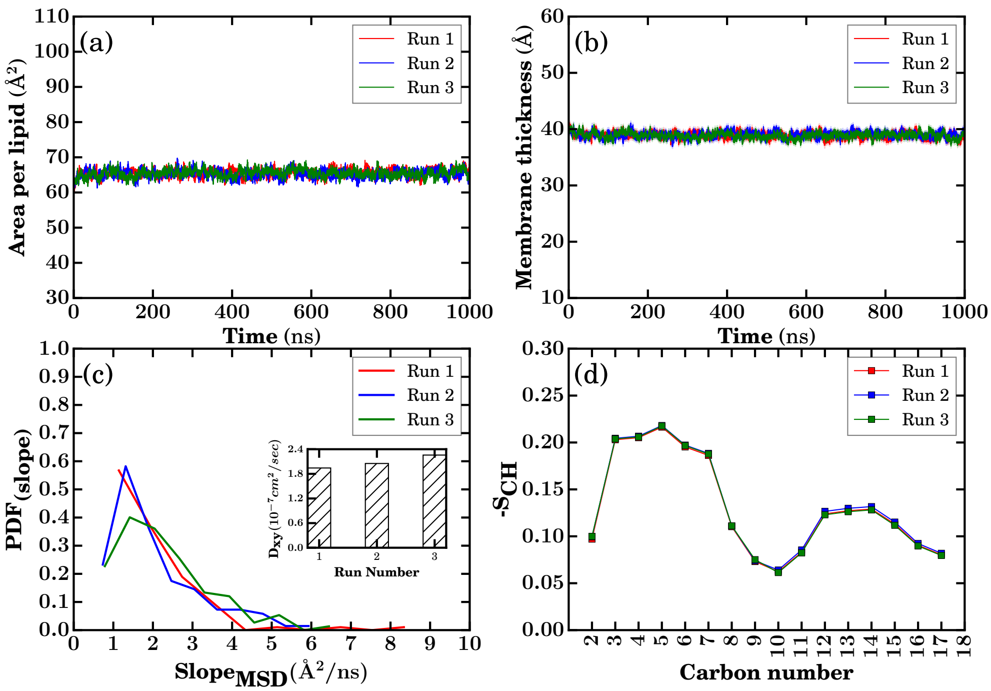

Figure S71: Effect of 2.00 mol% Formic acid on the membrane properties when no Hopanoids were present in the membrane (a) Area per lipid, (b) Membrane thickness, (c) Distribution of MSD slopes calculated at 10 ns chunks from the MD trajectory with the inset showing the lateral diffusion coefficient (D_{xy}), (d) Deuterium order parameter ($-S_{\text{CH}}$), in an NpT ensemble at 300 K and 1 bar.

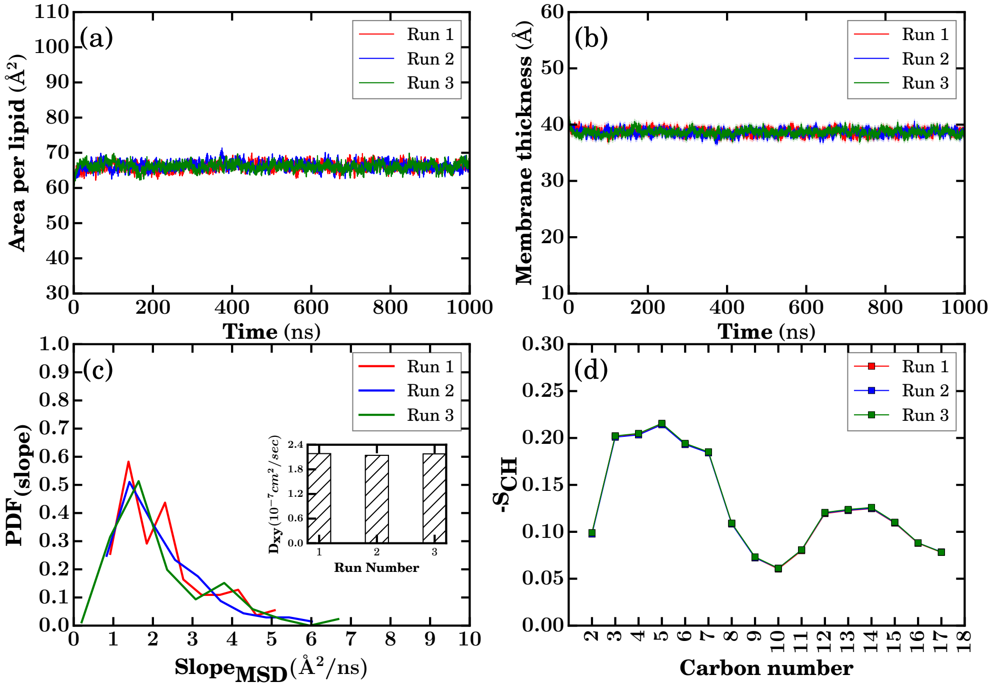

Figure S72: Effect of 2.50 mol% Formic acid on the membrane properties when no Hopanoids were present in the membrane (a) Area per lipid, (b) Membrane thickness, (c) Distribution of MSD slopes calculated at 10 ns chunks from the MD trajectory with the inset showing the lateral diffusion coefficient (D_{xy}), (d) Deuterium order parameter ($-S_{\mathrm{CH}}$), in an NpT ensemble at 300 K and 1 bar.

Membrane-crossing and permeabilities of small molecules

For counting the membrane crossing events of small molecules, we unwrapped the membrane and solute molecules using VMD's `fastpbc` commands, ensuring that all positions were continuous across the box boundaries. The scripts compute the z-coordinates of each solute relative to the instantaneous membrane center in each frame, effectively centering the membrane at $z = 0$. For each molecule, we track its z-position over time and identify crossing events relative to the membrane midplane, taking the PBC-corrected coordinates into account. This ensures that apparent translocations due to molecules crossing the periodic boundary are not miscounted as actual translocation events. Furthermore, the analysis computes the average membrane height or area to normalize the translocation frequency, providing a consistent measure of permeability across the different replicates and membrane systems. Membrane crossing events for all molecules across different concentrations and runs are shown in Table S2.

Molecule	Mol %	Run 1	Run 2	Run 3	Total	Mean ± Std
Acetic acid	0.50	4	1	3	8	2.67 ± 1.25
	1.00	2	3	2	7	2.33 ± 0.47
	1.50	9	11	3	23	7.67 ± 3.40
	2.00	10	8	10	28	9.33 ± 0.94
	2.50	30	11	32	73	24.33 ± 9.46
Ethanol	0.50	1	0	0	1	0.33 ± 0.47
	1.00	5	1	0	6	2.00 ± 2.16
	1.50	2	0	2	4	1.33 ± 0.94
	2.00	2	3	6	11	3.67 ± 1.70

Continued on next page

Molecule	Mol %	Run 1	Run 2	Run 3	Total	Mean ± Std
	2.50	6	5	4	15	5.00 ± 0.82
Furfural	0.50	13	3	1	17	5.67 ± 5.25
	1.00	10	6	17	33	11.00 ± 4.55
	1.50	38	26	25	89	29.67 ± 5.91
	2.00	36	29	39	104	34.67 ± 4.19
	2.50	63	75	79	217	72.33 ± 6.80
Acetone	0.50	1	2	0	3	1.00 ± 0.82
	1.00	0	1	0	1	0.33 ± 0.47
	1.50	1	1	1	3	1.00 ± 0.00
	2.00	3	2	3	8	2.67 ± 0.47
	2.50	3	3	2	8	2.67 ± 0.47
Acetaldehyde	0.50	4	4	7	15	5.00 ± 1.41
	1.00	14	10	13	37	12.33 ± 1.70
	1.50	16	20	12	48	16.00 ± 3.27
	2.00	37	39	38	114	38.00 ± 0.82
	2.50	27	33	37	97	32.33 ± 4.11
HMF	0.50	0	0	0	0	0.00 ± 0.00
	1.00	0	0	0	0	0.00 ± 0.00
	1.50	0	0	0	0	0.00 ± 0.00
	2.00	0	0	0	0	0.00 ± 0.00
	2.50	0	0	0	0	0.00 ± 0.00
Isobutanol	0.50	3	2	1	6	2.00 ± 0.82
	1.00	17	11	10	38	12.67 ± 3.09
	1.50	36	34	20	90	30.00 ± 7.12
	2.00	77	95	82	254	84.67 ± 7.59
	2.50	126	162	155	443	147.67 ± 15.58
Formic acid	0.50	1	4	3	8	2.67 ± 1.25
	1.00	4	8	9	21	7.00 ± 2.16
	1.50	12	13	15	40	13.33 ± 1.25
	2.00	22	18	24	64	21.33 ± 2.49
	2.50	23	40	33	96	32.00 ± 6.98
Propanoic acid	0.50	4	1	3	8	2.67 ± 1.25
	1.00	14	16	11	41	13.67 ± 2.05
	1.50	46	29	33	108	36.00 ± 7.26
	2.00	66	97	85	248	82.67 ± 12.76
	2.50	124	130	163	417	139.00 ± 17.15

Table S2: Membrane crossing events for all molecules across different concentrations and runs. Values shown for three independent runs with mean and standard deviation.

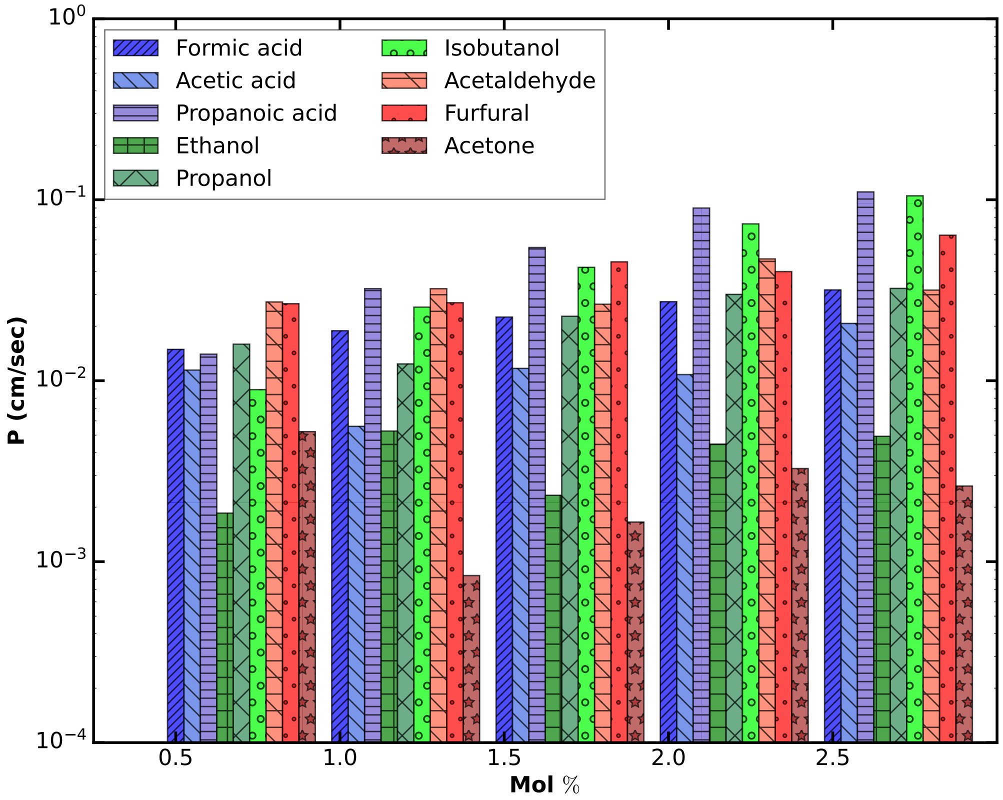

Figure S73: Permeability values of different compounds at five different concentration range (0.5 mol% to 2.5 mol%) calculated by counting the complete leaflet-to-leaflet crossings.

Molecule	Mol %	Crossings	log P (cm/s)
Formic acid	0.5	8	-1.83
	1.0	21	-1.72
	1.5	40	-1.65
	2.0	64	-1.56
	2.5	96	-1.50
Acetic acid	0.5	8	-1.94
	1.0	7	-2.25
	1.5	23	-1.93
	2.0	28	-1.97
	2.5	73	-1.68
Propanoic acid	0.5	8	-1.85
	1.0	41	-1.49
	1.5	108	-1.26
	2.0	248	-1.05
	2.5	417	-0.96
Ethanol	0.5	1	-2.73
	1.0	6	-2.28
	1.5	4	-2.63
	2.0	11	-2.35
	2.5	15	-2.31
Propanol	0.5	9	-1.80

	1.0	15	-1.91
	1.5	43	-1.64
	2.0	80	-1.52
	2.5	101	-1.49
Isobutanol	0.5	6	-2.05
	1.0	38	-1.59
	1.5	90	-1.37
	2.0	254	-1.13
	2.5	443	-0.98
Acetaldehyde	0.5	15	-1.57
	1.0	37	-1.49
	1.5	48	-1.58
	2.0	114	-1.33
	2.5	97	-1.50
Acetone	0.5	3	-2.28
	1.0	1	-3.08
	1.5	3	-2.78
	2.0	8	-2.49
	2.5	8	-2.58
Furfural	0.5	17	-1.57
	1.0	33	-1.57
	1.5	89	-1.34
	2.0	104	-1.40
	2.5	217	-1.20
HMF	0.5	0	Not defined
	1.0	0	Not defined
	1.5	0	Not defined
	2.0	0	Not defined
	2.5	0	Not defined

Table S3: Number of membrane-crossing events and calculated permeability coefficients ($\log P$ (cm/s)) for different small molecules at five bulk mole fractions. Translocation events are counted over a period of 1000 ns for three independent runs.

Free energy profile for solute translocation across the membrane

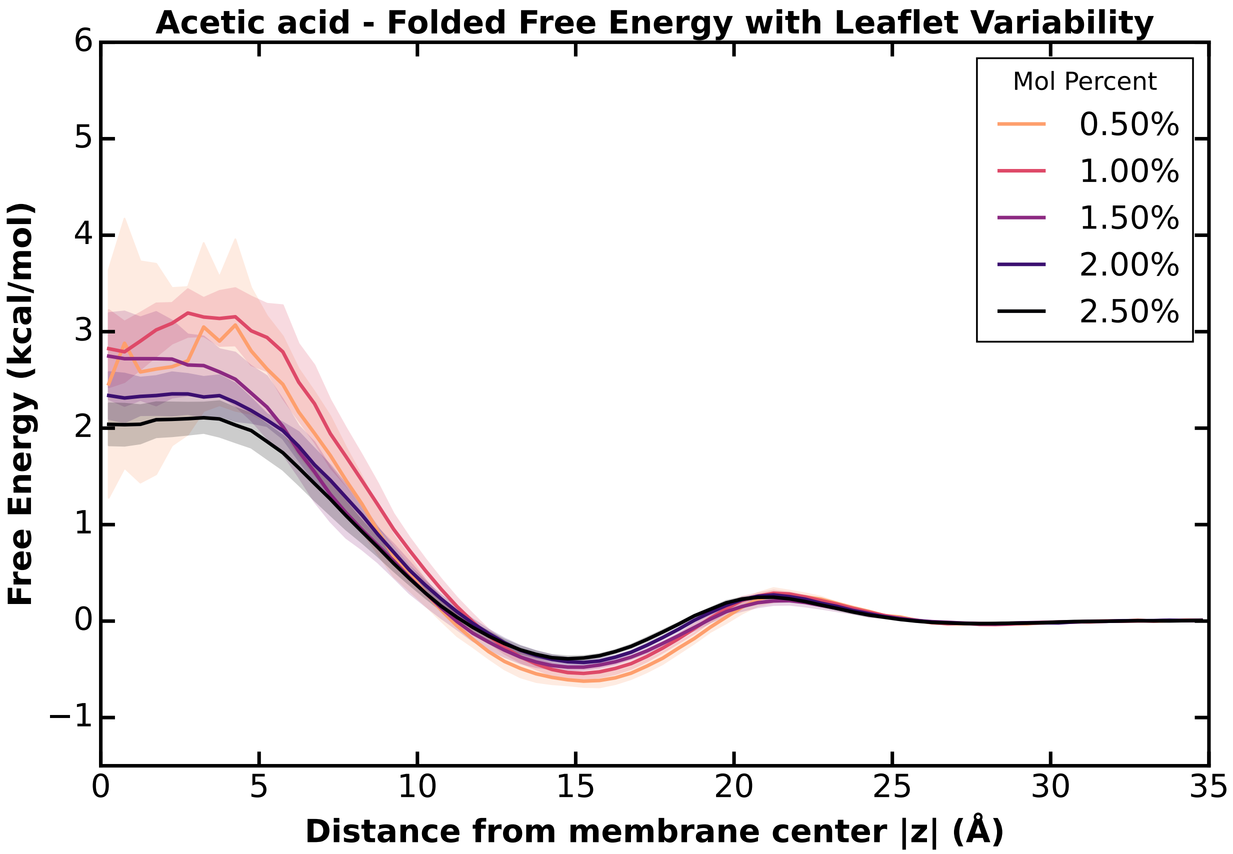

Figure S74: Free-energy profiles for solute translocation of Acetic acid are shown as a function of distance from the membrane center, $|z|$, after folding individual leaflet data about the bilayer midplane. Solid lines represent the mean free-energy profile averaged over six independent leaflets (three simulations $\times$ two leaflets), while shaded regions indicate standard deviation across leaflets. Results are shown for concentrations ranging from 0.5 to 2.5 mol%.

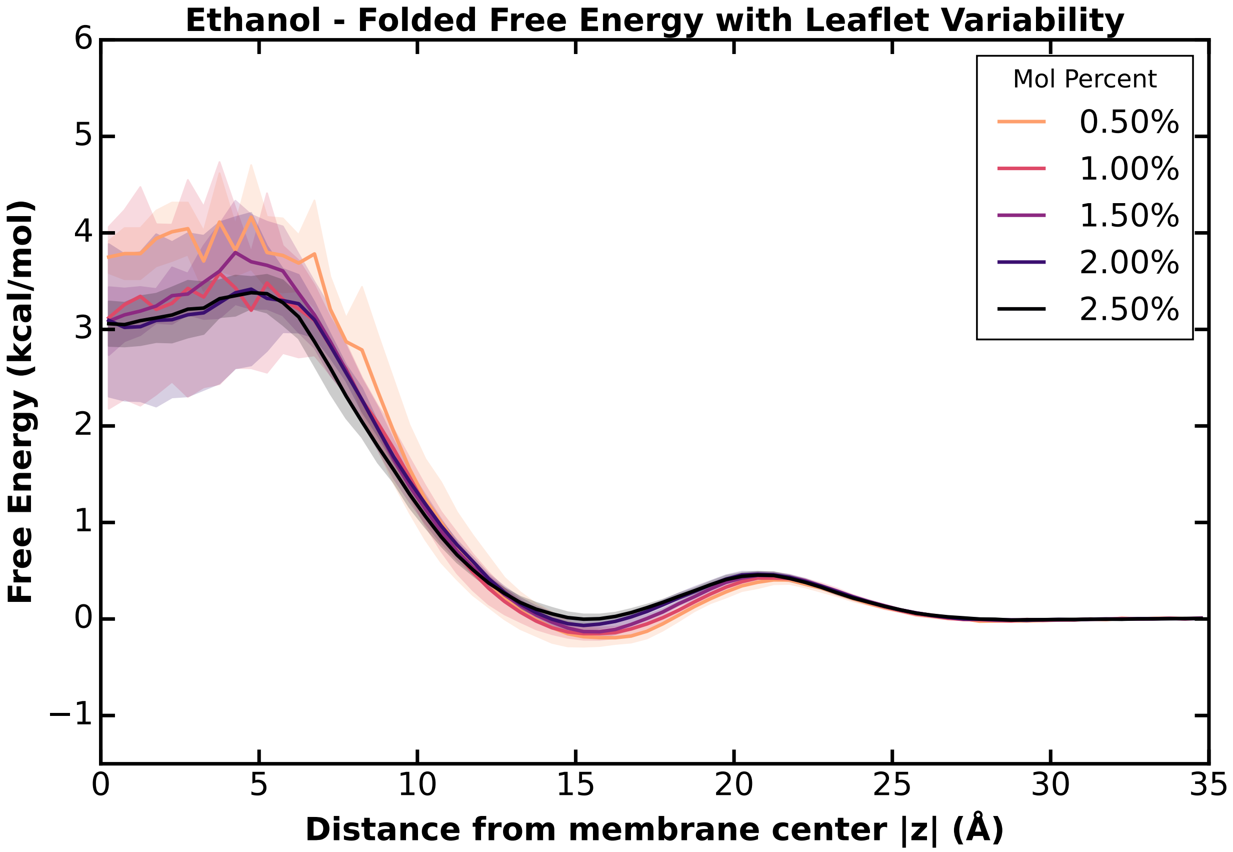

Figure S75: Free-energy profiles for solute translocation of Ethanol are shown as a function of distance from the membrane center, $|z|$, after folding individual leaflet data about the bilayer midplane. Solid lines represent the mean free-energy profile averaged over six independent leaflets (three simulations $\times$ two leaflets), while shaded regions indicate standard deviation across leaflets. Results are shown for concentrations ranging from 0.5 to 2.5 mol%.

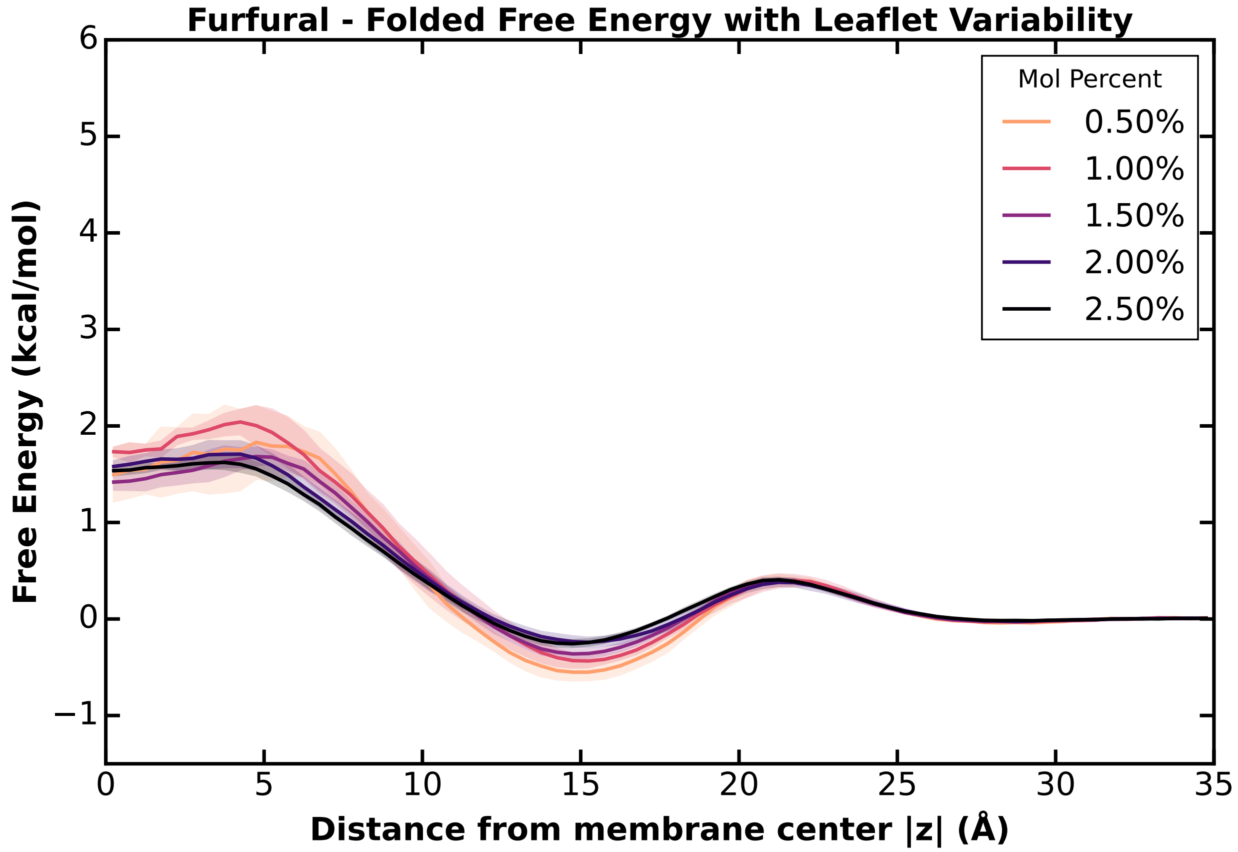

Figure S76: Free-energy profiles for solute translocation of Furfural are shown as a function of distance from the membrane center, $|z|$, after folding individual leaflet data about the bilayer midplane. Solid lines represent the mean free-energy profile averaged over six independent leaflets (three simulations × two leaflets), while shaded regions indicate standard deviation across leaflets. Results are shown for concentrations ranging from 0.5 to 2.5 mol%.

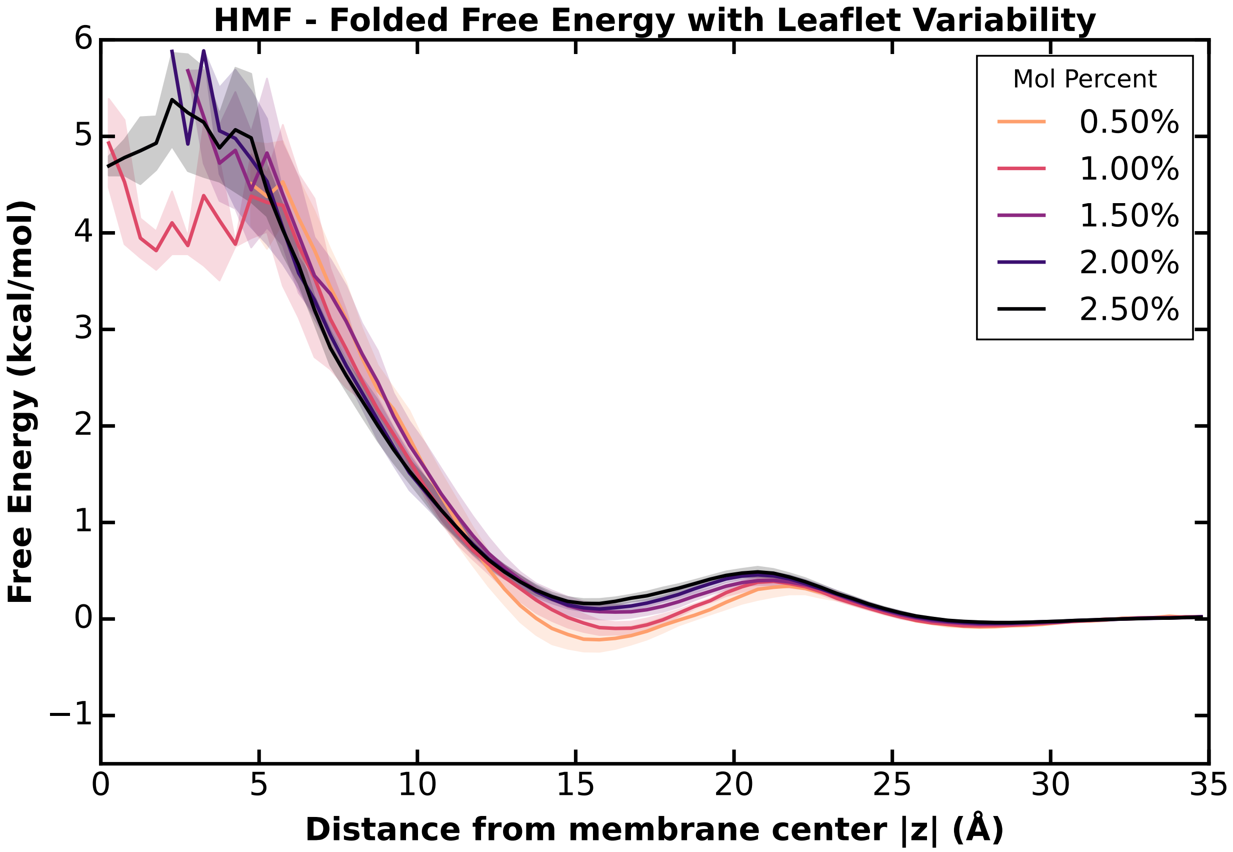

Figure S77: Free-energy profiles for solute translocation of HMF are shown as a function of distance from the membrane center, $|z|$, after folding individual leaflet data about the bilayer midplane. Solid lines represent the mean free-energy profile averaged over six independent leaflets (three simulations $\times$ two leaflets), while shaded regions indicate standard deviation across leaflets. Results are shown for concentrations ranging from 0.5 to 2.5 mol%.

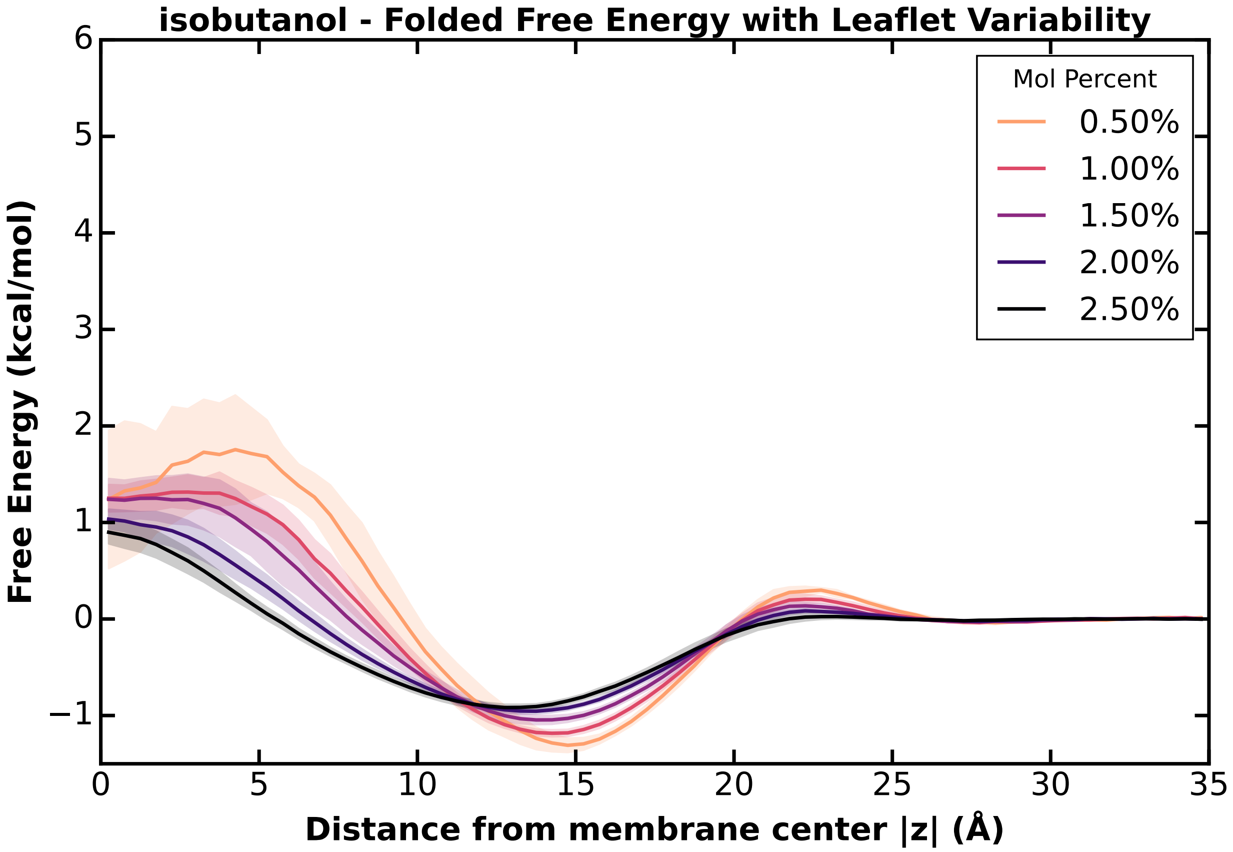

Figure S78: Free-energy profiles for solute translocation of Isobutanol are shown as a function of distance from the membrane center, $|z|$, after folding individual leaflet data about the bilayer midplane. Solid lines represent the mean free-energy profile averaged over six independent leaflets (three simulations × two leaflets), while shaded regions indicate standard deviation across leaflets. Results are shown for concentrations ranging from 0.5 to 2.5 mol%.

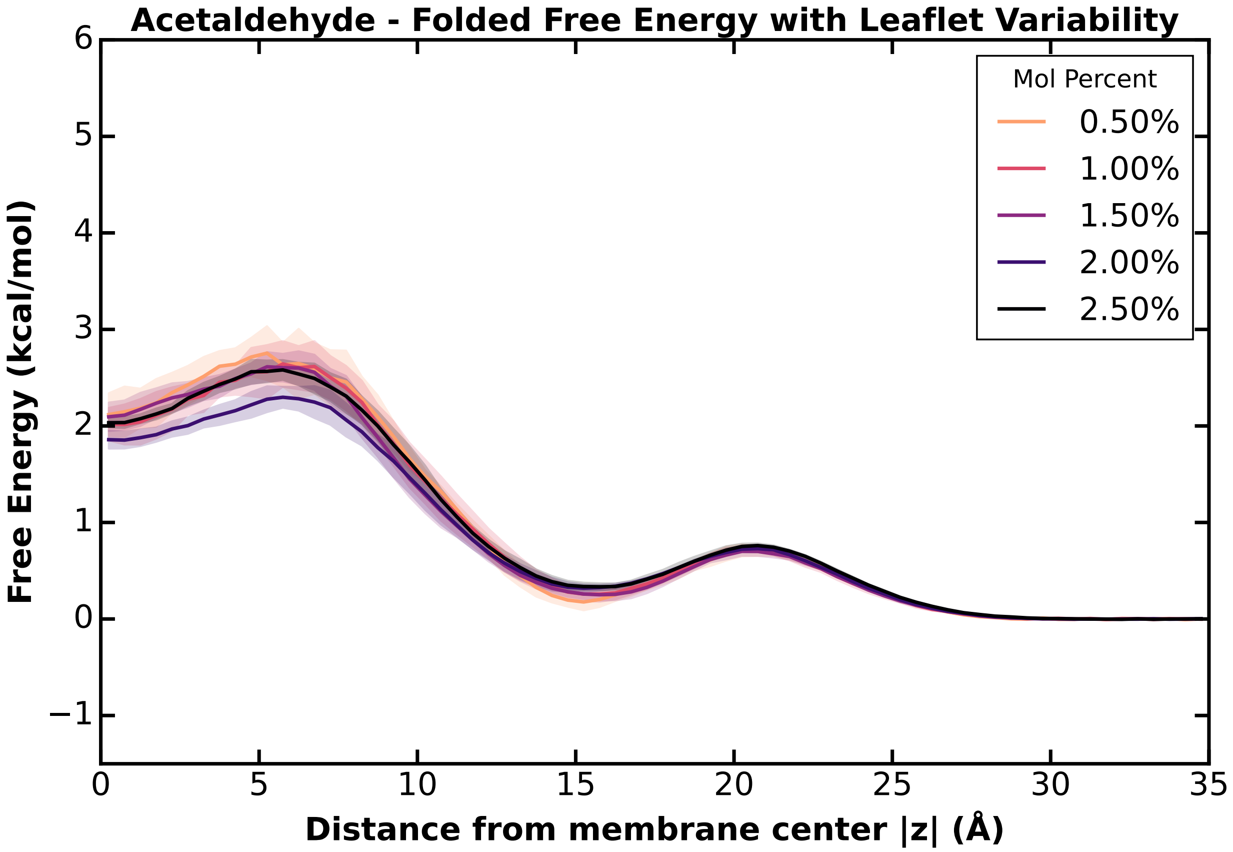

Figure S79: Free-energy profiles for solute translocation of Acetaldehyde are shown as a function of distance from the membrane center, $|z|$, after folding individual leaflet data about the bilayer midplane. Solid lines represent the mean free-energy profile averaged over six independent leaflets (three simulations $\times$ two leaflets), while shaded regions indicate standard deviation across leaflets. Results are shown for concentrations ranging from 0.5 to 2.5 mol%.

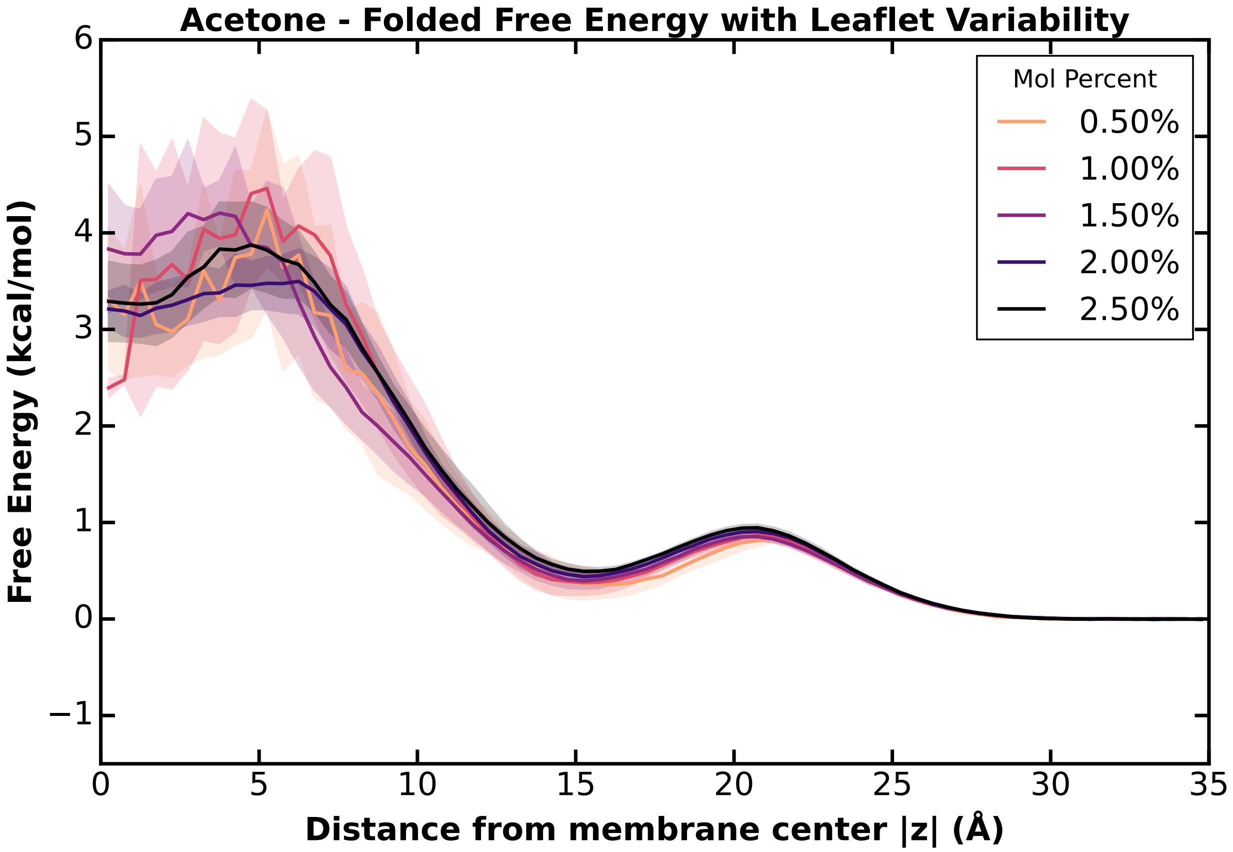

Figure S80: Free-energy profiles for solute translocation of Acetone are shown as a function of distance from the membrane center, $|z|$, after folding individual leaflet data about the bilayer midplane. Solid lines represent the mean free-energy profile averaged over six independent leaflets (three simulations $\times$ two leaflets), while shaded regions indicate standard deviation across leaflets. Results are shown for concentrations ranging from 0.5 to 2.5 mol%.

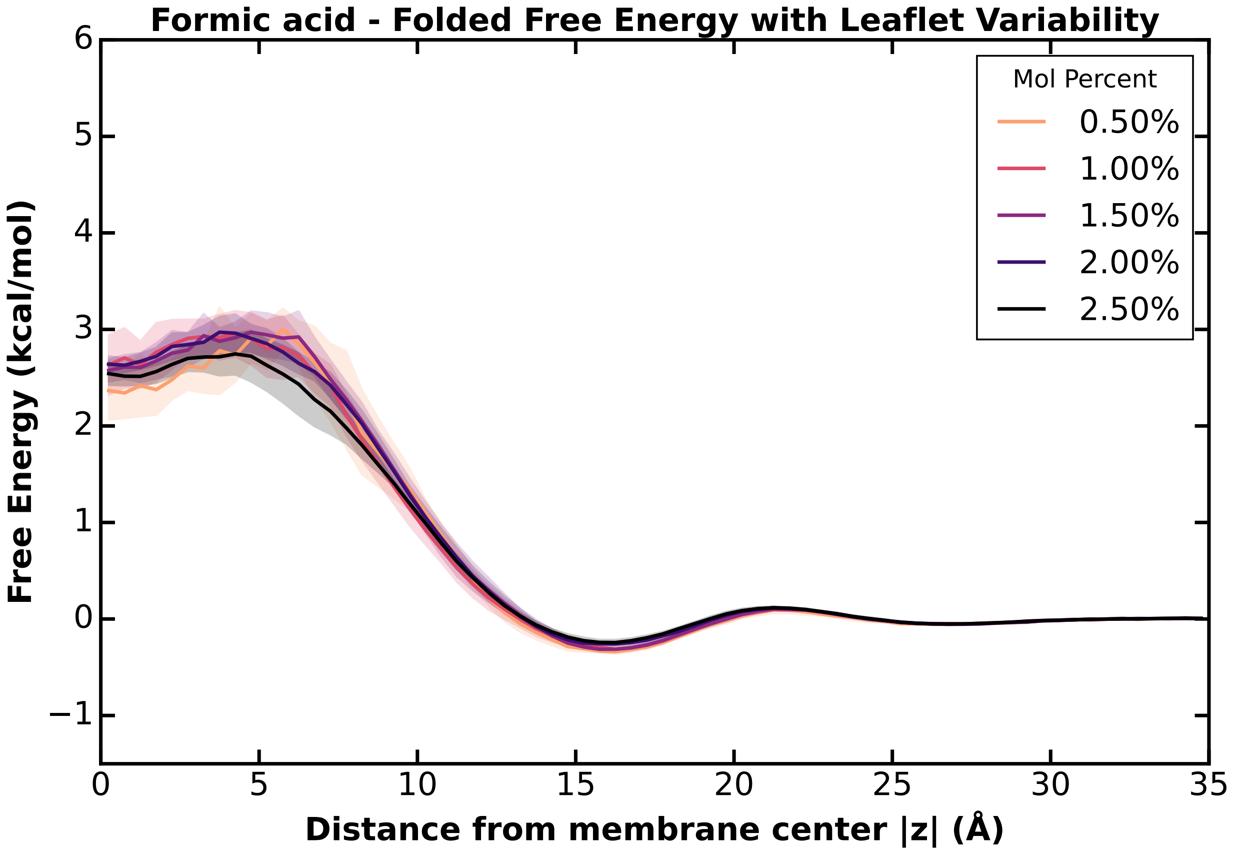

Figure S81: Free-energy profiles for solute translocation of Formic acid are shown as a function of distance from the membrane center, $|z|$, after folding individual leaflet data about the bilayer midplane. Solid lines represent the mean free-energy profile averaged over six independent leaflets (three simulations $\times$ two leaflets), while shaded regions indicate standard deviation across leaflets. Results are shown for concentrations ranging from 0.5 to 2.5 mol%.

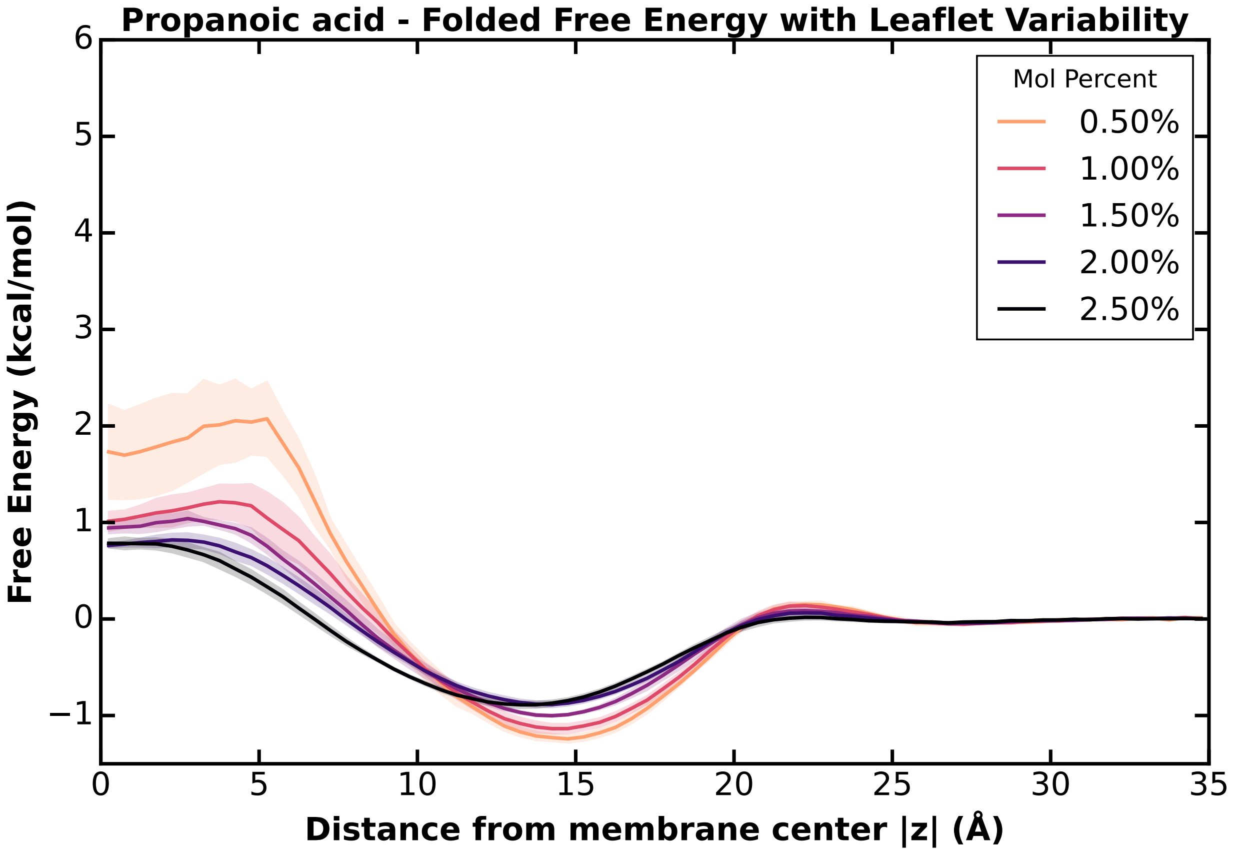

Figure S82: Free-energy profiles for solute translocation of Propanoic acid are shown as a function of distance from the membrane center, $|z|$, after folding individual leaflet data about the bilayer midplane. Solid lines represent the mean free-energy profile averaged over six independent leaflets (three simulations $\times$ two leaflets), while shaded regions indicate standard deviation across leaflets. Results are shown for concentrations ranging from 0.5 to 2.5 mol%.

Effect of hopanoids on solvent-stressed membrane properties.

Table S4: Area per lipid (Å^2) at two inhibitor concentrations with and without the hopanoid in membrane. The Area per lipid (Å^2) at 0 % solute concentration is reported in Table S1.

	With hopanoids		No hopanoids	
Inhibitor	0.5 mol%	2.5 mol%	0.5 mol%	2.5 mol%
Formic acid	53.39 ± 0.81	54.39 ± 0.30	63.29 ± 0.04	66.21 ± 0.12
Acetic acid	53.04 ± 0.24	56.94 ± 0.56	64.48 ± 0.11	71.98 ± 0.04
Ethanol	52.99 ± 0.48	54.93 ± 0.43	63.82 ± 0.06	68.02 ± 0.13
Isobutanol	54.47 ± 0.44	69.48 ± 0.02	66.81 ± 0.04	84.74 ± 0.09

Table S5: Membrane thickness (Å) at two inhibitor concentrations with and without the hopanoid in membrane. The Membrane thickness (Å) at 0 % solute concentration is reported in Table S1.

	With hopanoids		No hopanoids	
Inhibitor	0.5 mol%	2.5 mol%	0.5 mol%	2.5 mol%
Formic acid	40.18 ± 0.60	39.89 ± 0.30	39.70 ± 0.05	38.62 ± 0.04
Acetic acid	40.32 ± 0.32	38.88 ± 0.32	39.27 ± 0.07	36.84 ± 0.01
Ethanol	40.30 ± 0.35	39.67 ± 0.25	39.48 ± 0.02	37.95 ± 0.03
Isobutanol	40.30 ± 0.29	35.62 ± 0.14	38.65 ± 0.02	34.41 ± 0.04

Table S6: Diffusion coefficient in units of 10^{-7} cm^2/s at two inhibitor concentrations with and without the hopanoid in membrane. The diffusion coefficient at 0 % solute concentration is reported in Table S1.

	With hopanoids		No hopanoids	
Inhibitor	0.5 mol%	2.5 mol%	0.5 mol%	2.5 mol%
Formic acid	1.49 ± 0.07	1.44 ± 0.12	2.05 ± 0.07	2.17 ± 0.02
Acetic acid	1.51 ± 0.06	1.47 ± 0.11	2.29 ± 0.22	2.48 ± 0.12
Ethanol	1.54 ± 0.19	1.78 ± 0.17	2.03 ± 0.05	2.28 ± 0.05
Isobutanol	1.46 ± 0.05	1.69 ± 0.01	2.15 ± 0.05	2.81 ± 0.03

Table S7: S_{CH} at two inhibitor concentrations with and without the hopanoid in membrane. The S_{CH} at 0 % solute concentration is reported in Table S1.

	With hopanoids		No hopanoids	
Inhibitor	0.5 mol%	2.5 mol%	0.5 mol%	2.5 mol%
Formic acid	0.1066 ± 0.0028	0.1046 ± 0.0029	0.1396 ± 0.0002	0.1290 ± 0.0004
Acetic acid	0.1087 ± 0.0016	0.0940 ± 0.0031	0.1358 ± 0.0002	0.1151 ± 0.0003
Ethanol	0.1051 ± 0.0039	0.1000 ± 0.0026	0.1373 ± 0.0006	0.1230 ± 0.0003
Isobutanol	0.1065 ± 0.0020	0.0757 ± 0.0009	0.1318 ± 0.0001	0.1027 ± 0.0003
